# Taming Bromine Azide for Use in Organic Solvents—Radical Bromoazidations and Alcohol Oxidations

**DOI:** 10.1021/acs.joc.2c03012

**Published:** 2023-02-23

**Authors:** Göran Schulz, Vincent George, Daghan Taser, Andreas Kirschning

**Affiliations:** Institute of Organic Chemistry, Leibniz University Hannover, Schneiderberg 1B, 30167 Hannover, Germany

## Abstract

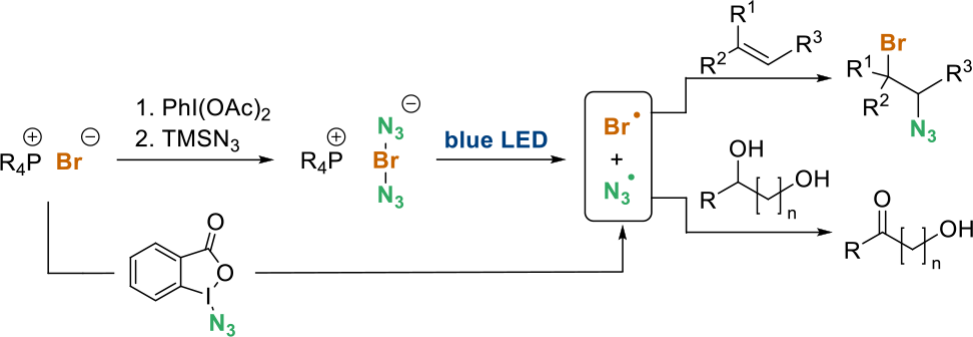

The formation of bromine azide from the bisazidobromate(I) anion or alternatively from Zhdankin’s reagent, using a phosphonium bromide salt as a common starting point, is reported. After homolytic cleavage in the presence of alkenes or alcohols either 1,2-functionalization or alternatively the selective oxidation of secondary alcohols in the presence of primary alcohols occur. The scopes and limitations of the use of BrN_3_ are covered.

## Introduction

The chemical potential of haloazides (X–N_3_; X = Cl, Br, I), whether ionically or radically induced, has not been fully exploited to date due to their highly explosive character (at Δ*p* ≥ 0.05 Torr; for X = Br: Δ*H*_explosion_ = −507 kcal kg^–1^, detonation temperature *T*_ex_= 6000 K and for X = I: Δ*H*_explosion_ = −805 kcal kg^–1^, detonation temperature *T*_ex_ = 6000 K). For X–N_3_ it was found that explosions can occur even with attempted crystallization. Dissociation is exothermic for CIN_3_, and BrN_3_, and mildly endothermic for IN_3_.^1^

One way to create a stable and storable form of iodine azide was reported by us that is obtained by iodine(III)-mediated oxidation of ammonium iodide.^2^ When acylated haloate(I) complexes^3^ are prepared from iodosobenzene diacetate (**2**), these anions can be further diversified by ligand exchange using silylated nucleophiles. When trimethylsilyl azide is employed, the bisazido iodate(I) anion is formed. It chemically behaves like iodine azide, which is assumed to be liberated from the bisazido iodate(I) anion.^4,5^ Under photochemical conditions this iodate(I) complex is homolytically cleaved so that the iodine and the azide radical are generated.^6^

**Scheme 1:**
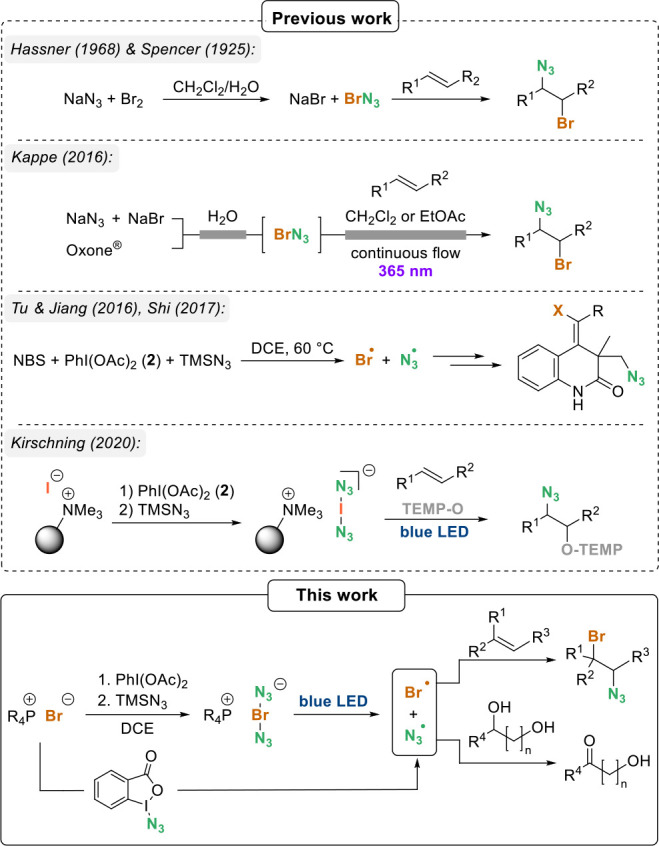
Development Bromine Azide Generation for Synthetic Applications

With these findings in mind, we aimed to extend our studies to related chemistry with the more explosive bromine azide (BrN_3_, **5**), especially as homolytic dissociation into bromine and azide radicals is more facile than for iodine azide.^2^ Because of the former property of BrN_3_ (**5**) this reagent has not become a common member in the portfolio of reagents of synthetic chemists. Bromine azide is usually generated by the reaction of sodium azide with Br_2_ in biphasic solvent systems such as CH_2_Cl_2_/H_2_O (Scheme 1).^7^ A technical approach to overcome the basic safety problems was achieved by switching from batch to continuous flow conditions as reported by Kappe and co-workers.^8^ In-situ formation was achieved by OXONE-mediated oxidation of a mixture of sodium bromide and sodium azide and continuously mixing with the organic substrate. Recently, Shi and co-workers disclosed a protocol describing the formation of BrN_3_ (**5**) from TMSN_3_, *N*-bromosuccinimide, and iodosobenzene diacetate (**2**) used in various radical chemistry applications.^9^

Apart from this work, very little attention has been focused toward the development of conditions that allow either ionic or radical reactions to be carried out with BrN_3_**5**. In this report, we have developed two approaches to this reagent that pave the way for conducting radical chemistry with BrN_3_ in a controlled manner.

## Result and Discussions

**Scheme 2:**
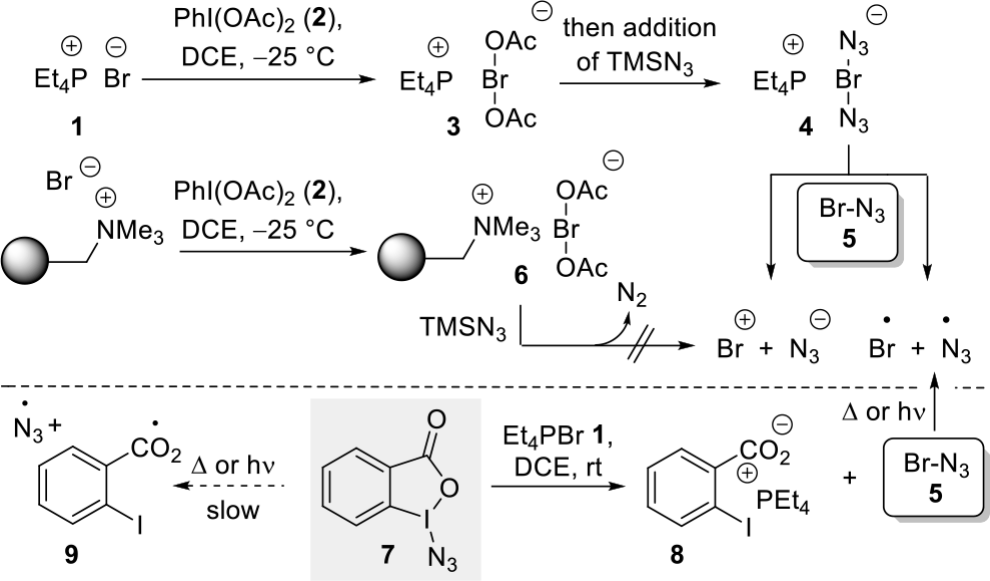
Preparation of Bisazido Bromate(I) Salt **4**, Formation of Bromine Azide (**5**), and Alternative Synthetic Approach Starting from Zhdankin’s Reagent (**7**) (for More Detailed Mechanistic Considerations on the Formation of Azide Radicals, See Scheme 8)

When transferring our protocol for the generation of polymer-bound bisazidoiodate(I) anions to the corresponding bromate(I) variant, we found that it did not work. Indeed, any attempt to produce bisazidobromate(I) anions (from **6**) resulted in immediate gas evolution (Scheme 2). Even when lower temperatures (−25 °C) were applied and the resulting polymer was added directly to indene **10a** at this temperature, the formation of a 1,2-adduct could not be detected, regardless of whether the reaction was irradiated with blue LED light or not.

For this reason, we investigated different new synthetic approaches in organic solvents to form bromine azide. A simple access to azide radicals via BrN_3_ would allow the study and broadening of the synthetic potential of this rarely used reagent. Despite the failures with the ion-exchange resin, we attempted to adhere to the concept of haloate(I) anions as a source of bromine azide. However, amonium-based counter cations were found not to be well suited as detailed in the Supporting Information (SI). In contrast, a practical access to bisazidobromate(I) anions such as **4** relies on phosphonium salt **1** as the bromide source.

In contrast to tetraethylphosphonium diacetoxybromate(I) (**3**), ate(I) anion **4** proved to rapidly liberate BrN_3_ (**5**) in dichloroethane (DCE) at −15 °C. Under irradiation with blue LED light, this process is already detectable at −25 °C.^10^ It is first oxidized to the bromate(I) anion **3** using iodosobenzene diacetate (**2**),^11^ and in the following formed at −25 °C by ligand exchange using trimethylsilyl azide (Scheme 2). Interestingly, we found that azido benziodoxolone (Zhdankin’s reagent, **7**) can also serve as an azide source for the formation of bromine azide (**5**), specifically after treatment with tetraethylphosphonium bromide (**1**).

**Scheme 3:**
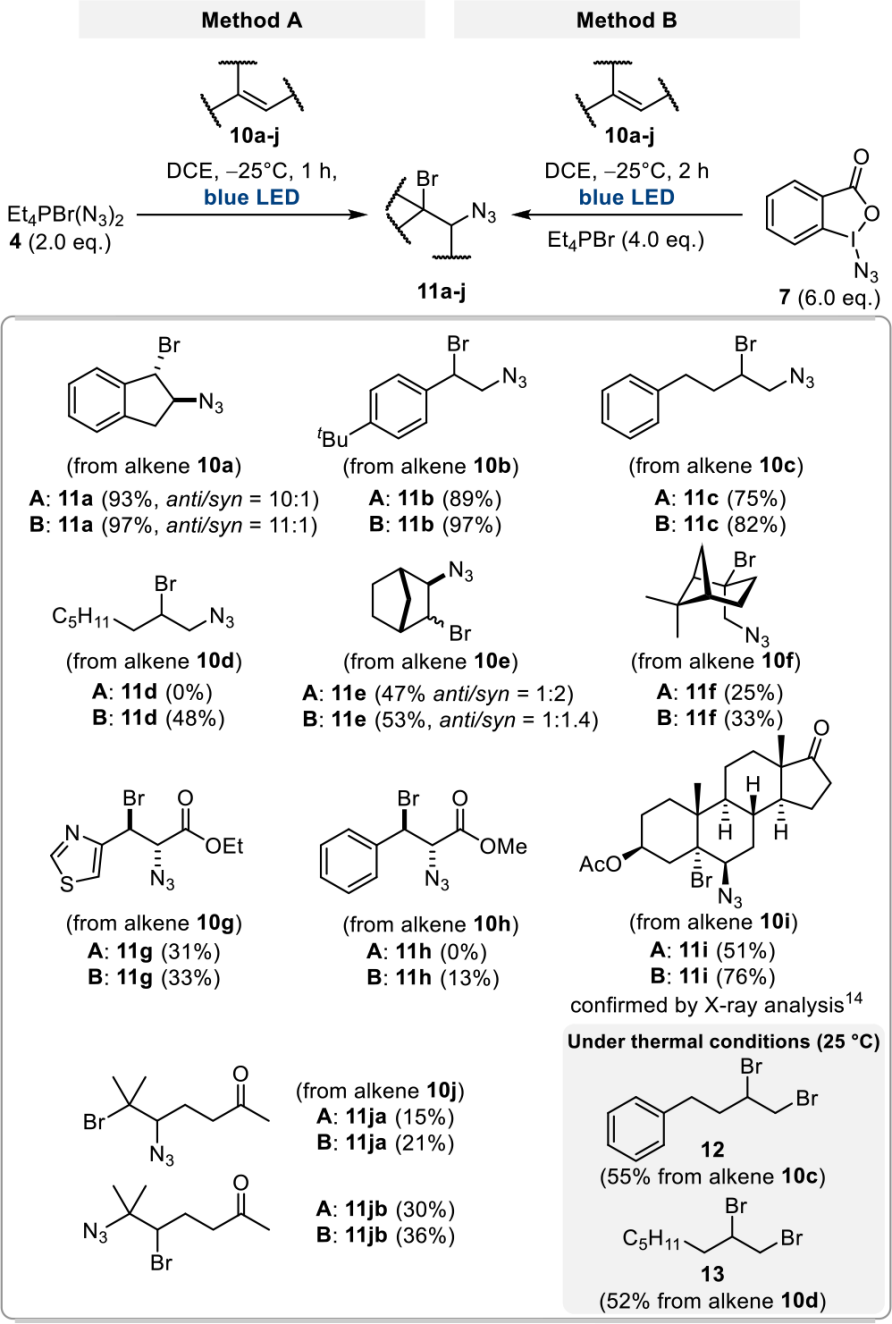
Methods A and B Are Suited for the *in Situ* Generation of BrN_3_ and Radical Bromoazidation of Alkenes **10a**–**j** under Photolytic Conditions; Dibromides **12** and **13** Are Formed from Alkenes **10c** and **10d**, Respectively, When Carrying out Method B under Thermal Conditions (25 °C)

Reagent **4** (method A, Scheme 3) or a mixture consisting of reagent **7** and bromide **1** (method B, Scheme 3) can be treated with alkenes under irradiation with blue LED light in DCE, and homolytic cleavage of the putative bromine azide (**5**) occurs. As a result, the azidobromination of alkenes takes place with opposite regioselectivity compared to the expected ionic 1,2-addition of BrN_3_. This is exemplified by the formation of 1,2-adducts **11a**–**11j**. Interestingly, radical bromoazidation can also be achieved with alkenes that are not of the styrene type, which has not been reported before. Thus, our method extends the scope of this type of 1,2-functionalization of alkenes. Importantly, we observed that electron-deficient alkenes **10g** and **10h** are not well suited for achieving high yields. When method B, which relies on Zhdankin’s reagent (**7**), was employed under thermal conditions (25 °C) we predominantly found the 1,2-dibromides (e.g., **12** and **13**) as exemplified for alkenes **10c** and **10d**, respectively.

In our view, method B, using the Zdhankin reagent as a starting point, leads to slightly better yields for radical 1,2 additions of BrN_3_ to alkenes than method A (see details in Scheme 8).

**Scheme 4:**
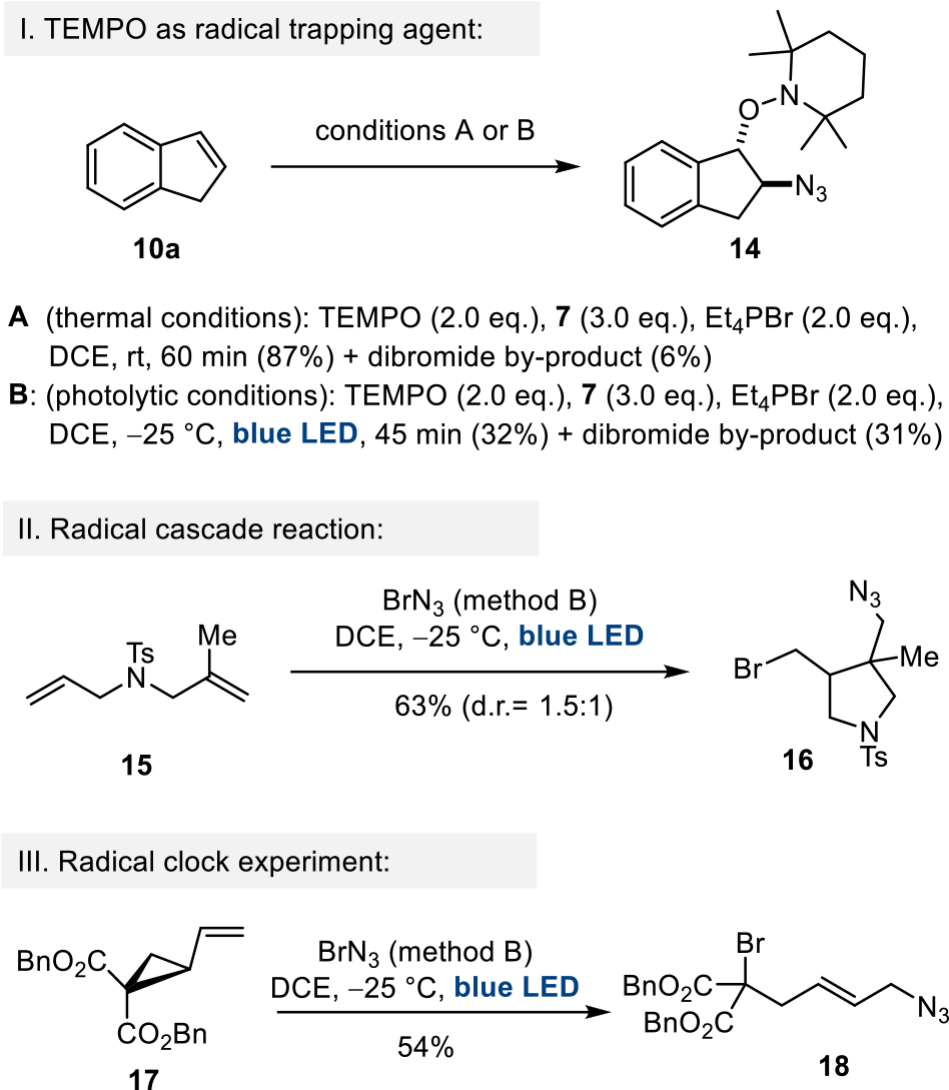
Three Experiments to Shed Light on the Mechanism: (I) Azidooxygenation of Alkene **10a** in the Presence of TEMPO; (II) Radical Cascade Experiment with Diene **15**; (III) Radical Clock Experiment Using Vinylcyclopropane **17**

Three additional experiments were selected to confirm that a radical mechanism is present. First, we repeated the bromoazidation with alkene **10a** using method B and added the radical scavenger 2,2,6,6-tetramethylpiperidinyloxy (TEMPO) to the reaction mixture (Scheme 4I). Studer et al.^12^ had previously described the azidooxygenation of alkenes using Zdhankin’s reagent and in situ formed TEMPO (from TEMPONa), but in the absence of a bromide source such as phosphonium salt **1**. Another way to achieve azidooxygenation of alkenes is by employing the explosive, in situ formed PhI(N_3_)_2_ and the TEMPO radical.^13^

In the presence of **1**, we isolated 1,2-adduct **14** rather than 1,2-bromoazide **11a**, irrespective whether the 1,2-addition was carried out under thermal or photochemical conditions. Obviously, the radical, formed upon addition of the azide radical to the alkene, was rapidly captured by TEMPO and not by a bromine radical. It also confirms that BrN_3_ (**5**) commonly dissociates homolytically.^1^ In the second experiment, diene **15** was chosen as a tool for detecting radical formation. It served as a starting point for a radical cascade reaction involving cyclization initiated by the reaction of the initially formed C radical with the second olefinic double bond (Scheme 4II). Indeed, the reaction provided cyclopentane derivative **16** in good yield. Third, we performed a radical clock experiment with vinylcycylopropane **17**, and again the formation of allyl azide **18** after treatment with BrN_3_ (**5**) under photolytic conditions revealed the presence of a radical mechanism (Scheme 4III).

**Scheme 5:**
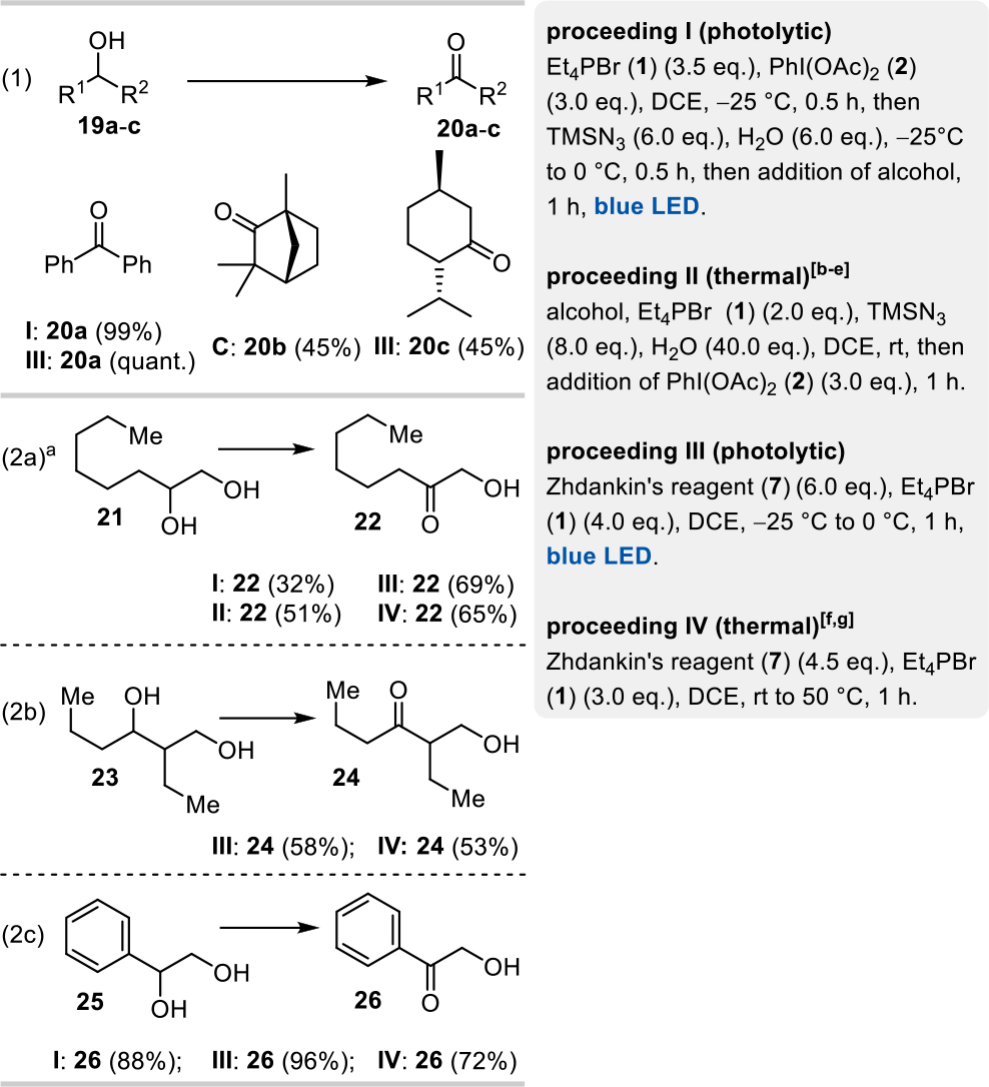
Oxidation of Secondary Alcohols **19a**–**c** and Diols **21**, **23**, and **25** (Isolated Yields Are Reported) Details on optimization of reaction parameters are found in the SI. Yield: 0 °C < 25 °C > 60 °C. Solvent dependency: DCE > MeCN > DME. Additives: Et_3_N inhibits the reaction, exchange of TMSN_3_ by Bu_4_NN_3_ leads to reduced yields, and Lewis acids like Zn(OTf)_2_ or CuOTf did not have a significant effect on the yield. Yield: PhI(OTs)(OH) ≈ PhI(OAc)_2_ > PhI(CO_2_CCF_3_)_2_ > PhIO. Additives: exchange of Et_4_PBr by NBS leads to reduced yield; addition of CuCl did not have a significant effect on the yield. Solvent dependency: DCE > PhCF_3_.

In our previous work, we showed that iodine azide is capable of chemoselectively oxidizing secondary alcohols in the presence of a primary alcohol under photolytic conditions. This reactivity had been unknown up to that point. Our studies provided strong evidence that this oxidation proceeds via a radical mechanism. With azide radical formation from in situ generated BrN_3_, removal of a H radical from the C atom of the carbinol moiety takes place first. As a consequence we tested the use of bromine azide for such kind of oxidations as it is better suited to induce radical processes than IN_3_ (Scheme 5).

To avoid the well-known *O*-silylation of alcohols mediated by TMSN_3_ and a bromide source,^14^ we decided to add water. However, this procedure required the reaction mixture to be warmed up from −25 to 0 °C, which was accompanied by a more rapid decomposition of the bisazidobromate(I) anion. This manifested itself in only a minor degree of converted diol **21** (Scheme 5, entry 2a, proceeding I), albeit with the desired selectivity for the secondary alcohol. Therefore, we were prompted to test further conditions under thermal and photolytical conditions (see SI for details). With the optimized proceeding III using Zhdankin’s reagent (**7**) as azide source and activation by blue LED light, we succeeded in obtaining hydroxyketone **22** in 69% yield.

For activated secondary alcohols such as **19a** and **25**, the oxidations proceeded in nearly quantitative yield (Scheme 5, entries 1 and 2c). In contrast, nonactivated secondary alcohols **19b** and **19c** are not well suited for these chemoselective oxidations from a preparative point of view.

The yield could not be significantly increased by longer reaction times or the addition of further reagents. One assumption for this relates to a possible reaction-inhibiting effect of the product formed. Therefore, the oxidation was carried out with a 1:1 mixture of diol **21** and hydroxyketone **22** (see SI), but no fundamentally different result was encountered supporting the assumption. However, the cause of the inhibitory effect is unknown at this point.

**Scheme 6:**
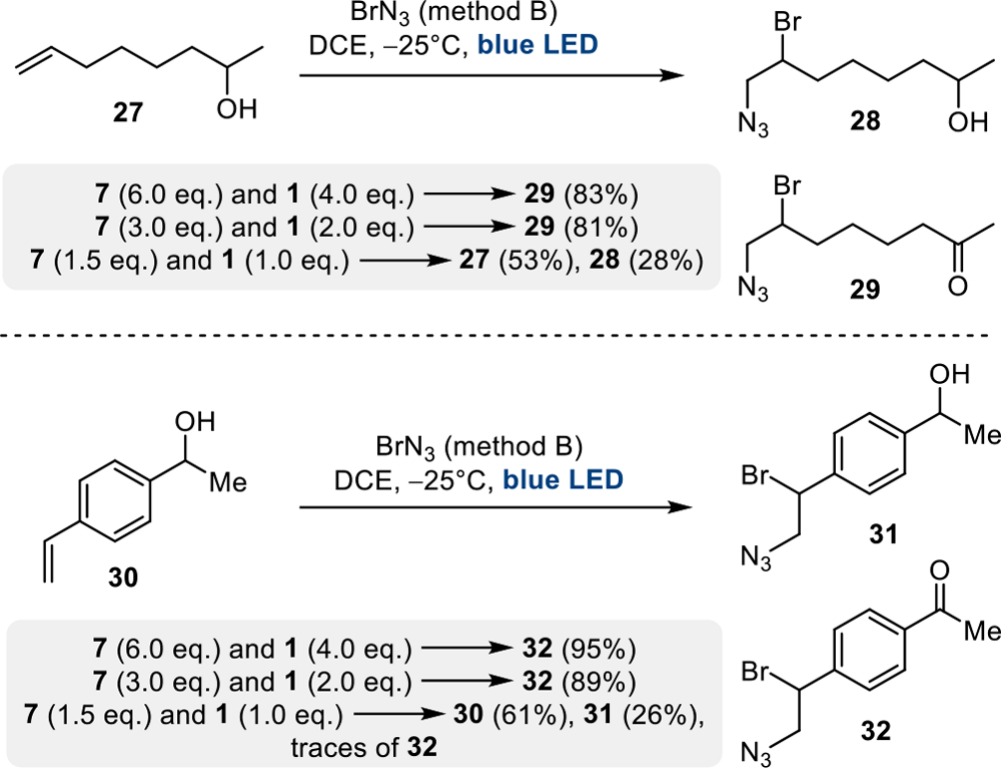
Chemoselectivity Studies on Bifunctionalized Model Substrates **27** and **30**

**Scheme 7:**
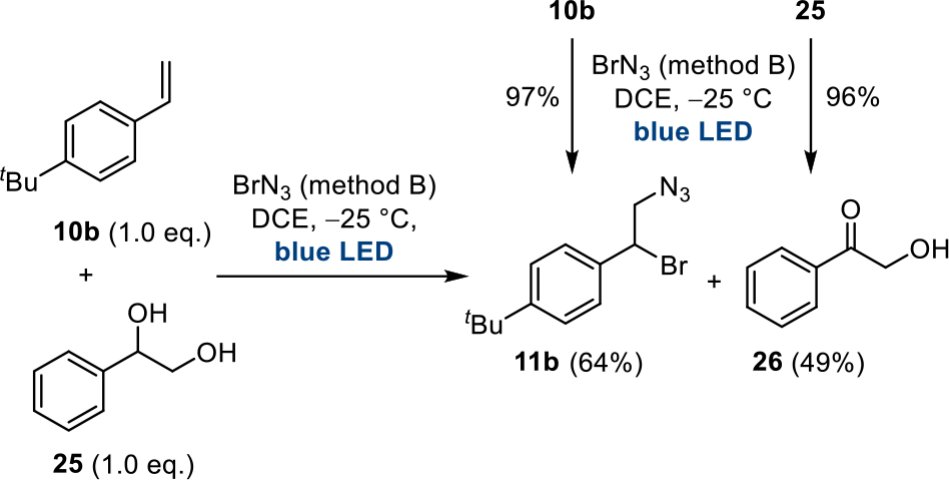
Chemoselectivity Studies by a Competition Experiment

After examining two basic reactions, the bromoazidation and the oxidation of alcohols, we turned our attention to the question of chemoselectivity. Method B for the formation of BrN_3_ was applied to substrates **27** and **30** containing both an alkene moiety and a secondary alcohol. We observed the preferential formation of the 1,2-adducts **29** and **32**, respectively, that had also undergone alcohol oxidation. However, when the number of equivalents of active BrN_3_ was reduced, the bromoazidation product was formed primarily (Scheme 6). Related to this is a competition experiment that starts with a mixture of an alkene and a secondary alcohol in the presence of a BrN_3_ source (Scheme 7). We chose substrates **10b** and **25**, which had already been employed for 1,2-bromozidations or oxidations of alcohols, and subjected the mixture to method B. Analysis of the products revealed that low chemoselectivity with a preferred trend for bromoazidation was observed.

**Scheme 8:**
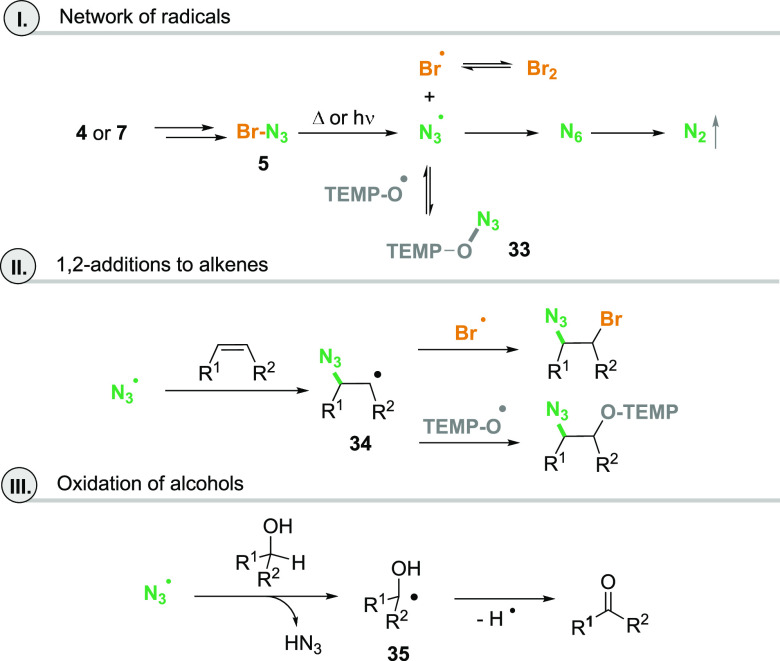
Proposed Mechanistic Considerations on Radical Processes Reported Here (for **33** One Possible Structure Is Shown)

From a mechanistic point of view, for BrN_3_ (**5**), unlike for IN_3_, the question does not arise under which conditions homolytic cleavage occurs as it can be achieved under both thermal and photolytic conditions. However, the stability at room temperature seems to be much lower than at −25 °C and decomposition occurs rapidly after its formation. For this reason, photolytically induced azide radical formation at lower temperatures is the method of choice. However, we noted that with respect to Zdhankin’s reagent **7**spontaneous homolysis of the hypervalent iodine-azide bond very likely does not occur. In fact, it only rapidly forms the known dark red-brownish charge transfer complex TEMPO-N_3_**33**^16^ with TEMPO in the presence of a bromide source (Scheme 8 I and SI) while bromide strongly facilitates the cleavage of the azido ligand from **7**. Indeed, it was reported that homolysis of the hypervalent iodine–azide bond in Zhdankin’s reagent is observed only at elevated temperatures (<40 °C)^17^ or is induced by a single-electron oxidant.^18^ Finally, recording of UV–vis spectra and comparison with literature data (λ_max_ = 292 nm)^19^ provided additional indications that BrN_3_ is formed after treatment of Zdhankin’s reagent with **1** (see SI).

Nevertheless, once BrN_3_ has formed, homolytic cleavage yields Br and N_3_ radicals, which can combine to form molecular bromine and N_6_, and the latter spontaneously decomposes to N_2_ (Scheme 8II).^15^ Bromine remains, which then can undergo 1,2-additions to alkenes (see products **12** and **13**; Scheme 3). In the presence of the TEMPO radical the azide radical can be trapped to form the metastable charge-transfer complex **33** (TEMPO^+^/N_3_^–^) which is in equilibrium with the precursor radicals.^16^ As such it may serve as an azide radical reservoir.

At −25 °C the 1,2-bromoazidation of alkenes is preferred likely because sufficient amounts are available (Scheme 8III). In the presence of the TEMPO radical the intermediate radical **34** is preferentially trapped by TEMPO over the bromine radical. The azide radical also enforces C–H abstraction next to a C–O bond as in secondary alcohols and the corresponding ketones are formed via the ketyl radicals **35** as recently established by us for iodine azide (Scheme 8IV).^6^

Nevertheless, once BrN_3_ has formed, homolytic cleavage yields Br and N_3_ radicals, which can combine to form molecular bromine and N_6_, with the latter spontaneously decomposing to N_2_ (Scheme 8II).^15^ Bromine remains, which then can undergo 1,2-additions to alkenes (see products **12** and **13**; Scheme 3). In the presence of the TEMPO radical the azide radical can be trapped to form the metastable charge-transfer complex **33** (TEMPO^+^/N_3_^–^) which is in equilibrium with the precursor radicals.^16^ As such it may serve as an azide radical reservoir.

## Conclusions

In conclusion, we have shown that BrN_3_ can be generated by two new routes, homolytically cleaved in organic solvents such as DCE and reacted with both alkenes and secondary alcohols under the regime of radicals. These preliminary studies suggest that Zdhankin’s reagent (**7**) is a better starting point than the haloate(I) route to utilize BrN_3_ in radical reactions. Our investigations suggest that the release of the azide radical from the mixture of Zhdankin’s reagent and Et_4_PBr and formation of BrN_3_ is more controlled than BrN_3_ generation from the reaction mixture composed of Et_4_PBr(OAc)_2_ and TMSN_3_. As a consequence, the formation of molecular bromine and nitrogen is less likely due to the lower radical concentration. This assumption is also supported by the observation that the photochemical experiments at lower temperatures proceeded with better yields than the thermal experiments at temperatures well above 0 °C. The scope of preparative choices for the use of bromine azide has been expanded, although the universality of bromoazidations as well as selective oxidations of secondary alcohols has not yet been fully achieved. However, we believe that we have succeeded in further advancing the acceptance of bromine azide as a reagent in organic synthesis.

## Experimental Section

### ***CAUTION***

Despite the fact that we found the procedures reported to be safe, we stress that BrN_3_ is potentially explosive. Precautions should be taken.

### General Procedures

#### Method A: Selective 1,2-Bromoazidation of Olefins Using PhI(OAc)_2_, TMSN_3_, and Et_4_PBr under Blue LED Light Irradiation

A suspension of PhI(OAc)_2_ (145 mg, 450 μmol, 1.50 equiv) in dry DCE (6.00 mL) was cooled to −25 °C under argon. Et_4_PBr (136 mg, 600 μmol, 2.00 equiv) was added, and stirring continued for 30 min at −25 °C. Then TMSN_3_ (138 μL, 1.05 mmol, 3.50 equiv) was added, and the mixture stirred for an additional 30 min at −25 °C. The alkene (300 μmol, 1.00 equiv) was added, and the mixture was irradiated with a blue LED. The reaction was monitored by thin layer chromatography and terminated by addition of Na_2_S_2_O_3_ solution (aq., sat.). The aqueous phase was separated and washed with CH_2_Cl_2_ (2×). The combined organic layers were dried over Na_2_SO_4_ and dried in vacuo to give the crude product, which was purified by flash column chromatography.

#### Method B: Selective 1,2-Bromoazidation of Olefins Using 1-Azido-1,2-benziodoxol-3(1*H*)-one (Zhdankin’s Reagent) and Et_4_PBr under Blue LED Light Irradiation

To a suspension of Zhdankin’s reagent (520 mg, 1.80 mmol, 6.00 equiv) in DCE (5.00 mL) was added Et_4_PBr (273 mg, 1.20 mmol, 4.00 equiv) at −25 °C, and the resulting mixture stirred for 20 min. A solution of the alkene (300 μmol, 1.00 equiv) in DCE (1.00 mL) was then added to the orange suspension and stirred at −25 °C for 2 h. The reaction was stopped by addition of Na_2_S_2_O_3_ solution (aq., sat.), and the separated aqueous phase extracted with CH_2_Cl_2_ (2×). The combined organic phases were subsequently washed with NaHCO_3_ solution (aq., sat.) and after separation of the layers the aqueous phase extracted with CH_2_Cl_2_ (2×). The organic phases were combined, and after purification by column chromatography, the product was isolated.

#### Method C: Selective 1,2-Bromoazidation of Olefins Using 1-Azido-1,2-benziodoxol-3(1*H*)-one (Zhdankin’s Reagent) and Et_4_PBr without LED Light Irradiation

To a mixture of Et_4_PBr (273 mg, 1.20 mmol, 4.00 equiv) and alkene (300 μmol, 1.00 equiv) in DCE (5.00 mL) Zhdankin’s reagent (520 mg, 1.80 mmol, 6.00 equiv) was portionwise added over 30 min at room temperature; gas formation and a color change from yellow to orange were clearly observed. The suspension was then stirred for further 1.5 h at room temperature, the reaction was stopped by addition of Na_2_S_2_O_3_ solution (aq., sat.), and the separated aqueous phase was extracted with CH_2_Cl_2_ (2×). The combined organic phases were subsequently washed with NaHCO_3_ solution (aq., sat.), and after separation of of the layers, the aqueous phase was extracted with CH_2_Cl_2_ (2×). The organic phases were combined, and after purification by column chromatography, the product was isolated.

#### Proceeding I: Selective Oxidation of Secondary Alcohols Using PhI(OAc)_2_, TMSN_3_, and Et_4_PBr under Blue LED Light Irradiation

A suspension of PhI(OAc)_2_ (291 mg, 900 μmol, 3.00 equiv) in dry DCE (12.0 mL) was cooled to −25 °C under an argon atmosphere. Et_4_PBr (239 mg, 1.05 mmol, 3.50 equiv) was added, and stirring continued for 30 min at −25 °C. TMSN_3_ (236 μL, 1.81 mmol, 6.00 equiv) was added, followed by water (32.5 μL, 1.81 mmol, 6.00 equiv), and the mixture was stirred for an additional 30 min at −25 °C. Then, the alcohol (300 μmol, 1.00 equiv) was added, and the mixture was irradiated with blue LED light and allowed to warm to 0 °C over a period of 1 h. Subsequently, the reaction was terminated by addition of Na_2_S_2_O_3_ solution (aq., sat.). The phases were separated, and the aqueous phase was extracted with CH_2_Cl_2_ (2×). The combined organic layers were dried over Na_2_SO_4_, filtered, and concentrated under reduced pressure to give the crude product, which was purified by flash column chromatography.

#### Proceeding II: Selective Oxidation of Secondary Alcohols Using PhI(OAc)_2_, TMSN_3_, and Et_4_PBr without LED Light Irradiation

A solution of the alcohol (300 μmol, 1.00 equiv) in DCE (3.75 mL) was treated with TMSN_3_ (314 μL, 2.40 mmol, 8.00 equiv), Et_4_PBr (136 mg, 600 μmol, 2.00 equiv), and water (216 μL, 12.0 mmol, 40.0 equiv) at room temperature. Then PhI(OAc)_2_ (290 mg, 900 μmol, 3.00 equiv) was added portionwise over 30 min. When the solid was added, nitrogen formation and a yellow coloration of the solution became apparent, which disappeared after a few minutes. After complete addition, the mixture was stirred for an additional 30 min before the reaction was terminated by the addition of Na_2_S_2_O_3_ solution (aq., sat.). The phases were separated, and the aqueous phase was extracted with CH_2_Cl_2_ (2×). The combined organic layers were dried over Na_2_SO_4_, filtered, and concentrated under reduced pressure to give the crude product, which was purified by flash column chromatography.

#### Proceeding III: Selective Oxidation of Secondary Alcohols Using 1-Azido-1,2-benziodoxol-3(1*H*)-one (Zhdankin’s Reagent) and Et_4_PBr under Blue LED Light Irradiation

A solution of the alcohol (300 μmol, 1.00 equiv) in DCE (6.00 mL) was treated with 1-azido-1,2-benziodoxol-3(1*H*)-one (522 mg, 1.81 mmol, 6.00 equiv) and Et_4_PBr (273 mg, 1.20 mmol, 4.00 equiv) at −25 °C under an argon atmosphere. Then, the mixture was irradiated with blue LED light and allowed to warm to 0 °C over a period of 1 h. Subsequently, the reaction was terminated by addition of Na_2_S_2_O_3_ solution (aq., sat.) and K_2_CO_3_ solution (aq., 10 wt %). The phases were separated, and the aqueous phase was extracted with CH_2_Cl_2_ (2×). The combined organic layers were dried over Na_2_SO_4_, filtered, and concentrated under reduced pressure to give the crude product, which was purified by flash column chromatography.

#### Proceeding IV: Selective Oxidation of Secondary Alcohols Using 1-Azido-1,2-benziodoxol-3(1*H*)-one (Zhdankin’s Reagent) and Et_4_PBr without LED Light Irradiation

A suspension of the alcohol (300 μmol, 1.00 equiv) and 1-azido-1,2-benziodoxol-3(1*H*)-one (390 mg, 1.35 mmol, 4.50 equiv) in DCE (3.75 mL) was treated with and Et_4_PBr (204 mg, 900 μmol, 3.00 equiv) at room temperature under an argon atmosphere. The mixture was then stirred at 50 °C for 1 h, during which time the formation of nitrogen bubbles can be observed. Subsequently, the reaction was terminated by addition of Na_2_S_2_O_3_ solution (aq., sat.) and K_2_CO_3_ solution (aq., 10 wt %). The phases were separated, and the aqueous phase was extracted with CH_2_Cl_2_ (2×). The combined organic layers were dried over Na_2_SO_4_, filtered, and concentrated under reduced pressure to give the crude product, which was purified by flash column chromatography.

## Data Availability

The data underlying this study are available in the published article and its Supporting Information.

## Supporting Information Available

It describes individual experiments and covers copies of ^1^H, ^13^C{^1^H } and 2D-NMR and IR spectra. (PDF)
