# Supplementary material for: Taming Bromine Azide for Use in Organic Solvents—Radical Bromoazidations and Alcohol Oxidations
Source: J Org Chem. 2023 Feb 23;88(6):3781–6. doi: 10.1021/acs.joc.2c03012 (PMC10028602; doi:10.1021/acs.joc.2c03012)
Supplement: Supplementary file 1 — jo2c03012_si_001.pdf [file jo2c03012_si_001.pdf]

Supporting Information for

TAMING BROMINE AZIDE FOR USE IN ORGANIC SOLVENTS – RADICAL BROMOAZIDATIONS AND ALCOHOL OXIDATIONS

Göran Schulz,‡ Vincent George,‡ Daghan Taser,‡ Andreas Kirschning*

Institute of Organic Chemistry, Leibniz University Hannover,

Schneiderberg 1b, 30167 Hannover

* E-Mail: andreas.kirschning@oci.uni-hannover.de

Table of Contents

1. Materials and methods S3
   1.1. General information S3
   1.2. Set-up for photo-activated reactions S4
   1.3. Statement concerning safety issues S4
2. Optimization studies S5
   2.1. Studies on the temperature-dependent release of azide radicals from BrN_3 precursors S5
   2.2. Optimization studies on the bromoazidation of double bonds according to method A S6
   2.3. Optimization studies on the bromoazidation of double bonds regarding the bromide source according to method A S7
   2.4. Optimization studies on the selective oxidation of secondary alcohols using $PhI(OAc)_2$, $TMSN_3$ and Et_4PBr without LED light irradiation S8
   2.5. Optimization studies on the selective oxidation of secondary alcohols using 1-azido-1,2-benziodoxol-3(1*H*)-one (Zhdankin's reagent) and Et_4PBr S9
3. Chemical syntheses S10
   3.1. Selective 1,2-bromoazidation of olefins S10
   3.2. Selective oxidation of secondary alcohols S16
   3.3. Mechanistic studies S19
   3.4. Chemoselectivity studies S23
4. X-ray crystal structure analysis of compound 10i S26
5. References S29
6. Attachments: ^{1}H NMR, $^{13}C\{^{1}H\}$ NMR and IR spectra S31

1. Materials and methods

1.1. General information

^{1}H NMR spectra were recorded at 400 MHz with a BRUKER Avance-400 and BRUKER Ascend-400 at 323K. ^{13}C NMR spectra were recorded at 101 MHz with a BRUKER Avance-400 and BRUKER Ascend-400. Multiplicities are described using the following abbreviations: s = singlet, d = doublet, t = triplet, q = quartet, sex = sextet, qd = quartet of doublet, tq = triplet of quartet, m = multiplet, br = broad. Substitutions of carbons are described using the following abbreviations: p = primary, s = secondary, t = tertiary, q = quaternary. Chemical shift values of ^{1}H and ^{13}C NMR spectra are commonly reported in ppm relative to residual solvent signal as internal standard. The multiplicities refer to the resonances in the off-resonance decoupled spectra and were elucidated using phase-sensitive HSQC experiments.

Mass spectra were obtained with a lockspray dual ion source in combination with a WATERS Alliance 2695 LC system, or with a type Q-TOF premier (MICROMASS) spectrometer (ESI mode) in combination with a WATERS Acquity UPLC system equipped with a WATERS Acquity UPLC BEH C18 1.7 µm (SN 01473711315545) column (solvent A: water + 0.1 % {v/v} formic acid, solvent B: MeCN or MeOH {given in experimental part} + 0.1 % {v/v} formic acid; flow rate = 0.4 mL/min; gradient {t [min]/solvent B [%]}: {0/5} {2.5/95} {6.5/95} {6.6/5} {8/5}; retention times {r_t} given in the experimental part). Ion mass signals (*m/z*) are reported as values in atomic mass units.

High-resolution mass spectrometry (HRMS) was measured with a Micromass LCT with lockspray source. The injection proceeded in loop-mode with a HPLC system by WATERS (Alliance 2695). Alternatively, mass spectra were recorded with an Acquity-UPLC system by WATERS in combination with a Q-Tof Premier mass spectrometer by WATERS in lockspray mode. The ionization happened by electrospray ionization (ESI) or by chemical ionization at atmospheric pressure (APCI). The calculated and found mass are reported. GC/MS analyses were carried out with an HP 6890 chromatograph with KAS 4, coupled to an HP 5973 quadrupole mass selective detector. Samples were analyzed on an OPTIMA 5 column (poly(5%-phenyl-95%-methylsiloxane), 30 m x 0.32 mm i.d. x film thickness 0.25 µm). Carrier gas, He; injector temp., 60 °C to 300 °C at 12 °C/min, splitless; temp. program: 50 °C (isothermal 1 min) to 300 °C, at 20 °C/min and held isothermal for 6 min at 300 °C; ion source: EI, ionization energy, 70 eV; electron mass spectra were acquired over the mass range of 40 – 500 amu.

Analytical thin-layer chromatography was performed using precoated silica gel plates (MACHERY NAGEL, DÜREN) and the spots were visualized with UV light at 254 nm or alternatively by staining with permanganate or 4-methoxybenzaldehyde solutions.

Commercially available reagents, chromatography type or dry solvents were used as received or purified by standard techniques according to the literature.[S1] 1-Azido-1,2-benziodoxol-3(1*H*)-one (Zhdankin's reagent) (**7**)[S2], ethyl (*E*)-3-(thiazol-4-yl)acrylate (**10g**)[S3], *N*-allyl-4-methyl-*N*-(2-methylallyl)benzenesulfonamide (**15**)[S4], dibenzyl 2-vinylcyclopropane-1,1-dicarboxylate (**17**)[S5], oct-7-en-2-ol (**27**)[S6] and 1-(4-vinylphenyl)ethan-1-ol (**30**)[S7] were synthesized according to literature-know procedures.

Flash column chromatography was performed using mesh silica by MACHERY NAGEL (grain size 40-63 μm), with the indicated solvent system according to the standard techniques.

Infrared spectra (ν_{max}, FTIR) were recorded in reciprocal centimeters (cm^{-1}) as thin films or compressed solids on a SHIMADZU FT-IR Affinity-1S spectrometer. Melting points were determined on an SRS OptiMelt apparatus and are not corrected.

Specific Optical rotation values $[\alpha]^T_D$ were measured in a quartz cuvette on a polarimeter 341 by PERKINELMER at a wavelength of 589 nm (D) and given temperature *T*.

X-ray structure analysis was performed using a BRUKER SMART X2S benchtop crystallographic system utilizing UCSF Chimera (version 1.14) software for visualisation.

1.2. Set-up for photo-activated reactions

All photo-activated reactions were carried out in a cryostat cooled 2-propanol bath. A 5 m COB LED strip with 224 LED/m, 10 W*m^{-1} and wavelengths of λ = 440 nm – 495 nm was used as the light source. The strip was partially submerged to ensure optimal irradiation conditions. The temperature of the cooling bath was controlled by an external thermometer.

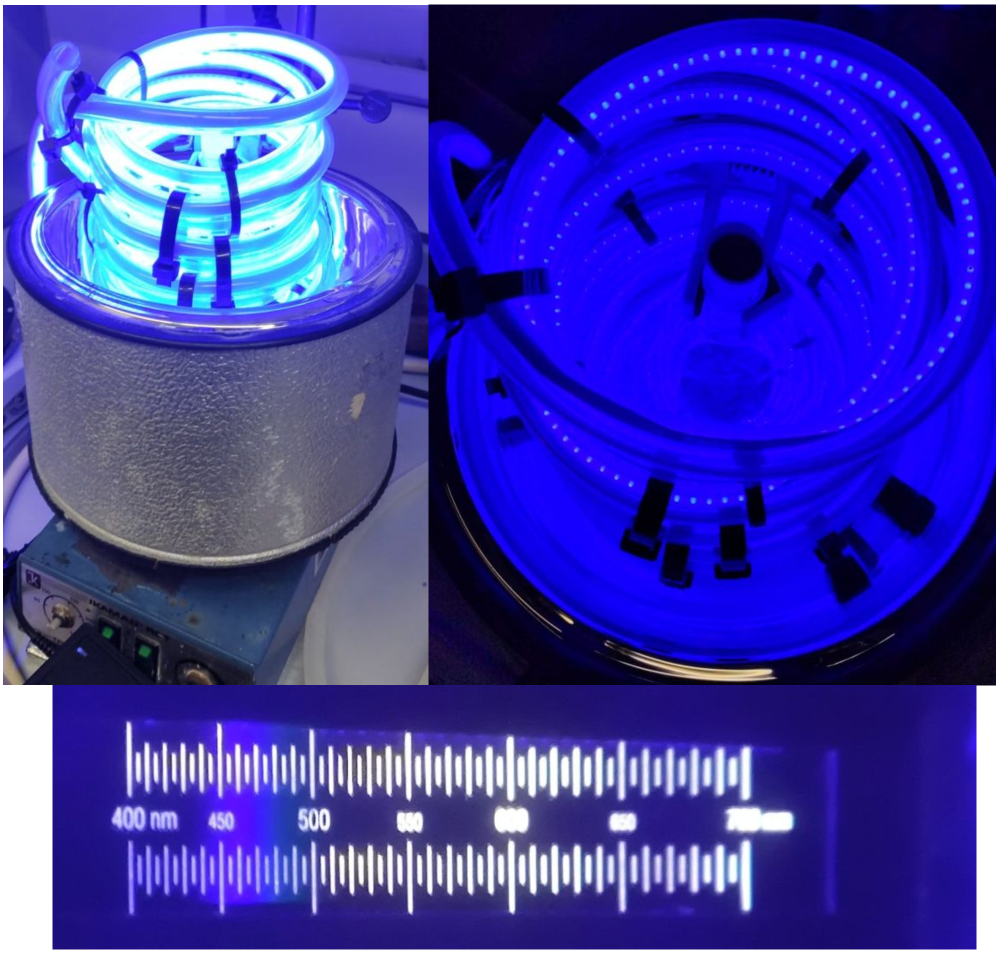

Figure S1: Set-up for photo-activated reactions.

1.3. Statement concerning safety issues

Organic azides are potentially explosive compounds and appropriate safety protocols to be observed, as they decompose with the slight input of energy from external sources as light, heat, pressure, etc.[S8] 1-Azido-1,2-benziodoxol-3(1*H*)-one (Zhdankin's reagent) (**7**) shows a high shock and friction sensitivity and should be handled with care.[S9]

Using our protocols, we have never experienced a safety problem.

2. Optimization studies

2.1. Studies on the temperature-dependent release of azide radicals from BrN_3 precursors

Azide radicals form a dark brown charge-transfer complex with TEMPO radicals. The clearly visible color change from light yellow to the dark complex thus enables studies on the temperature-dependent release of azide radicals from the bromine azide precursors. At 32 °C, a rapid color change and gas evolution is evident with both methods, although the rate of gas evolution is much greater with the method based on the bisazidobromate(I) species. At lower temperatures, the color change occurs much more slowly with the Zhdankin's reagent-based method. When cooled to −25 °C, the color of both mixtures did not change after 5 minutes, but irradiation with blue light led to darkening. At −35 °C, even with irradiation, it took a long time for the color to change, and the mixtures did not become as dark as the others.

Table S1: Studies on the temperature-dependent release of azide radicals from bromine azide precursors by observing gas evolution and formation of a brown TEMPO-N_3 complex.

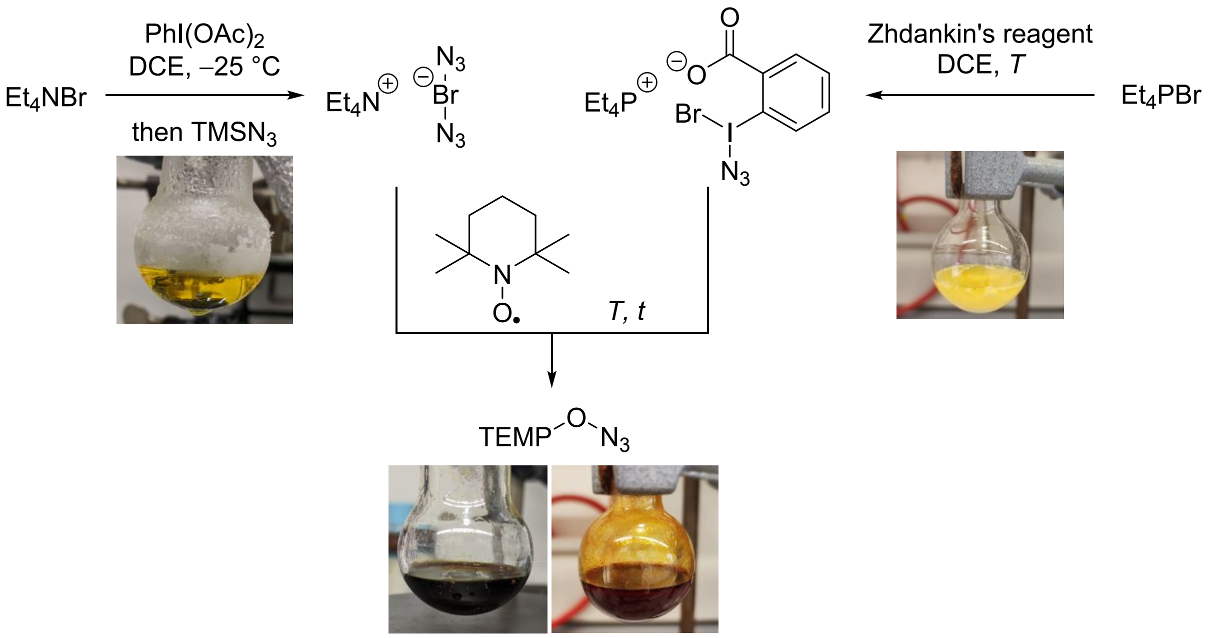

Entry	T / °C	$Et_4NBr/PhI(OAc)_2/TMSN_3$		Et_4NBr/Zhdankin's reagent	
		Gas evolution	t_{change} / s	Gas evolution	t_{change} / s
1	32	++	1	+	1
2	0	+	10	–	35
3	–16	–	120	–	180
4	–25	–	>300 / 10[1]	–	>300 / 150[1]
5	–35	–	>300[1]	–	>300[1]

[1]irradiation with blue LED light

2.2. Optimization studies on the bromoazidation of double bonds according to method A

Table S2: Optimization studies on the bromoazidation of double bonds according to method A.

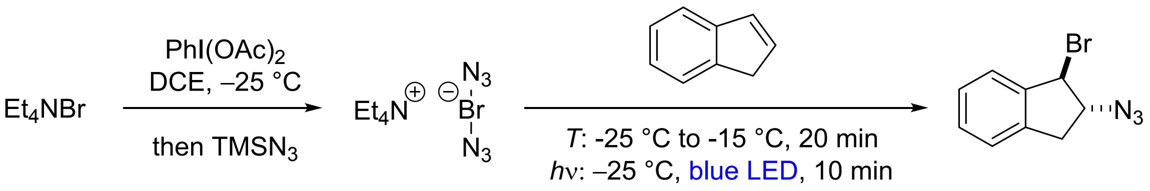

Entry	Et_4NBr /eq.	$PhI(OAc)_2$ / eq.	$TMSN_3$ /eq.	Method	*trans*:*cis*[1]	Yield[2] / %
1	1.50	2.00	4.00	*T*	n.d.	(63)
2	1.50	2.00	4.00	*hv*	n.d.	(62)
3	3.00	5.00	8.00	*hv*	n.d.	(42)
4	3.00	5.00	8.00	*T*	n.d.	(61)
5[4]	1.50	2.00	4.00	*hv*	8:1	65
6[5]	1.50	1.75	3.50	*hv*	3:2	49
7	2.00	1.50	3.50	*hv*	9:1	79 (74)
8	3.00	1.50	2.80	*hv*	8:1	65 (63)
9	2.00	1.50	3.50	*T*	62	63 (62)

[1]Determined by ^{1}H-NMR spectroscopy; [2] ^{1}H NMR-yields of the *trans*-product using naphthalene as an internal standard; yields in brackets are isolated yields; [4]reaction was started at –40 °C and heated up to –20 °C during 40 min of irradiation; [5]$PhI(O_2CCF_3)_2$ instead of $PhI(OAc)_2$. Conditions: indene (300 µmol, 1.00 eq.), Et_4NBr, $PhI(OAc)_2$, $TMSN_3$ in dry DCE (6.00 mL) under argon atm.

2.3. Optimization studies on the bromoazidation of double bonds regarding the bromide source according to method A

According to our observations, the stability of the bisazido bromate(I) salts does not depend in principal on the cation chosen. However, in azidobrominations with indene **10a**, we observed a trend related to the solubility of the bromide salt. Yields and selectivities were generally better when the bromide was less soluble in the DCE (relative yields: $MePh_3P$ < Oct_4N < Bu_4N < Et_4N < Et_4P). However, if the solubility is too low, no conversion can be observed (Me_4N and Me_2Dod_2N).

Table S3: Optimization studies on the bromoazidation of double bonds regarding the bromide source according to method A.

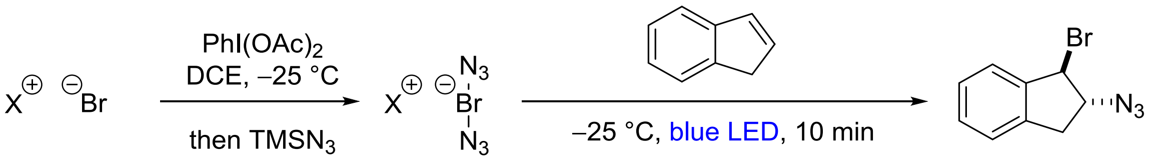

Entry	X^+	Yield / %[1]	*trans*:*cis*[2]
1	Me_4N^+	-	-
2	Et_4N^+	79	9:1
3	Bu_4N^+	47	5:1
4	Oct_4N^+	53	4:1
5	$Me_2Dod_2N^+$	-	-
6	$MePh_3P^+$	<45[3]	2:1
7	Et_4P^+	86	10:1

[1] ^{1}H NMR-yields of the *trans*-product using naphthalene as an internal standard; [2]determined with ^{1}H-NMR spectroscopy; [3]yield could not be accurately determined because the aromatic signals of the salt overlapped with those of indene and product in ^{1}H NMR. Conditions: indene (300 µmol, 1.00 eq.), XBr (600 µmol, 2.00 eq.), $PhI(OAc)_2$ (450 µmol, 1.50 eq.), $TMSN_3$ (1.05 mmol, 3.50 eq.) in dry DCE (6.00 mL) under argon atm. Dod = *n*-dodecyl.

2.4. Optimization studies on the selective oxidation of secondary alcohols using $PhI(OAc)_2$, $TMSN_3$ and Et_4PBr without LED light irradiation

Table S4: Optimization studies on the selective oxidation of secondary alcohols using $PhI(OAc)_2$, $TMSN_3$ and Et_4PBr without LED light irradiation.

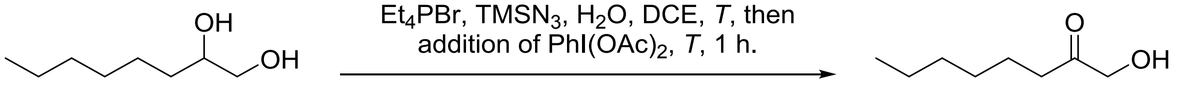

Entry	$TMSN_3$ /eq.	H_2O /eq.	$PhI(OAc)_2$ /eq.	Et_4PBr /eq.	T / °C	Additive	Solv.	Yield /%
1	6.0	6.0	3.5	3.0	25	-	DCE	44
2	8.0	8.0	6.0	4.0	25	-	DCE	35
3	8.0	8.0	3.0	2.0	25	-	DCE	48
4	10.0	10.0	3.0	2.0	25	-	DCE	48
5	12.0	12.0	3.0	2.0	25	-	DCE	35
6	8.0	-	3.0	2.0	25	-	DCE	traces
7	8.0	2.0	3.0	2.0	25	-	DCE	12
8	8.0	4.0	3.0	2.0	25	-	DCE	44
9	8.0	4.0	3.0	1.5	25	-	DCE	32
10	8.0	40.0	3.0	2.0	25	-	DCE	51
11	8.0	40.0	3.0	2.0	0	-	DCE	46
12	8.0	40.0	3.0	2.0	60	-	DCE	18
13	4.0 + 4.0 (after 30 min)	40.0	3.0 + 3.0 (after 30 min)	2.0 + 2.0 (after 30 min)	25	-	DCE	39
14	8.0	40.0	3.0	2.0	25	-	DME	18
15	8.0	40.0	3.0	2.0	25	-	MeCN	39
16	8.0	40.0	3.0	2.0	25	$Cu(OTf)_2$ (10 mol%)	DCE	51
17	8.0	40.0	3.0	2.0	25	$Zn(OTf)_2$ (10 mol%)	DCE	46
18	-	-	3.0	2.0	25	Bu_4NN_3 (8.0 eq.)	DCE	14
19	8.0	8.0	3.0	2.0	25	Et_3N (8.0 eq.)	DCE	traces
20	8.0	40.0	3.0	2.0	25	0.1 M pH7 buffer instead of water	DCE	44
21	8.0	40.0	-	2.0	25	$PhI(CF_3CO_2)_2$ (3.0 eq.)	DCE	44
22	8.0	40.0	-	2.0	25	PhI(OTs)(OH) (3.0 eq.)	DCE	55
23	8.0	8.0	-	2.0	25	Ph=O (3.0 eq.)	DCE	35

Conditions: octane-1,2-diol (300 µmol, 1.00 eq.), Et_4NBr, $TMSN_3$, H_2O in dry DCE (3.75 mL) under argon atm, then portionwise addition of $PhI(OAc)_2$.

2.5. Optimization studies on the selective oxidation of secondary alcohols using 1-azido-1,2-benziodoxol-3(1*H*)-one (Zhdankin's reagent) and Et_4PBr

Table S5: Optimization studies on the selective oxidation of secondary alcohols using 1-azido-1,2-benziodoxol-3(1*H*)-one (Zhdankin's reagent) and Et_4PBr.

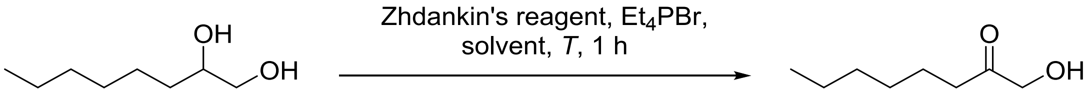

Entry	Zhdankin's reagent /eq.	Et_4PBr /eq.	Solv.	*T* / °C	Additive	Yield / %
1	1.5	1.0	DCE	25	-	39
2	3.0	2.0	DCE	25	-	54
3	4.5	3.0	DCE	25	-	64
4	6.0	4.0	DCE	25	-	65
5	3.0	3.0	DCE	25	-	38
6	4.5	2.0	DCE	25	-	52
7	4.5	4.5	DCE	25	-	45
8	4.5	3.0	DCE	0	-	61
9	4.5	3.0	DCE	–10	-	59
10	6.0	4.0	DCE	0 → 25	-	54
11	4.5	3.0	DCE	25 → 50	-	65
12	6.0	4.0	DCE	0 → 50	-	61
13	6.0	4.0	$PhCF_3$	25	-	40
14[1]	6.0	-	DCE	25	NBS (4.0 eq.)	41
15	4.5	3.0	DCE	25 → 50	CuCl (5 mol%)	60
16	6.0	4.0	DCE	–25	Blue LED light	69
17[2]	6.0	4.0	DCE	–25	Blue LED light	65

[1]24 h instead of 1 h reaction time; [2]A 1:1 mixture of starting material and product was used (0.15 mmol each); the yield is based on the maximum amount of product that can be formed with this mixture (0.30 mmol). Conditions: octane-1,2-diol (300 µmol, 1.00 eq.), Zhdankin's reagent, Et_4PBr in dry DCE (6.00 mL) under argon atm.

3. Chemical syntheses

3.1. Selective 1,2-bromoazidation of olefins

Method A: Selective 1,2-bromoazidation of olefins using $PhI(OAc)_2$, $TMSN_3$ and Et_4PBr under blue LED light irradiation

A suspension of $PhI(OAc)_2$ (145 mg, 450 µmol, 1.50 eq.) in dry DCE (6.00 mL) was cooled to −25 °C under argon. Et_4PBr (136 mg, 600 µmol, 2.00 eq.) was added and stirring continued for 30 min at −25 °C. Then $TMSN_3$ (138 µL, 1.05 mmol, 3.50 eq.) was added and the mixture stirred for additional 30 min at −25 °C. The alkene (300 µmol, 1.00 eq.) was added and the mixture irradiated with a blue LED. The reaction was monitored by thin layer chromatography and terminated by addition of $Na_2S_2O_3$ solution (aq., sat.). The aqueous phase was separated and washed with CH_2Cl_2 (2 x). The combined organic layers were dried over Na_2SO_4 and dried in vacuo to give the crude product, which was purified by flash column chromatography.

Method B: Selective 1,2-bromoazidation of olefins using 1-azido-1,2-benziodoxol-3(1*H*)-one (Zhdankin's reagent) and Et_4PBr under blue LED light irradiation

To a suspension of Zhdankin's reagent (520 mg, 1.80 mmol, 6.00 eq.) in DCE (5.00 mL) was added Et_4PBr (273 mg, 1.20 mmol, 4.00 eq.) at −25 °C and the resulting mixture stirred for 20 min. A solution of the alkene (300 µmol, 1.00 eq.) in DCE (1.00 mL) was then added to the orange suspension and stirred at −25 °C for 2 h. The reaction was stopped by addition of $Na_2S_2O_3$ solution (aq., sat.) and the separated aqueous phase extracted with CH_2Cl_2 (2 x). The combined organic phases were subsequently washed with $NaHCO_3$ solution (aq., sat.) and after separation of the layers the aqueous phase extracted with CH_2Cl_2 (2 x). The organic phases were combined and after purification by column chromatography, the product isolated.

Method C: Selective 1,2-bromoazidation of olefins using 1-azido-1,2-benziodoxol-3(1*H*)-one (Zhdankin's reagent) and Et_4PBr without LED light irradiation

To a mixture of Et_4PBr (273 mg, 1.20 mmol, 4.00 eq.) and alkene (300 µmol, 1.00 eq.) in DCE (5.00 mL) was portionwise added Zhdankin's reagent (520 mg, 1.80 mmol, 6.00 eq.) over 30 min at room temperature while clearly observing gas formation and a color change from yellow to orange. The suspension was then stirred for further 1.5 h at room temperature, the reaction stopped by addition of $Na_2S_2O_3$ solution (aq., sat.) and the separated aqueous phase extracted with CH_2Cl_2 (2 x). The combined organic phases were subsequently washed with $NaHCO_3$ solution (aq., sat.) and after separation of the layers the aqueous phase extracted with CH_2Cl_2 (2 x). The organic phases were combined and after purification by column chromatography, the product isolated.

2-Azido-1-bromo-2,3-dihydro-1*H*-indene (11a)

The title compound was prepared according to **Method A** using indene (34.8 mg, 300 µmol) in 93% yield (66.7 mg, 280 µmol, *trans*/*cis* 10:1).

The title compound was also prepared according to **Method B** using indene (34.8 mg, 300 µmol) in 97% yield (69.1 mg, 290 µmol, *trans*/*cis* 11:1).

Column chromatography Petroleum ether.

Major product - (1*R,2*R**)-2-azido-1-bromo-2,3-dihydro-1*H*-indene (*trans*-11a):**

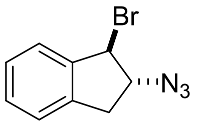

***trans*-11a**

Appearance colorless oil; **R_f** = 0.34 (petroleum ether); **^{1}H NMR** (400 MHz, $CDCl_3$) δ [ppm] = 7.47 – 7.40 (m, 1H), 7.34 – 7.29 (m, 2H), 7.28 - 7.27 (m, 1H), 5.33 (d, J = 3.2 Hz, 1H), 4.52 (dt, J = 6.7, 3.5 Hz, 1H), 3.45 (dd, *J* = 16.4, 6.4 Hz, 1H), 2.97 (dd, *J* = 16.4, 3.7 Hz, 1H); **^{13}C{^{1}H} NMR** (101 MHz, $CDCl_3$) δ [ppm] 140.6, 139.9, 129.8, 128.2, 125.9, 125.1, 70.0, 54.7, 36.7; **IR** ν_{max} [cm^{-1}] 2097.

The analytical data are in accordance with those reported in the literature.[S10]

Minor product - (1*R,2*S**)-2-azido-1-bromo-2,3-dihydro-1*H*-indene (*cis*-11a):**

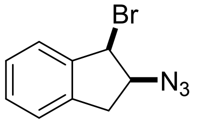

***cis*-11a**

Appearance colorless oil; **R_f** = 0.10 (petroleum ether); **^{1}H NMR** (400 MHz, $CDCl_3$) δ [ppm] = 7.42 – 7.40 (m, 1H), 7.33 – 7.24 (m, 3H), 5.52 (d, *J* = 5.3 Hz, 1H), 4.14 (dt, *J* = 7.7, 5.3 Hz, 1H), 3.16 – 3.10 (m, 2H); **^{13}C{^{1}H} NMR** (101 MHz, $CDCl_3$) δ [ppm] 141.0, 139.2, 130.0, 128.2, 125.4, 125.3, 63.6, 57.3, 35.2; **IR** ν_{max} [cm^{-1}] 2099.

The analytical data are in accordance with those reported in the literature.[S10]

1-(2-Azido-1-bromoethyl)-4-(*tert*-butyl)benzene (11b)

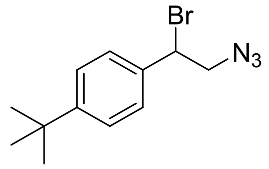

11b

The title compound was prepared according to **Method A** using 1-(*tert*-butyl)-4-vinylbenzene (48.1 mg, 300 μmol) in 89% yield (75.0 mg, 266 μmol).

The title compound was prepared according to **Method B** using 1-(*tert*-butyl)-4-vinylbenzene (48.1 mg, 300 μmol) in 97% yield (81.9 mg, 290 μmol).

Column chromatography Petroleum ether/EtOAc = 50:1; **Appearance** colorless oil; **R_f** = 0.37 (petroleum ether/EtOAc = 50:1); **^{1}H NMR** (400 MHz, $CDCl_3$) δ [ppm] 7.43 – 7.38 (m, 2H), 7.37 – 7.33 (m, 2H), 5.02 (t, *J* = 7.3 Hz, 1H), 3.95 – 3.80 (m, 2H), 1.32 (s, 9H); **^{13}C{^{1}H} NMR** (101 MHz, $CDCl_3$) δ [ppm] 152.5, 135.6, 127.4, 126.1, 57.7, 51.4, 34.8, 31.3; **IR** ν_{max} [cm^{-1}] 2100 (N_3); **HRMS** (EI) *m/z:* $[M]^+$ Calcd. for $C_{12}H_{16}N_3Br$ 281.0528; Found 281.0514.

(4-Azido-3-bromobutyl)benzene (11c)

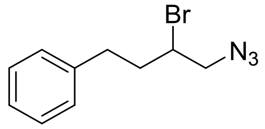

11c

The title compound was prepared according to **Method A** using but-3-en-1-ylbenzene (39.7 mg, 300 μmol) in 75% yield (56.9 mg, 224 μmol).

The title compound was prepared according to **Method B** using but-3-en-1-ylbenzene (39.7 mg, 300 μmol) in 82% yield (62.5 mg, 264 μmol).

Column chromatography Petroleum ether/EtOAc = 60:1; **Appearance** colorless oil; **R_f** = 0.27 (petroleum ether/EtOAc = 60:1); **^{1}H NMR** (400 MHz, $CDCl_3$) δ [ppm] 7.37 – 7.28 (m, 2H), 7.25 – 7.19 (m, 3H), 3.98 (dtd, J = 9.0, 6.0, 4.3 Hz, 1H), 3.64 (qd, J = 13.0, 6.0 Hz, 2H), 2.92 (ddd, J = 13.7, 8.3, 5.4 Hz, 1H), 2.76 (dt, J = 13.8, 8.0 Hz, 1H), 2.25 – 2.05 (m, 2H); **^{13}C{^{1}H} NMR** (101 MHz, $CDCl_3$) δ [ppm] 140.3, 128.7, 128.6, 126.5, 57.7, 52.5, 37.6, 33.4; **IR** ν_{max} [cm^{-1}] 2099 (N_3); **HRMS** (CI) *m/z:* $[M + H]^+$ Calcd. for $C_{10}H_{13}N_3Br$ 254.0293; Found 254.0280.

1-Azido-2-bromooctane (11d)

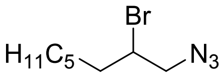

11d

The title compound could not be prepared according to **Method A**.

The title compound was prepared according to **Method B** using oct-1-ene (33.7 mg, 300 µmol) in 48% yield (33.4 mg, 143 µmol).

Column chromatography Petroleum ether; **Appearance** colorless oil; $\mathbf{R}_f$ = 0.54 (petroleum ether); **^{1}H NMR** (400 MHz, $CDCl_3$) δ [ppm] 4.07 – 4.01 (m, 1H), 3.62 (qd, J = 13.0, 6.1 Hz, 2H), 1.94 – 1.73 (m, 2H), 1.61 – 1.46 (m, 1H), 1.47 – 1.37 (m, 1H), 1.38 – 1.21 (m, 6H), 0.89 (t, J = 5.4 Hz, 3H); **^{13}C{^{1}H} NMR** (101 MHz, $CDCl_3$) δ [ppm] 57.6, 53.5, 36.1, 31.7, 28.7, 27.3, 22.7, 14.2; **IR** ν_{max} [cm^{-1}] 2099 (N_3); **HRMS** (CI) *m/z:* $[M + H]^+$ Calcd. for $C_8H_{17}N_3Br$ 234.0606; Found 234.0597.

2-Azido-3-bromobicyclo[2.2.1]heptane (11e)

The title compound was prepared according to **Method A** using 2-norbornene (28.2 mg, 300 µmol) in 47% yield (30.6 mg, 142 µmol, *cis*/*trans* 2:1).

The title compound was prepared according to **Method B** using 2-norbornene (28.2 mg, 300 µmol) in 53% yield (33.2 mg, 154 µmol, *cis*/*trans* 1.4:1).

Column chromatography Petroleum ether.

Major product - (1*S,2*R**,3*S**,4*R**)-2-Azido-3-bromobicyclo[2.2.1]heptane (*cis*-11e)**

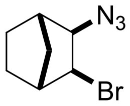

***cis*-11e**

Appearance colorless oil; $\mathbf{R}_f$ = 0.31 (petroleum ether); **^{1}H NMR** (400 MHz, $CDCl_3$) δ [ppm] 4.14 (dd, J = 6.6, 2.0 Hz, 1H), 3.55 (dd, J = 6.6, 1.0 Hz, 1H), 2.55 (d, J = 2.9 Hz, 1H), 2.27 (d, J = 2.7 Hz, 1H), 1.94 (dp, J = 10.6, 1.9 Hz, 1H), 1.69 - 1.56 (m, 2H), 1.28 - 1.20 (m, 3H); **^{13}C{^{1}H} NMR** (101 MHz, $CDCl_3$) δ [ppm] 67.2, 58.6, 47.1, 43.1, 33.6, 27.7, 26.0; **IR** ν_{max} [cm^{-1}] 2095 (N_3); **HRMS** (EI) *m/z:* $[M]^+$ Calcd. for $C_7H_{10}BrN_3$ 215.0056; Found 215.0058.

Minor product - (1*S,2*R**,3*R**,4*R**)-2-Azido-3-bromobicyclo[2.2.1]heptane (*trans*-11e)**

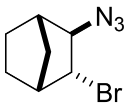

***trans*-11e**

Appearance colorless oil; $\mathbf{R}_f$ = 0.58 (petroleum ether); **^{1}H NMR** (400 MHz, $CDCl_3$) δ [ppm] 3.95 (dddd, J = 4.1, 3.1, 2.1, 1.0 Hz, 1H), 3.54 (t, J = 2.7 Hz, 1H), 2.45 (t, J = 3.8 Hz, 1H), 2.27 (d, J = 4.8 Hz, 1H), 1.90 (dddd, J = 12.8, 9.2, 4.3, 2.5 Hz, 1H), 1.69 - 1.66 (m, 2H), 1.56 – 1.47 (m, 1H), 1.42 (ddd, J = 10.7, 3.9, 1.7 Hz, 1H), 1.30 - 1.26 (m, 1H); **^{13}C{^{1}H} NMR** (101 MHz, $CDCl_3$) δ [ppm] 73.8, 57.9, 43.7, 42.5, 35.1, 26.2, 23.6; **IR** ν_{max} [cm^{-1}] 2093 (N_3); **MS** (EI) *m/z:* $[M – HNBr]^+$ Calcd. for $C_7H_9N_2$ 121.0; Found 121.0.

Relative stereochemistry was determined by comparing the *J-J* coupling values with those of the Cl-N_3 (*cis: J* = 6.8 Hz; *trans*: *J* = 4.2 Hz) analogues.[S11]

(1*R*,2*S*,5*S*)-2-(Azidomethyl)-2-bromo-6,6-dimethylbicyclo[3.1.1]heptane (11f)

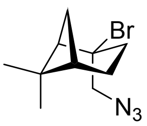

11f

The title compound was prepared according to **Method A** using (–)-*β*-pinene (40.9 mg, 300 µmol) in 25% yield (19.0 mg, 73.6 µmol).

The title compound was prepared according to **Method B** using (–)-*β*-pinene (40.9 mg, 300 µmol) in 33% yield (27.2 mg, 106 µmol).

Column chromatography Petroleum ether; **Appearance** colorless oil; $\mathbf{R}_f$ = 0.27 (petroleum ether); **$[\alpha]_D^{23}$** = –18.32° (c 0.16, $CHCl_3$); **^{1}H NMR** (400 MHz, $CDCl_3$) δ [ppm] 4.62 (ddd, J = 10.8, 4.5, 2.8 Hz, 1H), 3.44 (d, J = 13.0 Hz, 1H), 3.33 (d, J = 13.0 Hz, 1H), 2.59 (dddd, J = 14.0, 10.8, 4.5, 3.3 Hz, 1H),

2.07 – 1.96 (m, 1H), 1.79 (tq, J = 12.2, 4.0 Hz, 1H), 1.65 (t, J = 4.5 Hz, 1H), 1.60 – 1.57 (m, 1H), 1.53 – 1.47 (m, 1H), 1.33 (ddd, J = 12.3, 9.5, 4.3 Hz, 1H), 1.05 (s, 3H), 1.02 (s, 3H); **^{13}C{^{1}H} NMR** (101 MHz, $CDCl_3$) δ [ppm] 56.9, 53.8, 51.2, 47.3, 46.4, 40.6, 27.82, 27.80, 21.2, 19.9; **IR** ν_{max} [cm^{-1}] 2095 (N_3); **HRMS** (CI) *m/z:* $[M + H]^+$ Calcd. for $C_{10}H_{17}N_3Br$ 258.0598; Found 258.0606.

Ethyl (2*S,3*S**)-2-azido-3-bromo-3-(thiazol-4-yl)propanoate (11g)**

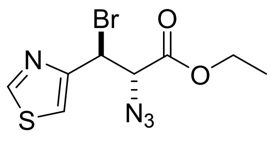

11g

The title compound was prepared according to **Method A** using Ethyl (*E*)-3-(thiazol-4-yl)acrylate (50.2 mg, 300 µmol) in 31% yield (28.7 mg, 94.1 µmol).

The title compound was prepared according to **Method B** using Ethyl (*E*)-3-(thiazol-4-yl)acrylate (50.2 mg, 300 µmol) in 33% yield (29.8 mg, 97.7 µmol).

Column chromatography Petroleum ether/EtOAc = 5:1; **Appearance** yellow oil; **R$_f$** = 0.30 (petroleum ether/EtOAc = 5:1); **^{1}H NMR** (400 MHz, $CDCl_3$) δ [ppm] 8.86 (d, J = 2.0 Hz, 1H), 7.51 (d, J = 2.0 Hz, 1H), 5.42 (d, J = 9.1 Hz, 1H), 4.77 (d, J = 9.1 Hz, 1H), 4.32 (qd, J = 7.1, 3.9 Hz, 2H), 1.33 (t, J = 7.1 Hz, 3H); **^{13}C{^{1}H} NMR** (101 MHz, $CDCl_3$) δ [ppm] 167.5, 154.1, 152.3, 118.9, 66.3, 62.6, 43.6, 14.2; **IR** ν_{max} [cm^{-1}] 2104 (N_3); **HRMS** (CI) *m/z:* $[M + H]^+$ Calcd. for $C_8H_{10}N_4O_2SBr$ 304.9708; Found 304.9716.

(2*S*,3*R)-Methyl 2-azido-3-bromo-3-phenylpropanoate (11h)**

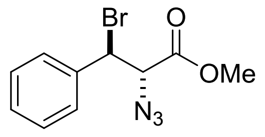

11h

The title compound could not be prepared according to **Method A**.

The title compound was prepared according to **Method B** using methyl cinnamate (48.7 mg, 300 µmol) in 13% yield (11.4 mg, 40.1 µmol).

Column chromatography Petroleum ether/EtOAc = 4:1; **Appearance** colorless oil; **R$_f$** = 0.32 (petroleum ether/EtOAc = 4:1; **^{1}H NMR** (400 MHz, $CDCl_3$): δ [ppm] 7.50 – 7.43 (m, 2H), 7.43 – 7.35 (m, 3H), 5.20 (d, J = 9.6 Hz, 1H), 4.42 (d, J = 9.6 Hz, 1H), 3.86 (s, 3H); **^{13}C{^{1}H} NMR** (101 MHz, $CDCl_3$): δ [ppm] 168.3, 136.8, 129.7, 129.2, 128.4, 67.4, 53.2, 49.5; **IR** ν_{max} [cm^{-1}] 2106 (N_3); **HRMS** (CI) *m/z:* $[M + H]^+$ Calcd. for $C_{10}H_{11}N_3O_2Br$ 284.0035; Found 284.0026.

Relative stereochemistry was determined by comparing the *J-J* coupling values with those of the I-N_3 (*trans:* J = 10 Hz) and OAc-N_3 (*trans:* J = 8 Hz; *cis*: J = 4 Hz) analogues.[S12]

(3*S*,5*R*,6*R*,8*S*,9*S*,10*R*,13*S*,14*S*)-6-Azido-5-bromo-10,13-dimethyl-17-oxohexadecahydro-1*H*-cyclopenta[*a*]phenanthren-3-yl acetate (11i)

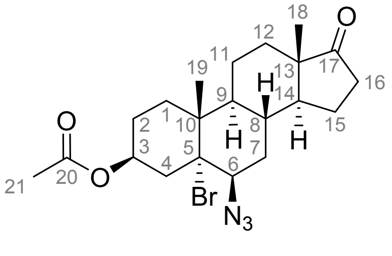

11i

The title compound was prepared according to **Method A** using prasterone acetate (99.1 mg, 300 µmol) in 51% yield (68.8 mg, 152 µmol).

The title compound was prepared according to **Method B** using prasterone acetate (99.1 mg, 300 µmol) in 76% yield (103 mg, 227 µmol).

Column chromatography Petroleum ether/EtOAc = 4:1; **Appearance** white needles; $\mathbf{R}_f$ = 0.34 (petroleum ether/EtOAc = 4:1); **mp.** = 169 °C (decomposition); $[\alpha]_D^{22.3}$ = 6.30 (c 0.64, $CHCl_3$); **^{1}H NMR** (400 MHz, $CDCl_3$) 5.45 (tt, *J* = 10.7, 5.5 Hz, 1H, *H*-3), 4.09 (dd, *J* = 4.1, 2.2 Hz, 1H, *H*-6), 2.53 – 2.41 (m, 2H, *H*-4 / *H*-16), 2.40 – 2.33 (m, 1H, *H*-7), 2.25 (ddd, *J* = 13.7, 5.5, 1.6 Hz, 1H, *H*-4'), 2.17 – 2.07 (m, 1H, *H*-16'), 2.05 (s, 3H, 3 x *H*-21), 1.95 (dddd, *J* = 19.4, 14.4, 5.1, 2.9 Hz, 3H, *H*-2 / *H*-15 / *H*-7'), 1.83 (ddd, *J* = 11.7, 9.5, 5.1 Hz, 2H, *H*-8, *H*-12), 1.77 – 1.70 (m, 1H, *H*-1), 1.67 – 1.54 (m, 5H, *H*-1' / *H*-11 / *H*-15' / *H*-2' / *H*-9), 1.51 – 1.39 (m, 1H, *H*-14), 1.38 – 1.18 (m, 5H, 3 x *H*-19 / *H*-11', *H*-12'), 0.88 (s, 3H, 3 x *H*-18); **^{13}C{^{1}H} NMR** (101 MHz, $CDCl_3$) *δ* [ppm] 220.2 (*C*-17), 170.4 (*C*-20), 84.8 (*C*-5), 71.5 (*C*-3), 67.1 (*C*-6), 50.6 (*C*-14), 47.8 (*C*-13), 47.5 (*C*-9), 41.0 (*C*-10), 39.2 (*C*-4), 35.8 (*C*-16), 35.2 (*C*-1), 31.3 (*C*-12), 30.9 (*C*-7), 30.7 (*C*-8), 26.3 (*C*-2), 21.7 (*C*-15), 21.4 (*C*-21), 20.6 (*C*-11), 17.6 (*C*-19), 14.0 (*C*-18); **IR** ν_{max} [cm^{-1}] 2097 (N_3); **HRMS** (EI) *m/z:* $[M]^+$ Calcd. for $C_{21}H_{30}N_3O_3Br$ 451.1471; Found 451.1472.

5-Azido-6-bromo-6-methylheptan-2-one (11ja)

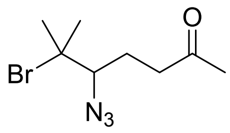

11ja

The title compound was prepared according to **Method A** using 6-methylhept-5-en-2-one (37.9 mg, 300 µmol) in 15% yield (11.2 mg, 45.1 µmol).

The title compound was prepared according to **Method B** using 6-methylhept-5-en-2-one (37.9 mg, 300 µmol) in 21% yield (15.8 mg, 63.7 µmol).

Column chromatography Petroleum ether/EtOAc = 100:1; **Appearance** colorless oil; $\mathbf{R}_f$ = 0.15 (petroleum ether/EtOAc = 9:1); **^{1}H NMR** (400 MHz, $CDCl_3$) *δ* [ppm] 3.35 (dd, *J* = 11.3, 2.4 Hz, 1H), 2.79 – 2.56 (m, 2H), 2.31 – 2.23 (m, 1H), 2.20 (s, 3H), 1.81 (s, 3H), 1.79 (s, 3H), 1.76 – 1.63 (m, 1H); **^{13}C{^{1}H} NMR** (101 MHz, $CDCl_3$) *δ* [ppm] 207.5, 72.4, 68.6, 40.5, 31.1, 30.9, 30.2, 25.1; **IR** ν_{max} [cm^{-1}] 2095 (N_3); **MS** (EI) *m/z:* $[M – N_2]^+$ Calcd. for $C_8H_{14}BrNO$ 219.0; Found 219.0.

6-Azido-5-bromo-6-methylheptan-2-one (11jb)

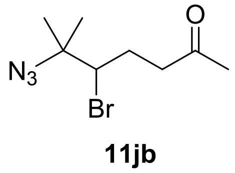

11jb

The title compound was prepared according to **Method A** using 6-methylhept-5-en-2-one (37.9 mg, 300 µmol) in 30% yield (22.4 mg, 90.3 µmol,).

The title compound was prepared according to **Method B** using 6-methylhept-5-en-2-one (37.9 mg, 300 µmol) in 36% yield (27.1 mg, 109 µmol).

Column chromatography Petroleum ether/EtOAc = 100:1; **Appearance** colorless oil; $\mathbf{R}_f$ = 0.18 (petroleum ether/EtOAc = 9:1); **^{1}H NMR** (400 MHz, $CDCl_3$) *δ* [ppm] 3.92 (dd, *J* = 11.6, 2.1 Hz, 1H), 2.91 – 2.61 (m, 2H), 2.36 (dtd, *J* = 15.0, 7.5, 2.1 Hz, 1H), 2.20 (s, 3H), 1.95 – 1.84 (m, 1H), 1.50 (s, 3H), 1.47 (s, 3H); **^{13}C{^{1}H} NMR** (101 MHz, $CDCl_3$) *δ* [ppm] 207.6, 64.3, 64.2, 42.1, 30.3, 27.7, 24.9, 23.8; **IR** ν_{max} [cm^{-1}] 2099 (N_3); **HRMS** (CI) *m/z:* $[M + H]^+$ Calcd. for $C_{10}H_{17}N_3Br$ 248.0398; Found 248.0402.

(3,4-Dibromobutyl)benzene (12)

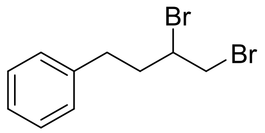

12

The title compound was prepared according to **Method C** using but-3-en-1-ylbenzene (39.7 mg, 300 µmol) in 55% yield (48.1 mg, 165 µmol).

Column chromatography Petroleum ether/EtOAc = 60:1; **Appearance** colorless oil; $\mathbf{R}_f$ = 0.45 (petroleum ether/EtOAc = 60:1); ^{1}**H NMR** (400 MHz, $CDCl_3$) δ [ppm] 7.36 – 7.27 (m, 2H), 7.23 (d, *J* = 7.3 Hz, 3H), 4.12 (tdd, *J* = 9.6, 4.4, 2.9 Hz, 1H), 3.86 (dd, *J* = 10.3, 4.4 Hz, 1H), 3.65 (t, *J* = 10.0 Hz, 1H), 2.94 (ddd, *J* = 13.9, 9.3, 4.7 Hz, 1H), 2.76 (ddd, *J* = 13.7, 9.1, 7.2 Hz, 1H), 2.48 (dddd, *J* = 14.7, 9.3, 7.2, 2.9 Hz, 1H), 2.09 (dtd, *J* = 14.3, 9.3, 4.7 Hz, 1H); ^{13}**C{^{1}H} NMR** (101 MHz, $CDCl_3$) δ [ppm] 140.4, 128.72, 128.68, 126.4, 52.2, 37.8, 36.4, 33.1.

The analytical data are in accordance with those reported in the literature.[S13]

1,2-Dibromooctane (13)

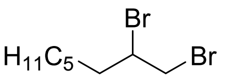

13

The title compound was prepared according to **Method C** using oct-1-ene (33.7 mg, 300 µmol) in 52% yield (42.6 mg, 157 µmol).

Column chromatography Petroleum ether; **Appearance** colorless oil; $\mathbf{R}_f$ = 0.71 (petroleum ether); ^{1}**H NMR** (400 MHz, $CDCl_3$) δ [ppm] 4.22 – 4.09 (m, 1H), 3.85 (dd, *J* = 10.2, 4.4 Hz, 1H), 3.63 (t, *J* = 10.0 Hz, 1H), 2.20 – 2.07 (m, 1H), 1.92 – 1.67 (m, 1H), 1.61 – 1.49 (m, 1H), 1.42 (ddd, *J* = 13.4, 6.8, 4.4 Hz, 1H), 1.38 – 1.24 (m, 6H), 0.93 – 0.86 (m, 3H); ^{13}**C{^{1}H} NMR** (101 MHz, $CDCl_3$) δ [ppm] 53.3, 36.5, 36.2, 31.7, 28.6, 26.9, 22.7, 14.2.

The analytical data are in accordance with those reported in the literature.[S14]

3.2. Selective oxidation of secondary alcohols

Proceeding I: Selective oxidation of secondary alcohols using $PhI(OAc)_2$, $TMSN_3$ and Et_4PBr under blue LED light irradiation

A suspension of $PhI(OAc)_2$ (291 mg, 900 µmol, 3.00 eq.) in dry DCE (12.0 mL) was cooled to −25 °C under an argon atmosphere. Et_4PBr (239 mg, 1.05 mmol, 3.50 eq.) was added and stirring continued for 30 min at −25 °C. $TMSN_3$ (236 µL, 1.81 mmol, 6.00 eq.) was added, followed by water (32.5 µL, 1.81 mmol, 6.00 eq.), and the mixture stirred for additional 30 min at −25 °C. Then, the alcohol (300 µmol, 1.00 eq.) was added and the mixture irradiated with blue LED light and allowed to warm to 0 °C over a period of 1 h. Subsequently, the reaction was terminated by addition of $Na_2S_2O_3$ solution (aq., sat.). The phases were separated and the aqueous phase was extracted with CH_2Cl_2 (2 x). The combined organic layers were dried over Na_2SO_4, filtered and concentrated under reduced pressure to give the crude product, which was purified by flash column chromatography.

Proceeding II: Selective oxidation of secondary alcohols using $PhI(OAc)_2$, $TMSN_3$ and Et_4PBr without LED light irradiation

A solution of the alcohol (300 µmol, 1.00 eq.) in DCE (3.75 mL) was treated with $TMSN_3$ (314 µL, 2.40 mmol, 8.00 eq.), Et_4PBr (136 mg, 600 µmol, 2.00 eq.) and water (216 µL, 12.0 mmol, 40.0 eq.) at room temperature. Then $PhI(OAc)_2$ (290 mg, 900 µmol, 3.00 eq.) was added portionwise over 30 min. When the solid was added, nitrogen formation and a yellow coloration of the solution became apparent, which disappeared after a few minutes. After complete addition, the mixture was stirred for additional 30 min before the reaction was terminated by the addition of $Na_2S_2O_3$ solution (aq., sat.). The phases were separated and the aqueous phase was extracted with CH_2Cl_2 (2 x). The combined organic layers were dried over Na_2SO_4, filtered and concentrated under reduced pressure to give the crude product, which was purified by flash column chromatography.

Proceeding III: Selective oxidation of secondary alcohols using 1-azido-1,2-benziodoxol-3(1*H*)-one (Zhdankin's reagent) and Et_4PBr under blue LED light irradiation

A solution of the alcohol (300 µmol, 1.00 eq.) in DCE (6.00 mL) was treated with 1-azido-1,2-benziodoxol-3(1*H*)-one (522 mg, 1.81 mmol, 6.00 eq.) and Et_4PBr (273 mg, 1.20 mmol, 4.00 eq.) at −25 °C under an argon atmosphere. Then, the mixture irradiated with blue LED light and allowed to warm to 0 °C over a period of 1 h. Subsequently, the reaction was terminated by addition of $Na_2S_2O_3$ solution (aq., sat.) and K_2CO_3 solution (aq., 10 wt%). The phases were separated and the aqueous phase was extracted with CH_2Cl_2 (2 x). The combined organic layers were dried over Na_2SO_4, filtered and concentrated under reduced pressure to give the crude product, which was purified by flash column chromatography.

Proceeding IV: Selective oxidation of secondary alcohols using 1-azido-1,2-benziodoxol-3(1*H*)-one (Zhdankin's reagent) and Et_4PBr without LED light irradiation

A suspension of the alcohol (300 µmol, 1.00 eq.) and 1-azido-1,2-benziodoxol-3(1*H*)-one (390 mg, 1.35 mmol, 4.50 eq.) in DCE (3.75 mL) was treated with and Et_4PBr (204 mg, 900 µmol, 3.00 eq.) at room temperature under an argon atmosphere. The mixture was then stirred at 50 °C for 1 h, during which time the formation of nitrogen bubbles can be observed. Subsequently, the reaction was terminated by addition of $Na_2S_2O_3$ solution (aq., sat.) and K_2CO_3 solution (aq., 10 wt%). The phases were separated and the aqueous phase was extracted with CH_2Cl_2 (2 x). The combined organic layers

were dried over Na_2SO_4, filtered and concentrated under reduced pressure to give the crude product, which was purified by flash column chromatography.

Benzophenone (20a)

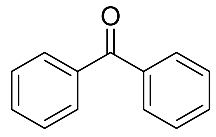

20a

The title compound was prepared according to **proceeding I** using 1-phenyl-1,2-ethanediol (41.4 mg, 300 µmol) in 99% yield (54.1 mg, 297 µmol).

The title compound was also prepared according to **proceeding III** using 1-phenyl-1,2-ethanediol (55.3 mg, 300 µmol) in quantitative yield (54.5 mg, 299 µmol).

Column chromatography Petroleum ether/EtOAc = 19:1; **Appearance** colorless solid; $\mathbf{R}_f$ = 0.29 (petroleum ether/EtOAc = 9:1); ^{1}**H NMR** (400 MHz, $CDCl_3$) δ [ppm] 7.81 (dd, J = 8.3, 1.4 Hz, 2H), 7.63 – 7.55 (m, 1H), 7.51 (tt, J = 6.5, 1.3 Hz, 2H); ^{13}**C{^{1}H} NMR** (101 MHz, $CDCl_3$) δ [ppm] 196.9, 137.7, 132.5, 130.2, 128.4.

The analytical data are in accordance with those reported in the literature.[S15]

(*1R*,4*S*)-1,3,3-Trimethylbicyclo[2.2.1]heptan-2-one (20b)

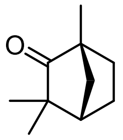

20b

The title compound was prepared according to **proceeding III** using (+)-fenchol (46.2 mg, 300 µmol) in 45% yield (20.3 mg, 133 µmol).

Column chromatography Petroleum ether/EtOAc = 30:1 → 9:1; **Appearance** colorless oil; $\mathbf{R}_f$ = 0.47 (toluene/EtOAc = 9:1); $[\alpha]_D^{27}$ = –41.5° (*c* 1.1, acetone), {Lit. $[\alpha]_D^{20}$ = –44° (*c* 1.0, hexane)[S16]}; ^{1}**H NMR** (400 MHz, $CDCl_3$) δ [ppm] 2.13 (br s, 1H), 1.82 – 1.67 (m, 3H), 1.59 – 1.51 (m, 2H), 1.42 – 1.34 (m, 1H), 1.14 (s, 3H), 1.03 (s, 6H); ^{13}**C{^{1}H} NMR** (101 MHz, $CDCl_3$) δ [ppm] 223.6, 54.3, 47.5, 45.5, 41.8, 32.0, 25.1, 23.5, 21.9, 14.8.

The analytical data are in accordance with those reported in the literature.[S17]

(2*S*,5*R*)-2-Isopropyl-5-methylcyclohexan-1-one (20c)

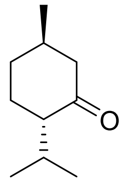

20c

The title compound was prepared according to **proceeding III** using (–)-menthol (47.0 mg, 300 µmol) in 45% yield (21.1 mg, 137 µmol).

Column chromatography Petroleum ether/EtOAc = 20:1; **Appearance** colorless oil; $\mathbf{R}_f$ = 0.54 (petroleum ether/EtOAc = 9:1); $[\alpha]_D^{27}$ = –28.4° (*c* 0.54, $CHCl_3$) {Lit. $[\alpha]_D^{20}$ = –23.1° (*c* 5.18, $CHCl_3$)[S18]}; ^{1}**H NMR** (400 MHz, $CDCl_3$) δ [ppm] 2.33 (ddd, J = 12.9, 3.9, 2.2 Hz, 1H), 2.16 – 1.79 (m, 6H) 1.43 – 1.22 (m, 2H), 0.99 (d, J = 6.2 Hz, 3H), 0.89 (d, J = 6.8 Hz, 3H), 0.83 (d, J = 6.8 Hz, 3H); ^{13}**C{^{1}H} NMR** (101 MHz, $CDCl_3$) δ [ppm] 212.6, 56.0, 51.0, 35.6, 34.1, 28.0, 26.0, 22.4, 21.3, 18.8.

The analytical data are in accordance with those reported in the literature.[S19]

1-Hydroxyoctan-2-one (22)

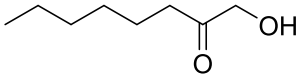

22

The title compound was prepared according to **proceeding I** using octane-1,2-diol (43.9 mg, 300 µmol) in 32% yield (13.8 mg, 95.7 µmol).

The title compound was also prepared according to **proceeding II** using octane-1,2-diol (43.9 mg, 300 µmol) in 51% yield (22.0 mg, 153 µmol).

The title compound was also prepared according to **proceeding III** using octane-1,2-diol (43.9 mg, 300 µmol) in 69% yield (29.8 mg, 207 µmol).

The title compound was also prepared according to **proceeding IV** using octane-1,2-diol (43.9 mg, 300 µmol) in 65% yield (28.1 mg, 195 µmol).

Column chromatography Petroleum ether/EtOAc = 3:1; **Appearance** colorless oil; $\mathbf{R}_f$ = 0.54 (petroleum ether/EtOAc = 1:1); ^{1}**H NMR** (400 MHz, $CDCl_3$) δ [ppm] 4.24 (s, 2H), 3.00 (br s, 1H), 2.40 (t, J = 7.5 Hz, 1H), 1.66 – 1.59 (m, 2H), 1.33 – 1.25 (m, 6H), 0.87 (t, J = 6.6 Hz, 3H); ^{13}**C{^{1}H} NMR** (101 MHz, $CDCl_3$) δ [ppm] 210.1, 68.2, 38.6, 31.6, 29.0, 23.8, 22.6, 14.1.

The analytical data are in accordance with those reported in the literature.[S20]

3-(Hydroxymethyl)heptan-4-one (24)

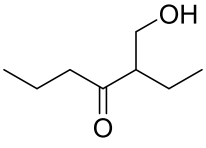

24

The title compound was prepared according to **proceeding III** using 2-ethylhexane-1,3-diol (43.9 mg, 300 µmol) in 58% yield (25.1 mg, 174 µmol).

The title compound was also prepared according to **proceeding IV** using 2-ethylhexane-1,3-diol (43.9 mg, 300 µmol) in 53% yield (23.1 mg, 160 µmol).

Column chromatography Petroleum ether/EtOAc = 3:1; **Appearance** colorless oil; $\mathbf{R}_f$ = 0.48 (petroleum ether/EtOAc = 1:1); ^{1}**H NMR** (400 MHz, $CDCl_3$) δ [ppm] 3.79 (dd, J = 11.0, 7.3 Hz, 1H), 3.69 (dd, J = 11.1, 4.0 Hz, 1H), 2.61 (qd, J = 11.5, 4.1 Hz, 1H), 2.47 (t, J = 7.2 Hz, 2H), 2.13 (br s, 1H), 1.71 – 1.47 (m, 4H), 0.91 (td, J = 11.1, 4.4 Hz, 6H); ^{13}**C{^{1}H} NMR** (101 MHz, $CDCl_3$) δ [ppm] 215.3, 62.6, 55.1, 44.9, 21.4, 17.0, 13.9, 12.0.

The analytical data are in accordance with those reported in the literature.[S15]

2-Hydroxy-1-phenylethan-1-one (26)

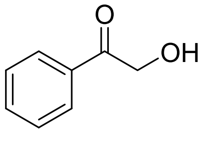

26

The title compound was prepared according to **proceeding I** using 1-phenyl-1,2-ethanediol (41.4 mg, 300 µmol) in 88% yield (36.1 mg, 265 µmol).

The title compound was also prepared according to **proceeding III** using 1-phenyl-1,2-ethanediol (41.4 mg, 300 µmol) in 96% yield (39.1 mg, 287 µmol).

The title compound was also prepared according to **proceeding IV** using 1-phenyl-1,2-ethanediol (41.4 mg, 300 µmol) in 72% yield (29.3 mg, 216 µmol).

Column chromatography Petroleum ether/EtOAc = 3:1; **Appearance** colorless solid; $\mathbf{R}_f$ = 0.48 (petroleum ether/EtOAc = 1:1); ^{1}**H NMR** (400 MHz, $CDCl_3$) δ [ppm] 7.93 (dd, J = 8.4, 1.4 Hz, 2H), 7.63 (ddt, J = 8.0, 6.9, 1.3 Hz, 1H), 7.51 (ddd, J = 8.0, 6.7, 1.2 Hz, 2H), 4.88 (s, 2H), 3.51 (br s, 1H); ^{13}**C{^{1}H} NMR** (101 MHz, $CDCl_3$) δ [ppm] 198.5, 134.5, 133.5, 129.1, 127.9, 65.6.

The analytical data are in accordance with those reported in the literature.[S21]

3.3. Mechanistic studies

Bromide-mediated release of azide radicals from Zhdankin's reagent (7)

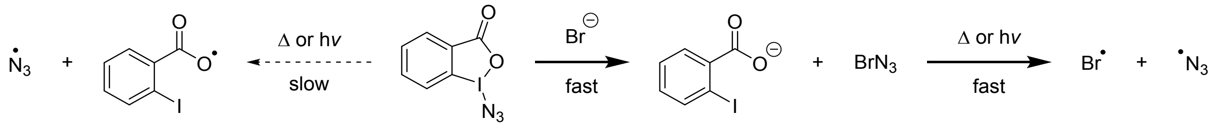

A solution of Et_4PBr, Zhdankin's reagent and TEMPO in DCE turns into an intense red-brown color within a few minutes at room temperature due to the formation of the TEMPO-N_3 charge-transfer complex. Without the addition of the bromide, a brown color becomes visible only after 12 h.

We assume that the Zhdankin's reagent (**7**) rapidly forms BrN_3 when added to a bromide. BrN_3 itself is very unstable and decomposes both thermally and photolytically to form both the Br and N_3 radicals.

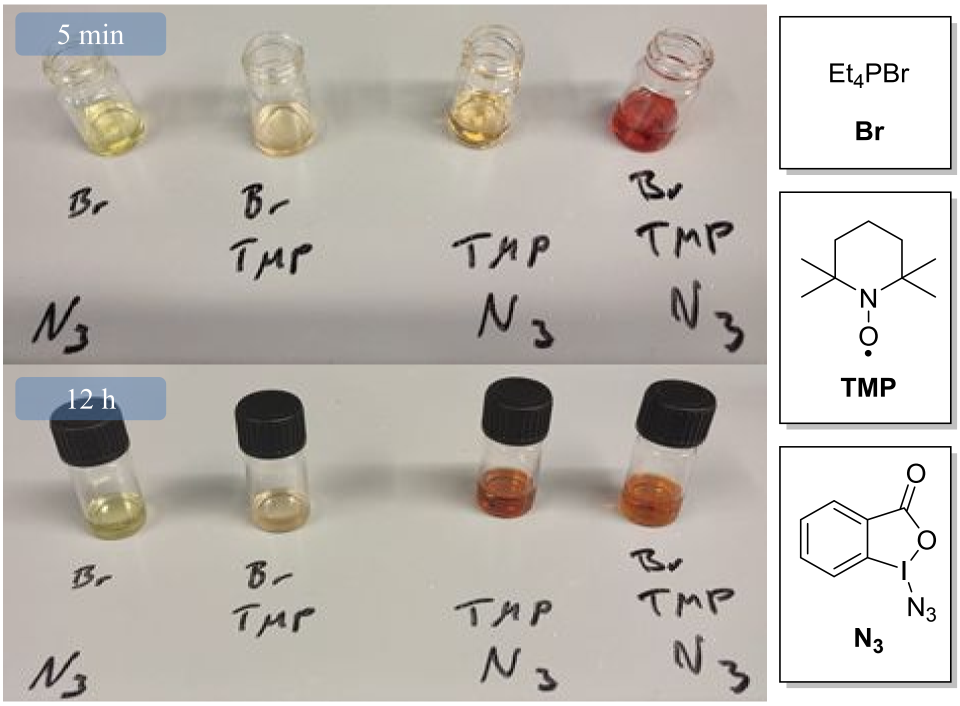

Figure S2: Experiment to demonstrate the accelerated release of azide radicals from Zhdankin's reagent when a bromide sourse is added.

Detection of bromine azide formation by UV-Vis Spectroscopy

To detect the formation of BrN_3 from the reaction of Zhdankin's reagent and Et_4PBr, UV-Vis spectra of the two reagents were recorded individually and as a mixture in CCl_4 (sat.) at room temperature (Figure S3). The solution of the mixture turned slightly yellowish indicating the formation of BrN_3. However, the UV-Vis spectrum of the mixture showed the same absorption maximum as that of the pure Zhdankin's reagent ($\lambda_{max} = 273$ nm), but with a broadening of the signal into the longer wavelength region.

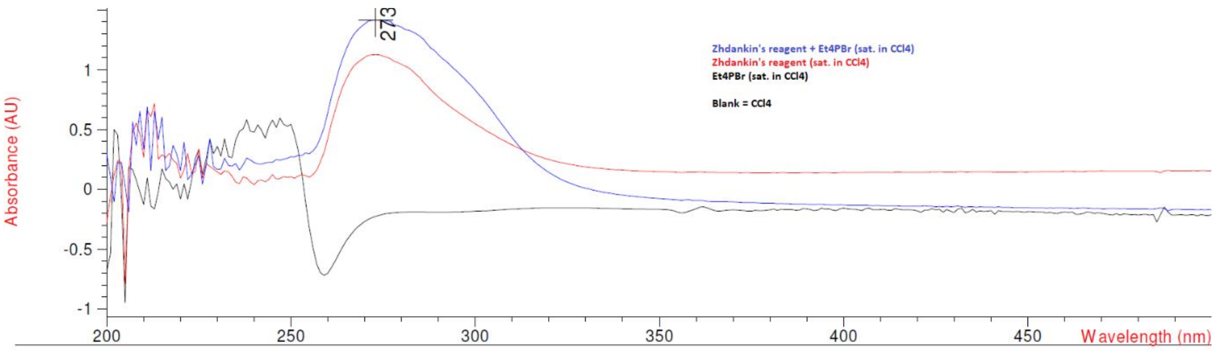

Figure S3: Overlaid UV-vis spectra of Et_4PBr, Zhdankin's reagent and a mixture of both in CCl_4.

It can therefore be assumed that the signal referring to bromine azide is covered by the signal associated by Zhdankin's reagent. For this reason, a difference spectrum was recorded from the Zhdankin's reagent and the mixture (solution of Zhdankin's reagent as blank, sat. solution of Zhdankin's reagent and Et_4PBr as sample, Figure S4). The detected absorption maximum $\lambda_{max} = 292$ nm is in accordance with the literature ($\lambda_{max} = 291$ nm) for BrN_3 in CCl_4.[S22] It has to be noted that the second absorption maximum at 420 nm, which is described in the literature as much less intense, could not be detected.

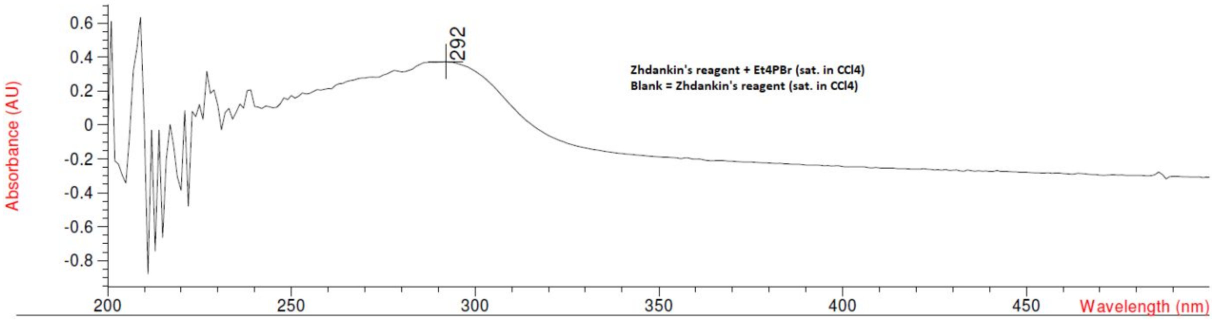

Figure S4: Difference spectrum of Zdankin's reagent and the mixture of Zdankin's reagent and Et_4PBr in CCl_4.

Azidooxygenation of indene (10a)

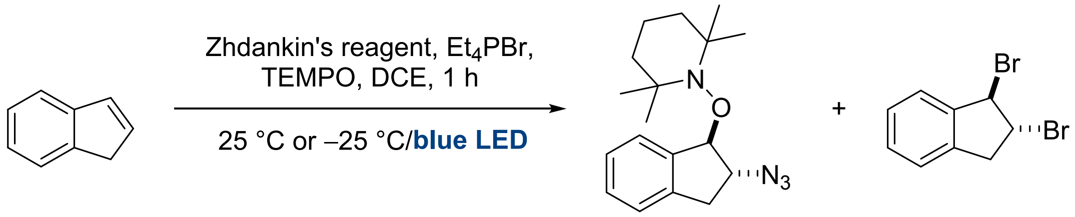

Scheme S1: Azidooxygenation of indene under photochemical and thermal conditions.

At room temperature

To a mixture of Et_4PBr (136 mg, 600 µmol, 2.00 eq.), alkene (300 µmol, 1.00 eq.) and TEMPO (93.7 mg, 600 µmol, 2.00 eq.) in DCE (5.00 mL) was portionwise added Zhdankin's reagent (260 mg, 900 µmol, 3.00 eq.) over 30 min at room temperature while clearly observing a color change from yellow to brown. The suspension was then stirred for further 1.5 h at room temperature, the reaction stopped by addition of $Na_2S_2O_3$ solution (aq., sat.) and the separated aqueous phase extracted with

CH_2Cl_2 (2 x). The combined organic phases were subsequently washed with $NaHCO_3$ solution (aq., sat.) and after separation of the layers the aqueous phase extracted with CH_2Cl_2 (2 x). The organic phases were combined and after purification by column chromatography (petroleum ether/diethyl ether = 40:1), TEMPO-adduct **14** (87%, 82.0 mg, 261 µmol) and dibromide **S1** (6%, 4 mg, 0.02 mmol) isolated.

At −25 °C and blue LED light irradiation

To a suspension of Zhdankin's reagent (260 mg, 900 µmol, 3.00 eq.) in DCE (5.00 mL) was added Et_4PBr (136 mg, 600 µmol, 2.00 eq.) at –25 °C and the resulting mixture stirred for 20 min. A solution of the alkene (300 µmol, 1.00 eq.) and TEMPO (93.7 mg, 600 µmol, 2.00 eq.) in DCE (1.00 mL) was then added to the orange suspension and stirred at –25 °C for 2 h. The reaction was stopped by addition of $Na_2S_2O_3$ solution (aq., sat.) and the separated aqueous phase extracted with CH_2Cl_2 (2 x). The combined organic phases were subsequently washed with $NaHCO_3$ solution (aq., sat.) and after separation of the layers the aqueous phase extracted with CH_2Cl_2 (2 x). The organic phases were combined and after purification by column chromatography (petroleum ether/diethyl ether = 40:1), TEMPO-adduct **14** (32%, 30.6 mg, 97.3 µmol) and dibromide **S1** (31%, 25.4 mg, 92.0 µmol) isolated.

1-(((1*R**,2*R**)-2-Azido-2,3-dihydro-1*H*-inden-1-yl)oxy)-2,2,6,6-tetramethylpiperidine (14)

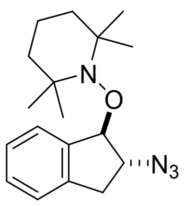

14

Appearance colorless oil; $\mathbf{R}_f$ = 0.45 (Petroleum ether/diethyl ether = 60:1); **^{1}H NMR** (400 MHz, $CDCl_3$) δ [ppm] 7.57 (d, *J* = 7.2 Hz, 1H, Ar*H*), 7.34 – 7.21 (m, 3H), 5.34 (d, *J* = 4.1 Hz, 1H), 4.41 (dt, *J* = 7.2, 4.7 Hz, 1H), 3.37 (dd, *J* = 16.2, 7.1 Hz), 2.88 (dd, *J* = 16.2, 5.2 Hz), 1.58 – 1.34 (m, 6H), 1.30 (s, 3H), 1.20 (s, 3H), 1.11 (s, 3H, CH_3), 1.05 (s, 3H, CH_3); **^{13}C{^{1}H} NMR** (101 MHz, $CDCl_3$) δ [ppm] 140.6, 140.2, 129.0, 126.9, 126.7, 124.8, 90.3, 66.8, 60.9, 60.0, 40.4, 36.8, 34.4, 33.9, 20.7, 17.4; **IR** ν_{max} [cm^{-1}] 2099 (N_3).

The analytical data are consistent with those reported in the literature.[S15]

(1*R**,2*R**)-1,2-Dibromo-2,3-dihydro-1*H*-indene (S1)

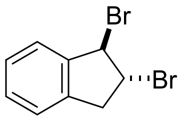

S1

Appearance colorless oil; $\mathbf{R}_f$ = 0.59 (petroleum ether/diethyl ether = 60:1); **^{1}H NMR** (400 MHz, $CDCl_3$) δ [ppm] 7.50 – 7.45 (m, 1H), 7.36 – 7.31 (m, 3H), 5.63 (s, 1H), 4.88 (dt, *J* = 5.2, 1.2 Hz, 1H), 3.82 (dd, *J* = 17.5, 5.3 Hz, 1H), 3.27 (d, *J* = 17.5 Hz, 1H); **^{13}C{^{1}H} NMR** (101 MHz, $CDCl_3$): δ [ppm] 140.7, 129.9, 128.1, 125.9, 125.6, 57.9, 54.6, 41.6.

The analytical data are consistent with those reported in the literature.[S23]

Radical cascade reaction: 3-(Azidomethyl)-4-(bromomethyl)-3-methyl-1-tosylpyrrolidine (16)

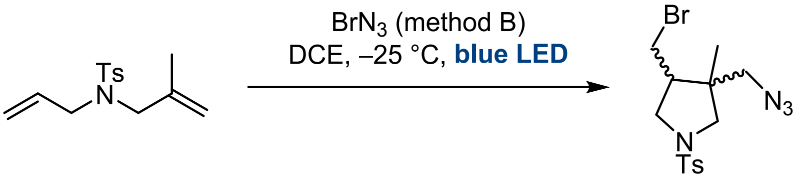

15 **16**

Scheme S2: Radical cascade reaction of *N*-allyl-4-methyl-*N*-(2-methylallyl)benzenesulfonamide (15) following method B for the 1,2-bromoazidation of olefins.

The title compound was prepared according to **Method B** using *N*-allyl-4-methyl-*N*-(2-methylallyl)benzenesulfonamide (79.6 mg, 300 µmol) as an inseparable mixture of diastereoisomers (*d.r.* 1.5:1) in 63% yield (74.1 mg, 191 µmol).

Column chromatography Petroleum ether/EtOAc = 4:1; **Appearance** colorless oil; $\mathbf{R}_f$ = 0.25 (petroleum ether/EtOAc = 4:1); **^{1}H NMR** (400 MHz, $CDCl_3$): δ [ppm] 7.72 (dd, J = 8.3, 6.7 Hz, 2H), 7.36 (ddt, J = 7.9, 2.4, 0.7 Hz, 2H), 3.67 (ddd, J = 16.9, 10.4, 7.9 Hz, 1H), 3.53 – 2.85 (m, 7H), 2.45 (d, J = 1.8 Hz, 3H), 2.40 – 2.24 (m, 1H), 1.07 & 0.89 (2 s, 3H total integral, CH_3); **^{13}C{^{1}H} NMR** (101 MHz, $CDCl_3$) δ [ppm] 144.0 (d), 133.5, 133.4, 130.0 (d), 127.7, 127.6, 58.5, 58.0, 57.4, 54.8, 52.2 (d), 50.0, 46.5, 45.5, 45.4, 30.3, 29.7, 22.1, 17.4; **IR** ν_{max} [cm^{-1}] 2099 (N_3); **HRMS** (ESI) *m/z:* $[M + Na]^+$ Calcd. for $C_{14}H_{19}N_4O_2SBrNa$ 409.0310; Found 409.0310.

Radical clock experiment: Dibenzyl (*E*)-2-(4-azidobut-2-en-1-yl)-2-bromomalonate (18)

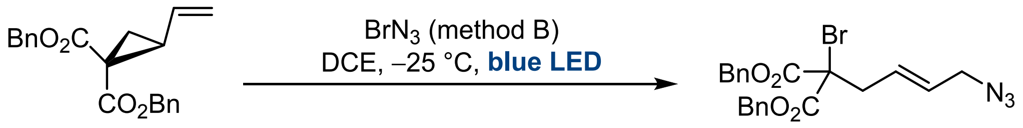

17 **18**

Scheme S3: Radical clock experiment of dibenzyl 2-vinylcyclopropane-1,1-dicarboxylate (17) following method B for the 1,2-bromoazidation of olefins.

The title compound was prepared according to **Method B** using dibenzyl 2-vinylcyclopropane-1,1-dicarboxylate (50.5 mg, 150 µmol) in 54% yield (37.3 mg, 81.4 µmol).

Column chromatography Petroleum ether/EtOAc = 50:1; **Appearance** colorless oil; $\mathbf{R}_f$ = 0.36 (petroleum ether/EtOAc = 9:1); **^{1}H NMR** (400 MHz, $CDCl_3$) δ [ppm] 7.35 – 7.27 (m, 10H), 5.72 – 5.61 (m, 1H), 5.59 – 5.47 (m, 1H), 5.25 – 5.13 (m, 4H), 3.60 (d, J = 5.3 Hz, 2H), 3.08 (d, J = 6.7 Hz, 2H); **^{13}C{^{1}H} NMR** (101 MHz, $CDCl_3$) δ [ppm] 166.2, 134.7, 129.3, 128.8, 128.7, 128.5, 128.3, 68.8, 61.4, 52.3, 41.2; **IR** ν_{max} [cm^{-1}] 2099 (N_3); **HRMS** (ESI) *m/z:* $[M + Na]^+$ Calcd. for $C_{21}H_{20}N_3O_4BrNa$ 480.0535; Found 480.0551.

3.4. Chemoselectivity studies

Chemoselectivity studies on bifunctionalized model substrate 27

Oct-7-en-2-ol (38.5 mg, 300 µmol) was applied to three different reactions according to **Method B**, varying the equivalents of Zhdankin's reagent (**7**) and Et_4PBr (**1**). The results of the experiments are listed in Table S6.

Table S6: Chemoselectivity studies on bifunctionalized model substrate 27.

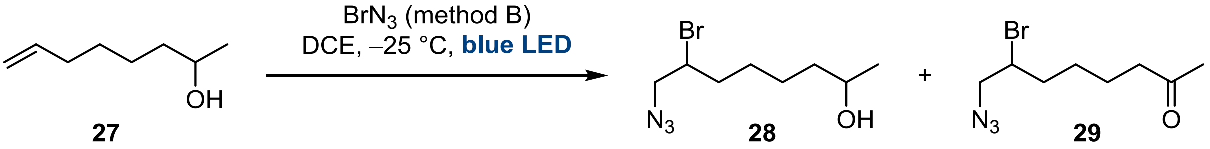

Entry	Zhdankin's reagent / eq.	Et_4PBr / eq.	27	28	29
1	6.0	4.0	-	-	83% (61.3 mg, 247 µmol)
2	3.0	2.0	-	-	81% (60.1 mg, 242 µmol)
3	1.5	1.0	53% (20.1 mg, 159 µmol)	28% (20.9 mg, 83.6 µmol)	-

8-Azido-7-bromooctan-2-ol (28)

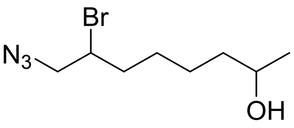

29

Column chromatography Petroleum ether/EtOAc = 2:1; **Appearance** colorless oil; **R*f*** = 0.15 (petroleum ether/EtOAc = 4:1); **^{1}H NMR** (400 MHz, $CDCl_3$) *δ* [ppm] 4.04 (dtd, *J* = 8.9, 6.1, 4.4 Hz, 1H), 3.79 (p, *J* = 6.1 Hz, 1H), 3.62 (qd, *J* = 13.0, 6.1 Hz, 2H), 1.96 – 1.75 (m, 2H), 1.65 – 1.53 (m, 1H), 1.51 – 1.45 (m, 2H), 1.45 – 1.39 (m, 2H), 1.35 (s, 1H), 1.19 (d, *J* = 6.2 Hz, 3H); **^{13}C{^{1}H} NMR** (101 MHz, $CDCl_3$) *δ* [ppm] 68.0, 57.6, 53.2, 39.1, 36.0, 27.3, 25.2, 23.7; **IR** ν_{max} [cm^{-1}] 3345 (OH), 2099 (N_3); **HRMS** (CI) *m/z:* $[M + H]^+$ Calcd. for $C_8H_{17}N_3OBr$ $[M+H]^+$ 250.0555; Found 250.0545.

8-Azido-7-bromooctan-2-one (29)

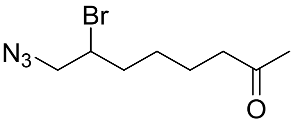

30

Column chromatography Petroleum ether/EtOAc = 10:1; **Appearance** yellow oil; **R*f*** = 0.27 (petroleum ether/EtOAc = 4:1); **^{1}H NMR** (400 MHz, $CDCl_3$) *δ* [ppm] 4.02 (dtd, *J* = 8.9, 6.0, 4.3 Hz, 1H), 3.61 (qd, *J* = 13.0, 6.0 Hz, 2H), 2.45 (t, *J* = 7.0 Hz, 2H), 2.13 (s, 3H), 2.02 – 1.72 (m, 1H), 1.68 – 1.48 (m, 3H), 1.45 – 1.34 (m, 1H); **^{13}C{^{1}H} NMR** (101 MHz, $CDCl_3$) *δ* [ppm] 208.6, 57.5, 53.0, 43.3, 35.8, 30.1, 26.8, 23.0; **IR** ν_{max} [cm^{-1}] 2099 (N_3); **HRMS** (CI) *m/z:* $[M + H]^+$ Calcd. for $C_8H_{15}N_3OBr$ 248.0398; Found 248.0390.

Chemoselectivity studies on bifunctionalized model substrate 30

1-(4-Vinylphenyl)ethan-1-ol (44.5 mg, 300 µmol) was applied to three different reactions according to **Method B**, varying the equivalents of Zhdankin's reagent (**7**) and Et_4PBr (**1**). The results of the experiments are listed in Table S7.

Table S7 Chemoselectivity studies on bifunctionalized model substrate 30.

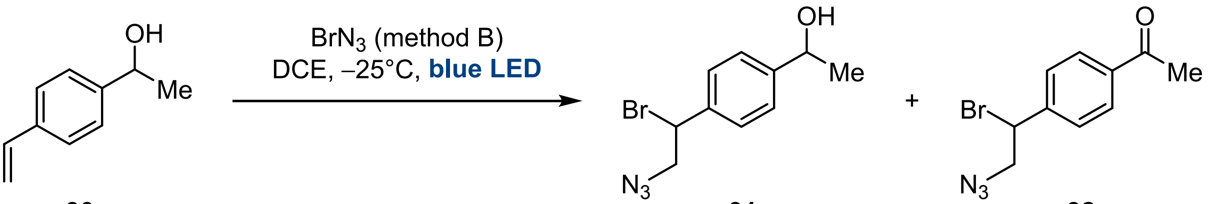

30 **31** **32**

Entry	Zhdankin's reagent / eq.	Et_4PBr / eq.	30	31	32
1	6.0	4.0	-	-	95% (76.5 mg, 285 µmol)
2	3.0	2.0	-	-	89% (71.9 mg, 268 µmol)
3	1.5	1.0	61% (27.7 mg, 183 µmol)	26% (21.4 mg, 79.2 µmol)	traces

1-(4-(2-Azido-1-bromoethyl)phenyl)ethan-1-ol (31)

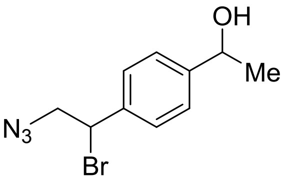

31

Column chromatography Petroleum ether/EtOAc = 10:1; **Appearance** colorless oil; **R$_f$** = 0.17 (petroleum ether/EtOAc = 4:1); **^{1}H NMR** (400 MHz, $CDCl_3$) δ [ppm] 7.47 – 7.34 (m, 4H), 5.01 (t, J = 7.3 Hz, 1H), 4.91 (q, J = 6.5 Hz, 2H), 3.87 (qd, J = 13.0, 7.3 Hz, 2H), 1.89 (s, 1H), 1.49 (d, J = 6.5 Hz, 3H); **^{13}C{^{1}H} NMR** (101 MHz, $CDCl_3$) δ [ppm] 147.1, 137.8, 128.0, 126.2, 70.1, 57.7, 50.9, 25.3; **IR** ν_{max} [cm^{-1}] 3360 (OH), 2099 (N_3); **GC-MS** (t_R 11.483 min) m/z $[M - N_2]^+$ Calcd. for $C_{10}H_{12}{}^{79}BrNO$ 241.0; Found 241.0; m/z $[M - OH]^+$ Calcd. for $C_{10}H_{11}{}^{79}BrN_3$ 250.1; Found 250.1; m/z $[M - N_2OH]^+$ Calcd. for $C_{10}H_{11}{}^{79}BrN$ 224.0; Found 224.0 and the corresponding ^{81}Br isotopes for the given fragments; m/z $[M - Br]^+$ Calcd. for $C_{10}H_{12}N_3O$ 190.1; Found 190.1.

1-(4-(2-Azido-1-bromoethyl)phenyl)ethan-1-one (32)

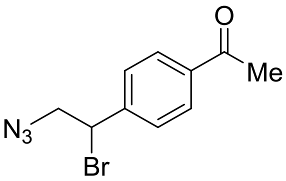

32

Column chromatography Petroleum ether/EtOAc = 10:1; **Appearance** colorless oil; **R$_f$** = 0.21 (petroleum ether/EtOAc = 4:1); **^{1}H NMR** (400 MHz, $CDCl_3$) δ [ppm] 7.97 (d, J = 8.4 Hz, 2H), 7.53 (d, J = 8.4 Hz, 2H), 5.02 (t, J = 7.2 Hz, 1H), 3.90 (qd, J = 13.0, 7.2 Hz, 2H), 2.61 (s, 3H); **^{13}C{^{1}H} NMR** (101 MHz, $CDCl_3$) δ [ppm] 197.3, 143.5, 137.7, 129.1, 128.2, 57.4, 49.7, 26.8; **IR** ν_{max} [cm^{-1}] 2100 (N_3); **HRMS** (CI) m/z: $[M + H]^+$ Calcd. for $C_{10}H_{11}N_3OBr$ 268.0085; Found 268.0088.

Competition experiment

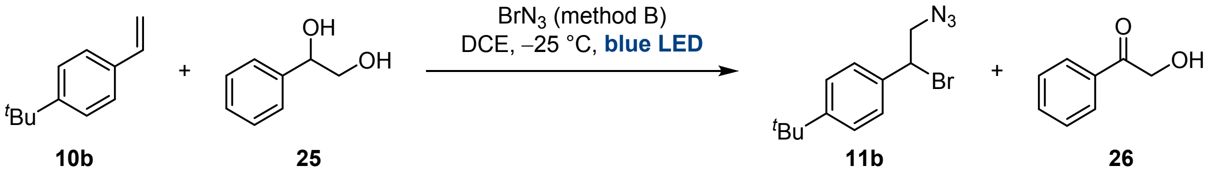

Scheme S4: Competition experiment to elucidate the selectivity of 1,2-bromoazidation of olefins versus oxidation of secondary alcohols.

A mixture of 1-(*tert*-butyl)-4-vinylbenzene (**10b**) (48.1 mg, 300 µmol) and 1-phenyl-1,2-ethanediol (**25**) (41.4 mg, 300 µmol) was applied to a reaction according to **Method B**. After purification by column chromatography (petroleum ether/EtOAc = 20:1) 1-(2-azido-1-bromoethyl)-4-(*tert*-butyl)benzene (**11b**) (54.1 mg, 192 µmol, 64%) and 2-hydroxy-1-phenylethan-1-one (**26**) (19.8 mg, 145 µmol, 49%) were isolated.

4. X-ray crystal structure analysis of compound 10i

Sample preparation

In a 5 mL glass vial, the purified steroid **10i** was layered with 1 mL petroleum ether. Then CH_2Cl_2 was slowly added until the sample was completely dissolved. The vial was sealed with a plastic cap with a small hole to allow CH_2Cl_2 to evaporate. The sample was kept in the refrigerator until a crystal reached a sufficient size.

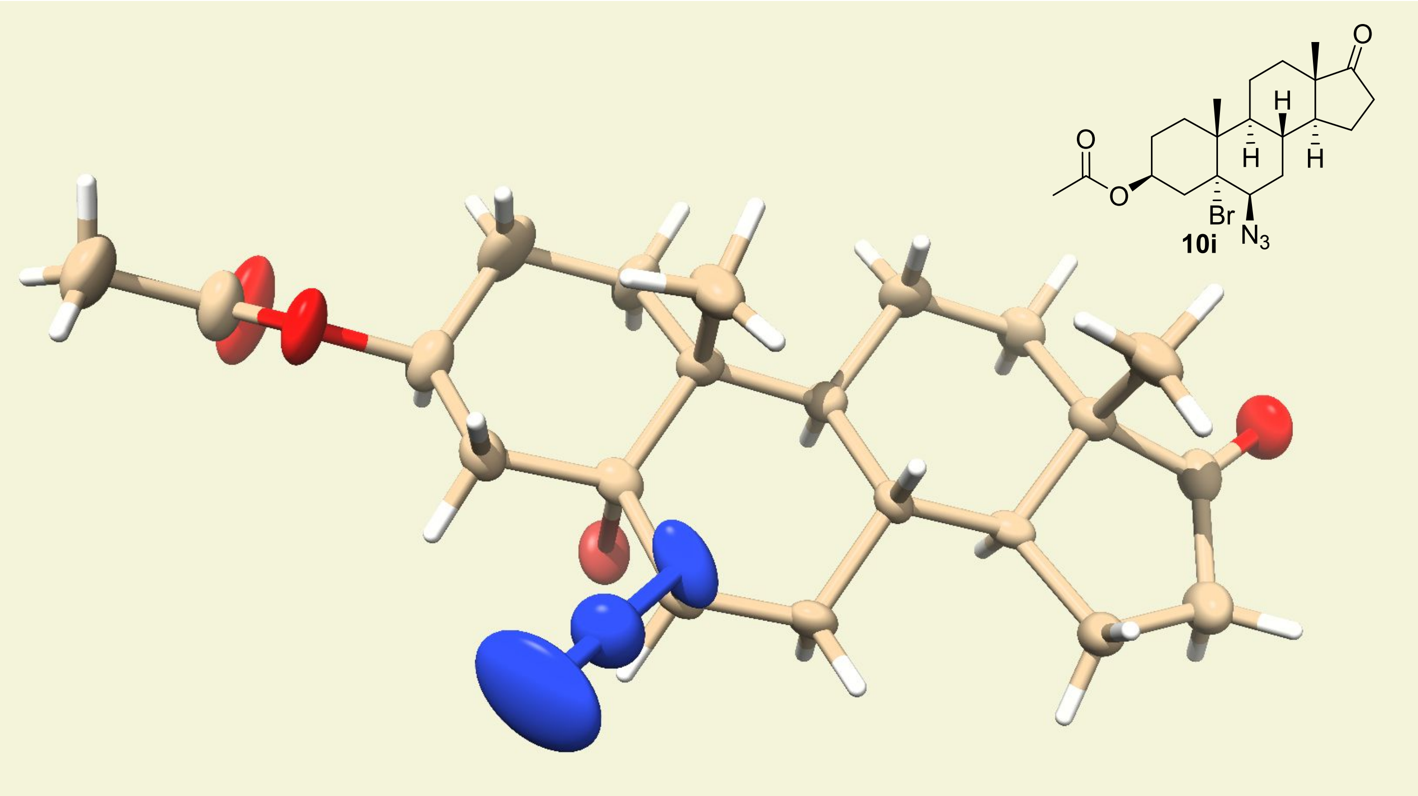

Figure S5: Thermal ellipsoid plot for the crystal structure of compound 10i (probability level 50%).

**Identification code**	VG103c
**Chemical formula**	$C_{21}H_{30}BrN_3O_3$
**Formula weight**	452.39 g/mol
**Temperature**	200(2) K
**Wavelength**	0.71073 Å
**Crystal size**	0.160 x 0.280 x 0.600 mm
**Crystal system**	monoclinic
**Space group**	P 1 21 1
**Unit cell dimensions**	a = 8.4901(14) Å α = 90°
	b = 6.8845(11) Å β = 94.904(6)°
	c = 18.313(3) Å γ = 90°
**Volume**	1066.5(3) $Å^3$
**Z**	12
**Density (calculated)**	1.409 g/cm^3
**Absorption coefficient**	1.953 mm^{-1}
**F(000)**	472
**Theta range for data collection**	2.41 to 25.86°
**Index ranges**	-10<=h<=10, -8<=k<=8, -22<=l<=22
**Reflections collected**	19980
**Independent reflections**	4076 [R(int) = 0.0995]
**Coverage of independent reflections**	98.8%
**Absorption correction**	Multi-Scan
**Max. and min. transmission**	0.7450 and 0.3870
**Structure solution technique**	direct methods
**Structure solution program**	SHELXT 2014/5 (Sheldrick, 2014)
**Refinement method**	Full-matrix least-squares on F^2
**Refinement program**	SHELXL-2018/3 (Sheldrick, 2018)
**Function minimized**	$\Sigma w(F_o^2 - F_c^2)^2$
**Data / restraints / parameters**	4076 / 1 / 256
**Goodness-of-fit on F^2**	0.987
**Final R indices**	3249 data; $I>2\sigma(I)$ R1 = 0.0472, wR2 = 0.0856
	all data R1 = 0.0682, wR2 = 0.0916
**Weighting scheme**	$w=1/[\sigma^2(F_o^2)+(0.0211P)^2+0.3894P]$<br>where $P=(F_o^2+2F_c^2)/3$
**Absolute structure parameter**	0.049(10)
**Largest diff. peak and hole**	0.525 and -0.425 $eÅ^{-3}$

R.M.S. deviation from mean 0.060 eÅ^{-3}

5. References

(S1) Leonard, J.; Lygo, B.; Procter, G., Eds. *Praxis der organischen Chemie*: *Ein Handbuch;* VCH, 1996.

(S2) Hendrick, C. E.; Bitting, K. J.; Cho, S.; Wang, Q. Site-Selective Copper-Catalyzed Amination and Azidation of Arenes and Heteroarenes via Deprotonative Zincation. *J. Am. Chem. Soc.* **2017**, *139* (33), 11622–11628.

(S3) Cox, C. D.; Dudkin, V.; Kern, J.; Layton, M. E.; Raheem, I. T. PYRIMIDINE PDE10 INHIBITORS. WO2013028590 (A1), 2013.

(S4) Terada, Y.; Arisawa, M.; Nishida, A. Cycloisomerization promoted by the combination of a ruthenium-carbene catalyst and trimethylsilyl vinyl ether, and its application in the synthesis of heterocyclic compounds: 3-methylene-2,3-dihydroindoles and 3-methylene-2,3-dihydrobenzofurans. *Angew. Chem. Int. Ed.* **2004**, *43* (31), 4063–4067.

(S5) Mei, L.; Wei, Y.; Xu, Q.; Shi, M. Diastereo- and Enantioselective Construction of Oxindole-Fused Spirotetrahydrofuran Scaffolds through Palladium-Catalyzed Asymmetric [3+2] Cycloaddition of Vinyl Cyclopropanes and Isatins. *Organometallics* **2013**, *32* (12), 3544–3556.

(S6) Mori, K.; Tomioka, H. Pheromone Synthesis, CXL. Synthesis of Four Macrolide Pheromones to Define the Scope and Limitation of Enzymatic Macrolactonization. *Liebigs Ann. Chem.* **1992**, *1992* (10), 1011–1017.

(S7) He, C.-L.; Feng, Z.; Li, Y.; Zhou, M.; Zhao, L.; Shan, S.; Wang, M.; Chen, X.; Wang, X.-S.; Zou, G. Improved enantioselectivity in thiol–ene photopolymerization of sulphur-containing polymers with circularly polarized luminescence. *Polym. Chem.* **2021**, *12* (16), 2433–2438.

(S8) Bräse, S.; Gil, C.; Knepper, K.; Zimmermann, V. Organic azides: an exploding diversity of a unique class of compounds. *Angew. Chem. Int. Ed.* **2005**, *44* (33), 5188–5240.

(S9) Alazet, S.; Preindl, J.; Simonet-Davin, R.; Nicolai, S.; Nanchen, A.; Meyer, T.; Waser, J. Cyclic Hypervalent Iodine Reagents for Azidation: Safer Reagents and Photoredox-Catalyzed Ring Expansion. *J. Org. Chem.* **2018**, *83* (19), 12334–12356.

(S10) Mitrochkine, A. A.; Blain, I.; Bit, C.; Canlet, C.; Pierre, S.; Courtieu, J.; Réglier, M. Enantioselective Synthesis of cis - and trans -2(S)-Amino-1- d -indane: Debrominative [1,2]-Hydride Shift Rearrangement by Reduction of cis -2-Azido-1-bromoindane with LiAlD 4. *J. Org. Chem.* **1997**, *62* (18), 6204–6209.

(S11) Valiulin, R. A.; Mamidyala, S.; Finn, M. G. Taming chlorine azide: access to 1,2-azidochlorides from alkenes. *J. Org. Chem.* **2015**, *80* (5), 2740–2755.

(S12) Cambie, R. C.; Jurlina, J. L.; Rutledge, P. S.; Swedlund, B. E.; Woodgate, P. D. Reactions of iodine(I) azide with αβ-unsaturated carbonyl compounds. *J. Chem. Soc., Perkin Trans. 1* **1982**, (0), 327–333.

(S13) Dong, X.; Roeckl, J. L.; Waldvogel, S. R.; Morandi, B. Merging shuttle reactions and paired electrolysis for reversible vicinal dihalogenations. *Science* **2021**, *371* (6528), 507–514.

(S14) Ryu, I.; Matsubara, H.; Yasuda, S.; Nakamura, H.; Curran, D. P. Phase-vanishing reactions that use fluorous media as a phase screen. Facile, controlled bromination of alkenes by dibromine and dealkylation of aromatic ethers by boron tribromide. *J. Am. Chem. Soc.* **2002**, *124* (44), 12946–12947.

(S15) Kösel, T.; Schulz, G.; Dräger, G.; Kirschning, A. Photochemical Transformations with Iodine Azide after Release from an Ion-Exchange Resin. *Angew. Chem. Int. Ed.* **2020**, *59* (30), 12376–12380.

(S16) Giorgio, E.; Viglione, R. G.; Zanasi, R.; Rosini, C. Ab initio calculation of optical rotatory dispersion (ORD) curves: a simple and reliable approach to the assignment of the molecular absolute configuration. *J. Am. Chem. Soc.* **2004**, *126* (40), 12968–12976.

(S17) Kolehmainen, E.; Laihia, K.; Korvola, J.; Kauppinen, R.; Pitkänen, M.; Mannila, B.; Mannila, E. Multinuclear NMR study of 1,3,3-trimethylbicyclo[2.2.1]heptan-2-one (fenchone) and its six monochlorinated derivatives. *Magn. Reson. Chem.* **1990**, *28* (9), 812–816.

(S18) Tuktarov, A. R.; Korolev, V. V.; Tulyabaev, A. R.; Popod'ko, N. R.; Khalilov, L. M.; Dzhemilev, U. M. Synthesis of optically active spiro homo- and methanofullerenes. *Tetrahedron Lett.* **2011**, *52* (7), 834–836.

(S19) He, C.; Ma, F.; Zhang, W.; Tong, R. Reinvestigating FeBr3-Catalyzed Alcohol Oxidation with H2O2: Is a High-Valent Iron Species (HIS) or a Reactive Brominating Species (RBS) Responsible for Alcohol Oxidation? *Org. Lett.* **2022**, *24* (19), 3499–3503.

(S20) William, J. M.; Kuriyama, M.; Onomura, O. Boronic Acid-Catalyzed Selective Oxidation of 1,2-Diols to α-Hydroxy Ketones in Water. *Adv. Synth. Catal.* **2014**, *356* (5), 934–940.

(S21) Saleh, S. A.; Hazra, A.; Hajra, S. Regioselective Hydroperoxylation of Aziridines and Epoxides Only with Aqueous Hydrogen Peroxide. *Adv. Synth. Catal.* **2022**, *364* (2), 391–404.

(S22) Dehnicke, K.; Ruschke, P. Die Ultraviolettspektren der Halogenazide ClN 3 , BrN 3 und IN 3 / The Ultraviolet Spectra of the Halogen Azides ClN 3 , BrN 3 and IN 3. *Z. Naturforsch. B* **1978**, *33* (7), 750–752.

(S23) Strehl, J.; Abraham, M. L.; Hilt, G. Linear Paired Electrolysis-Realising 200 % Current Efficiency for Stoichiometric Transformations-The Electrochemical Bromination of Alkenes. *Angew. Chem. Int. Ed.* **2021**, *60* (18), 9996–10000.

6. Attachments: ^{1}H NMR, $^{13}C\{^{1}H\}$ NMR and IR spectra

(1*R**,2*R**)-2-Azido-1-bromo-2,3-dihydro-1*H*-indene (*trans*-11a)

^{1}H NMR (400 MHz, $CDCl_3$)

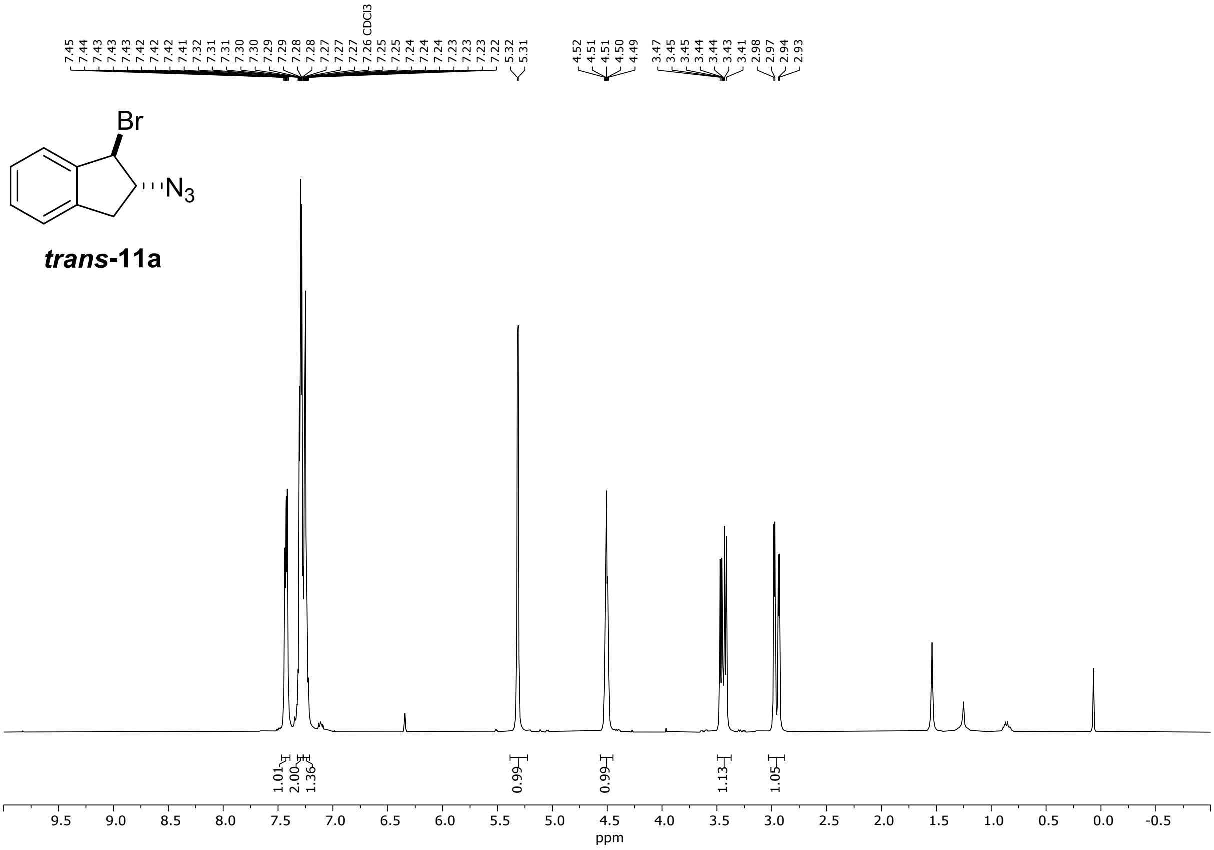

$^{13}C\{^{1}H\}$ NMR (101 MHz, $CDCl_3$)

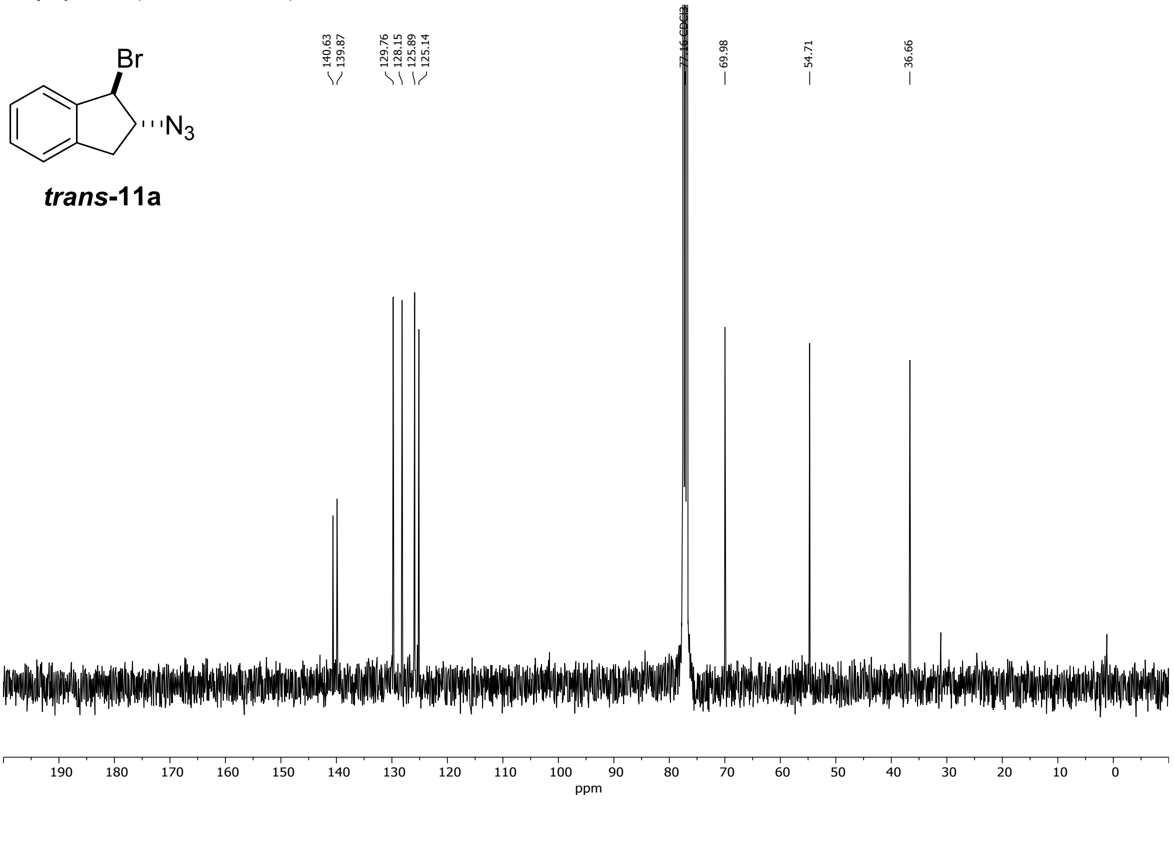

FT-IR

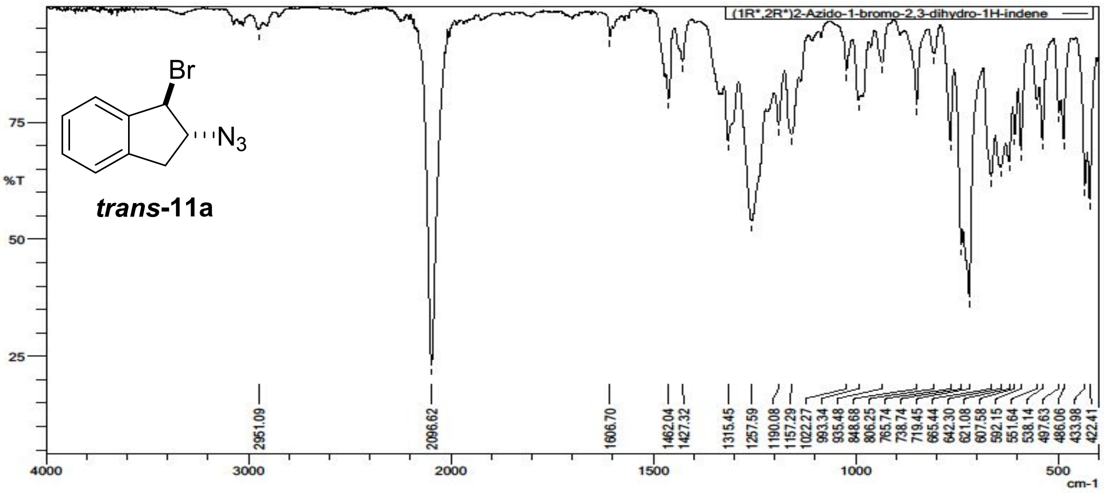

(1*R**,2*S**)-2-Azido-1-bromo-2,3-dihydro-*1H*-indene (*cis*-11a)

¹H NMR (400 MHz, $CDCl_3$)

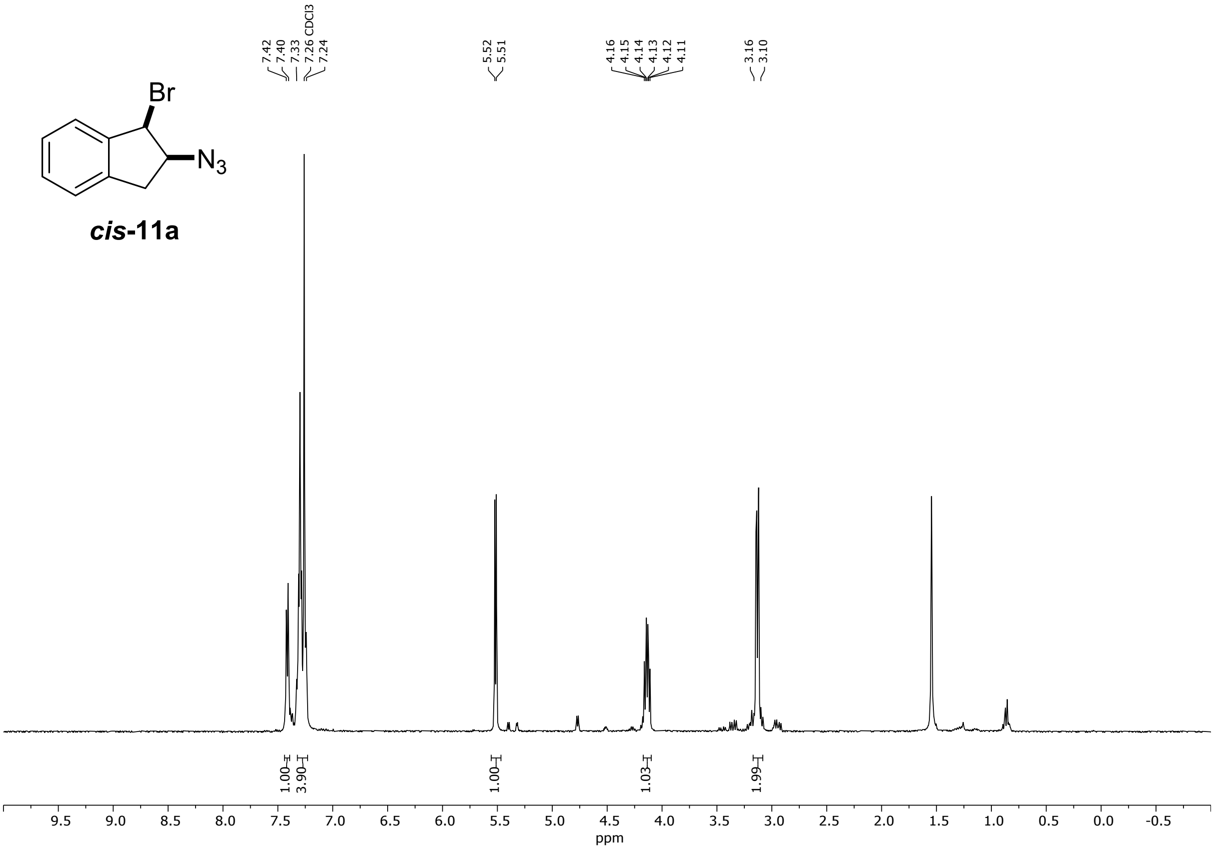

$^{13}C\{^{1}H\}$ **NMR** (101 MHz, $CDCl_3$)

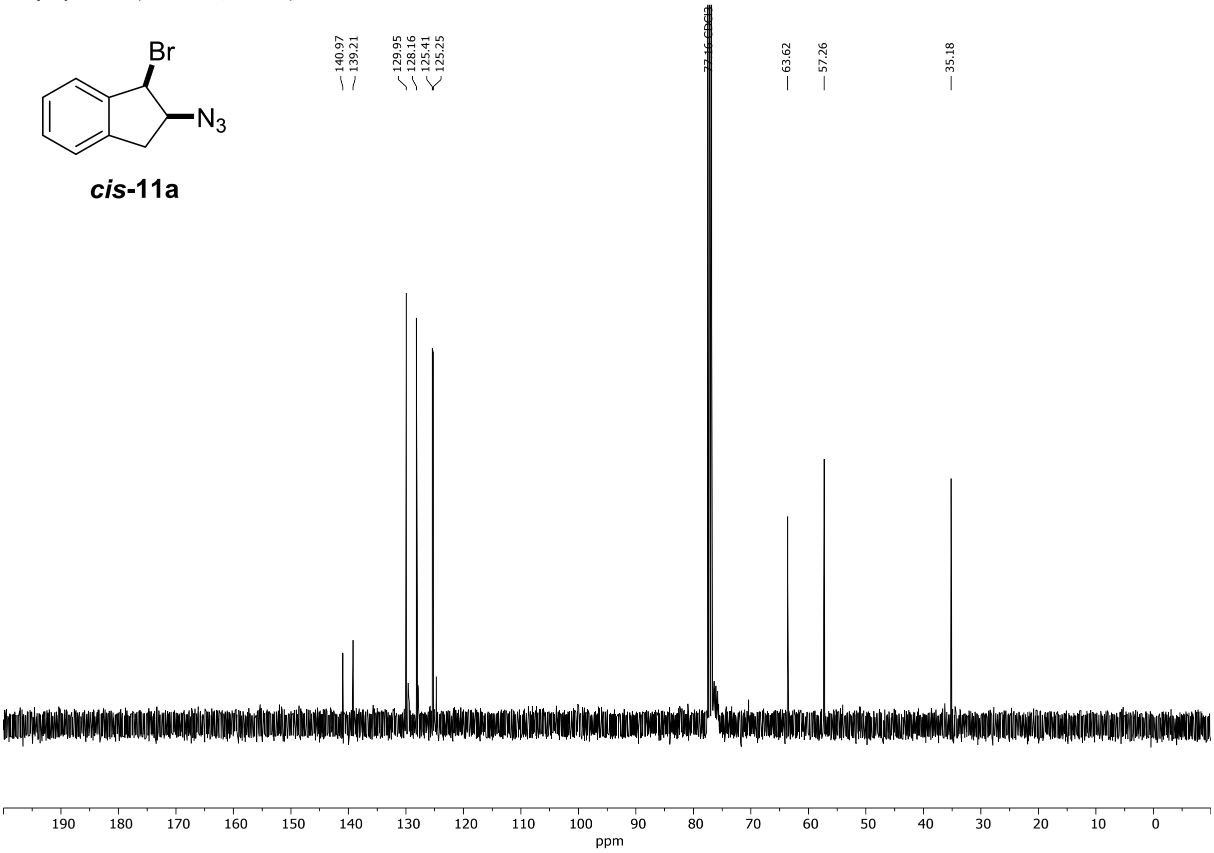

FT-IR

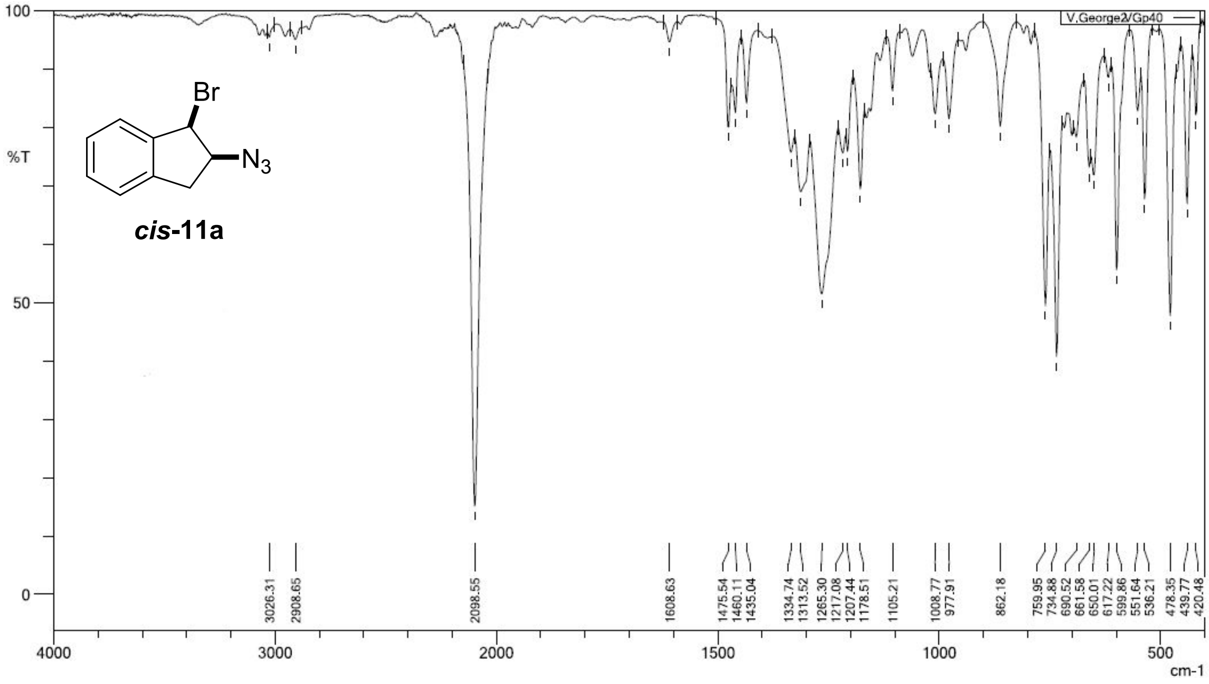

1-(2-Azido-1-bromoethyl)-4-(*tert*-butyl)benzene (11b)

^{1}H NMR (400 MHz, $CDCl_3$)

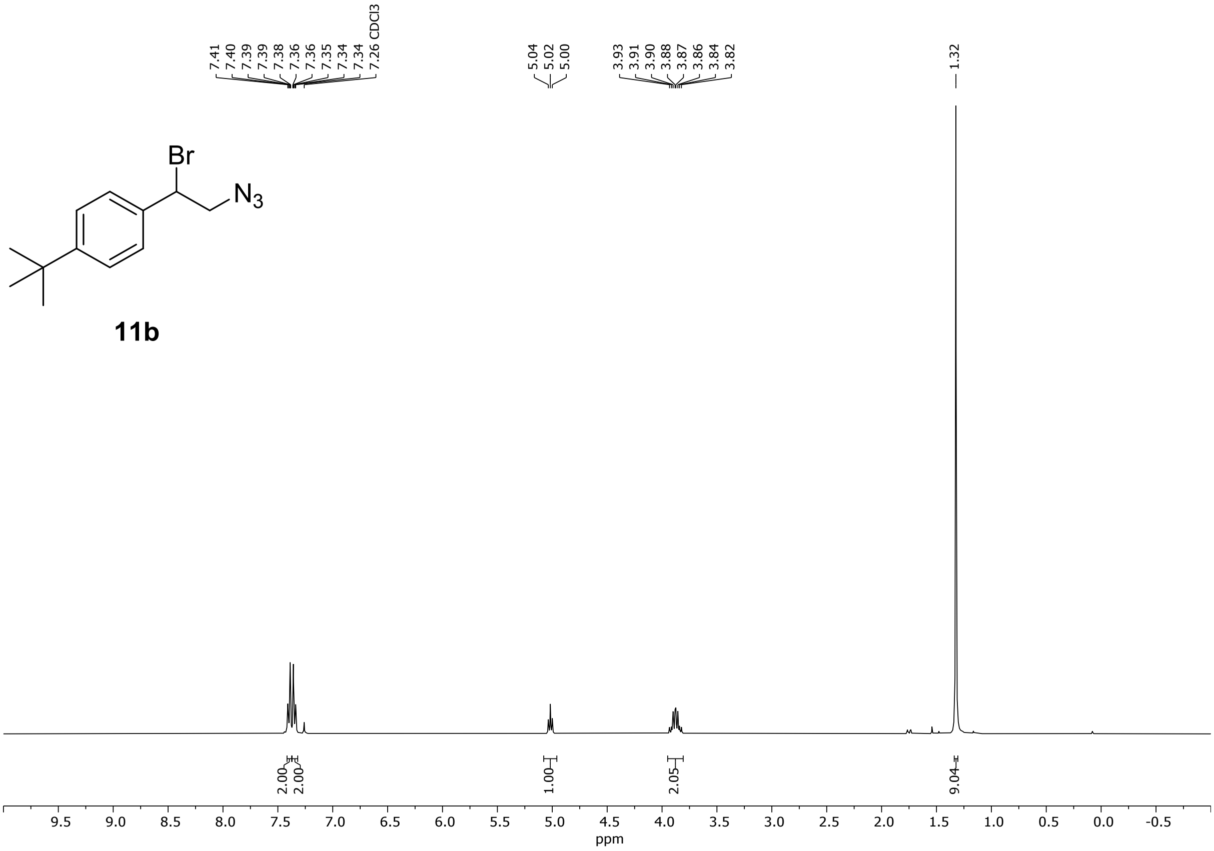

^{13}C{^{1}H} NMR (101 MHz, $CDCl_3$)

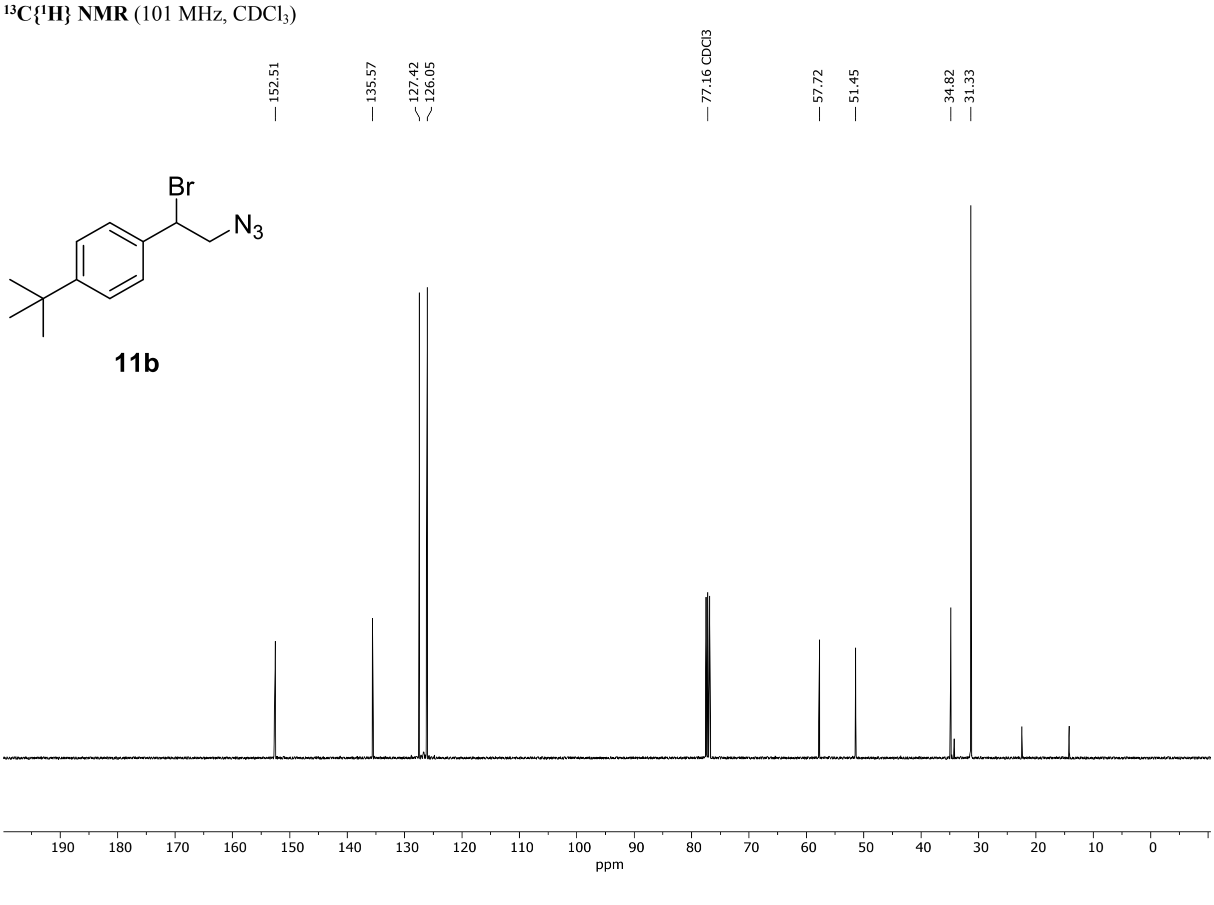

FT-IR

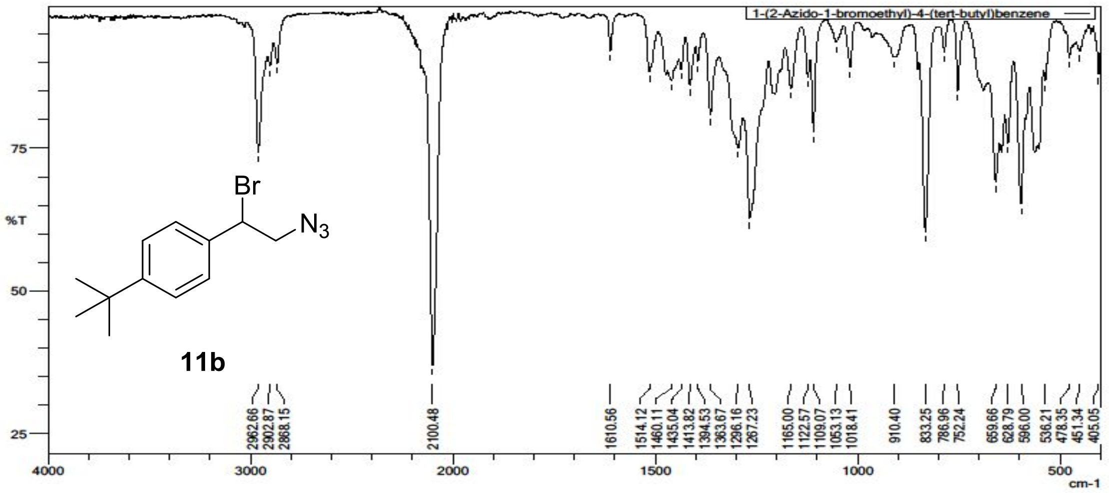

(4-Azido-3-bromobutyl)benzene (11c)

^{1}H NMR (400 MHz, $CDCl_3$)

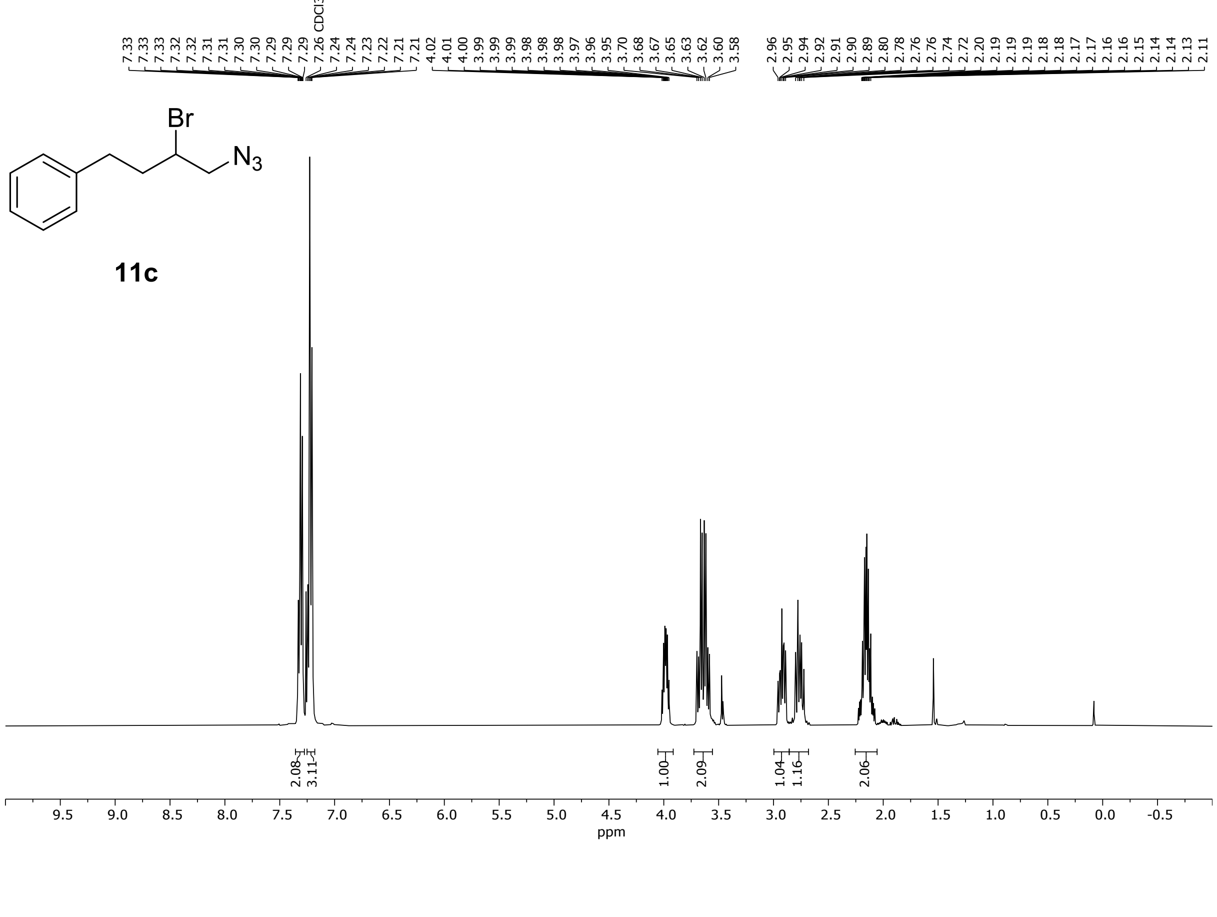

$^{13}C\{^{1}H\}$ NMR (101 MHz, $CDCl_3$)

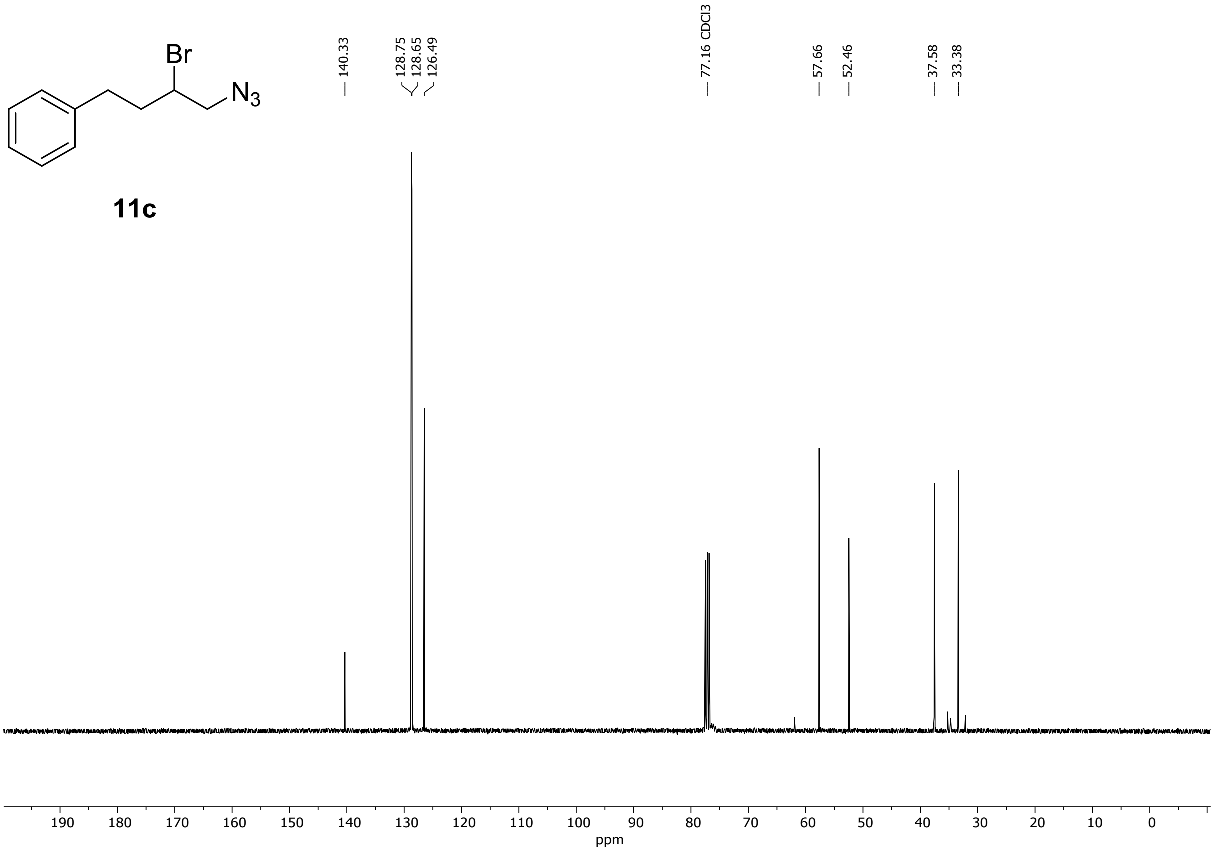

FT-IR

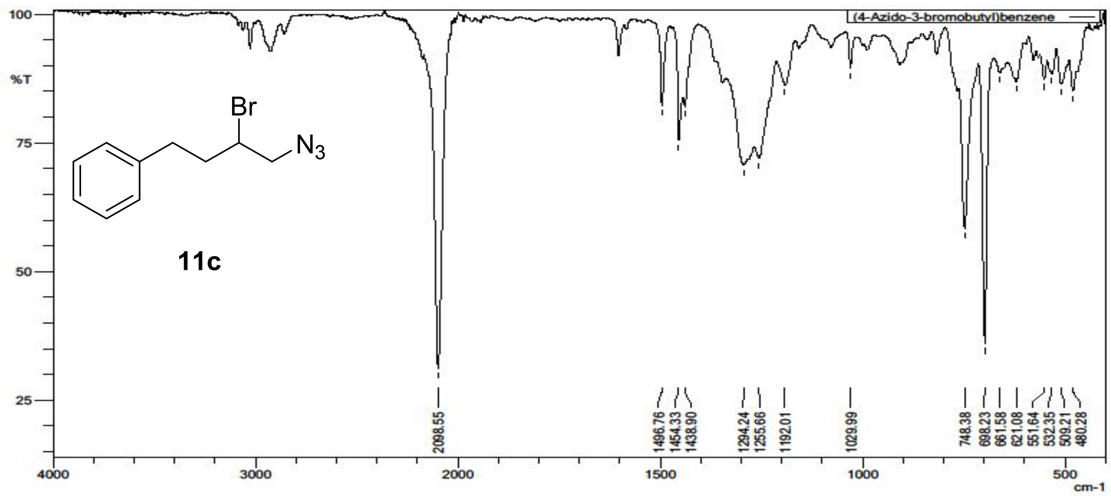

1-Azido-2-bromooctane (11d)

^{1}H NMR (400 MHz, $CDCl_3$)

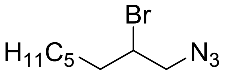

11d

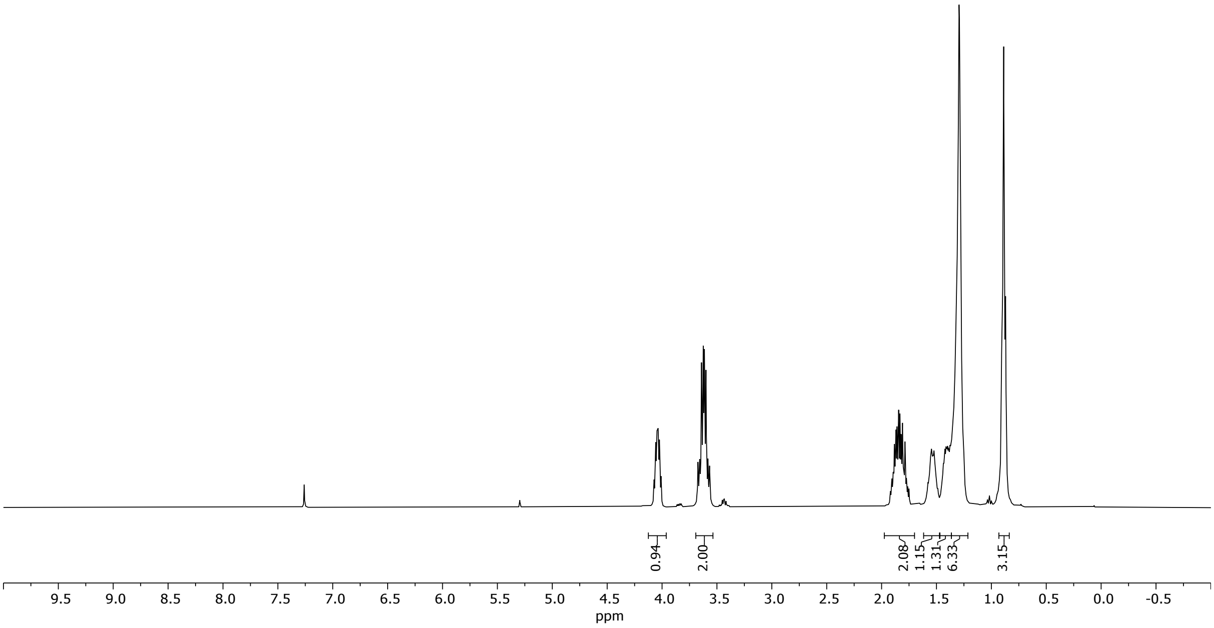

^{13}C{^{1}H} NMR (101 MHz, $CDCl_3$)

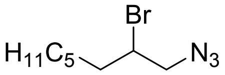

11d

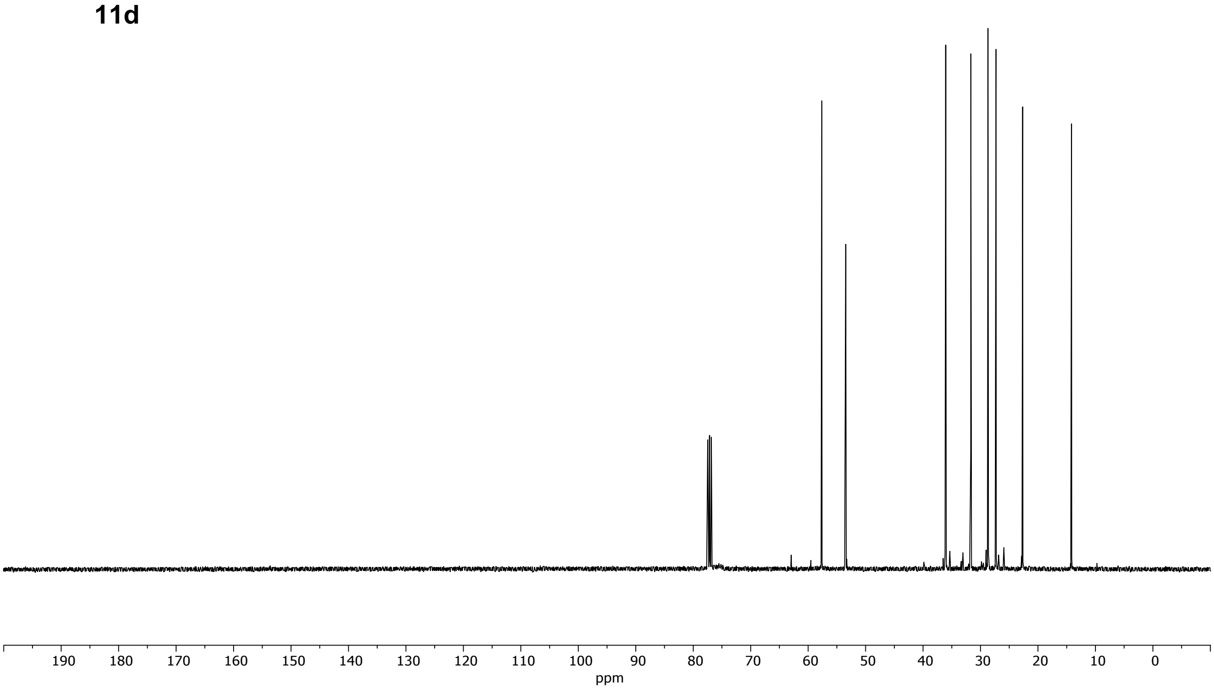

FT-IR

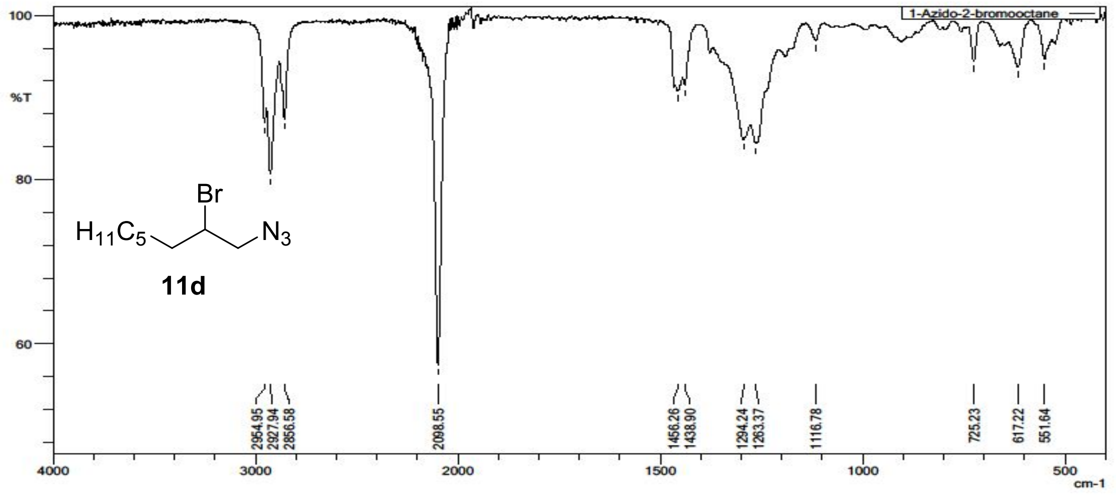

(1*S**,2*R**,3*S**,4*R**)-2-Azido-3-bromobicyclo[2.2.1]heptane (*cis*-11e)

^{1}H NMR (400 MHz, $CDCl_3$)

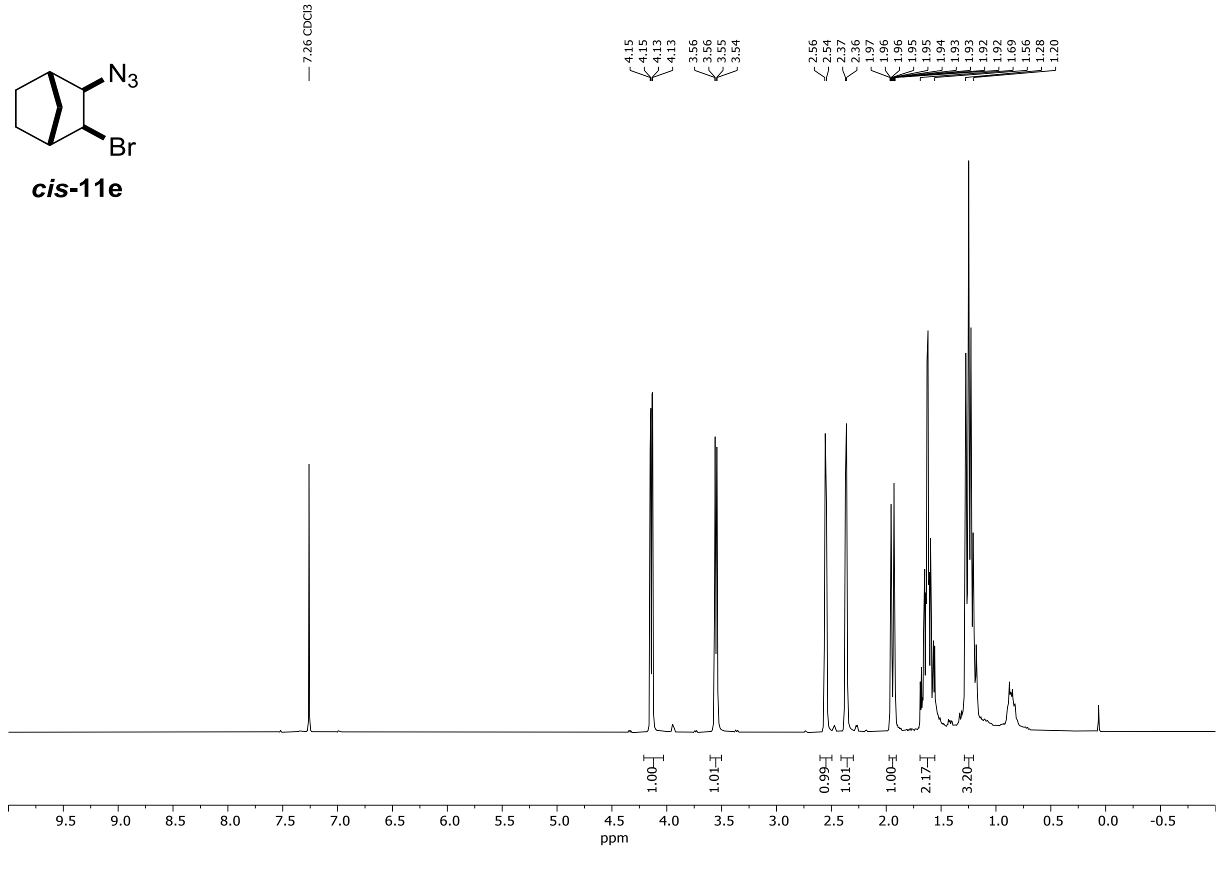

^{13}C{^{1}H} NMR (101 MHz, $CDCl_3$)

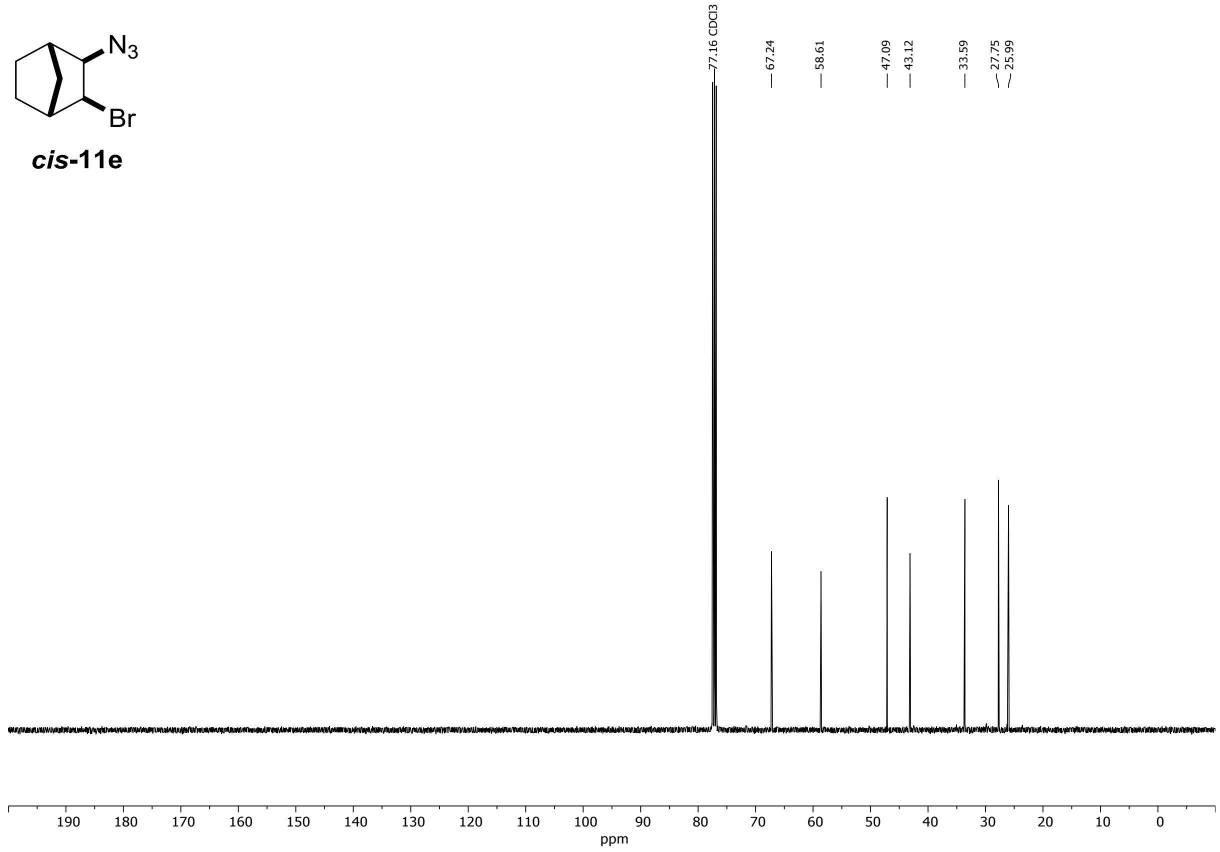

FT-IR

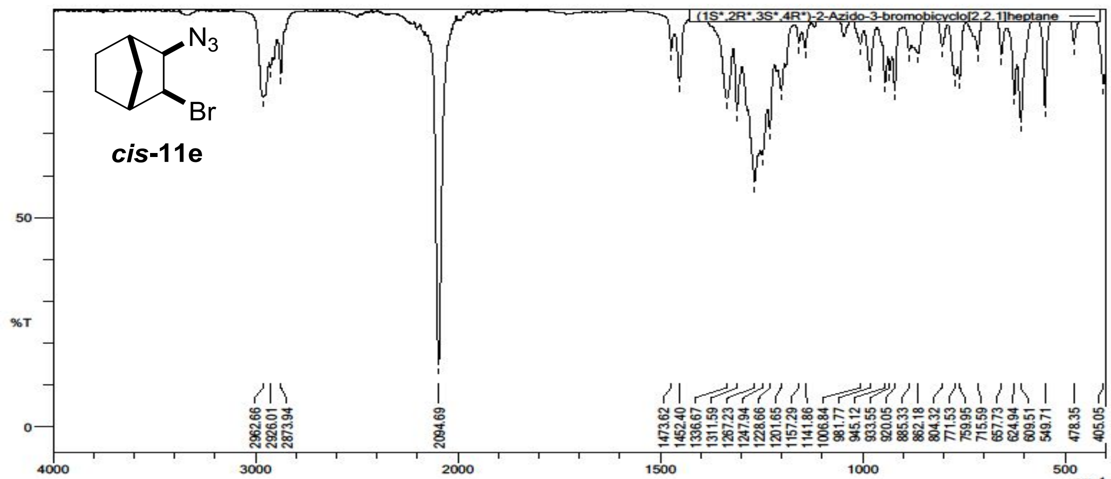

(1*S,2*R**,3*R**,4*R**)-2-Azido-3-bromobicyclo[2.2.1]heptane (*trans*-11e)**

^{1}H NMR (400 MHz, $CDCl_3$)

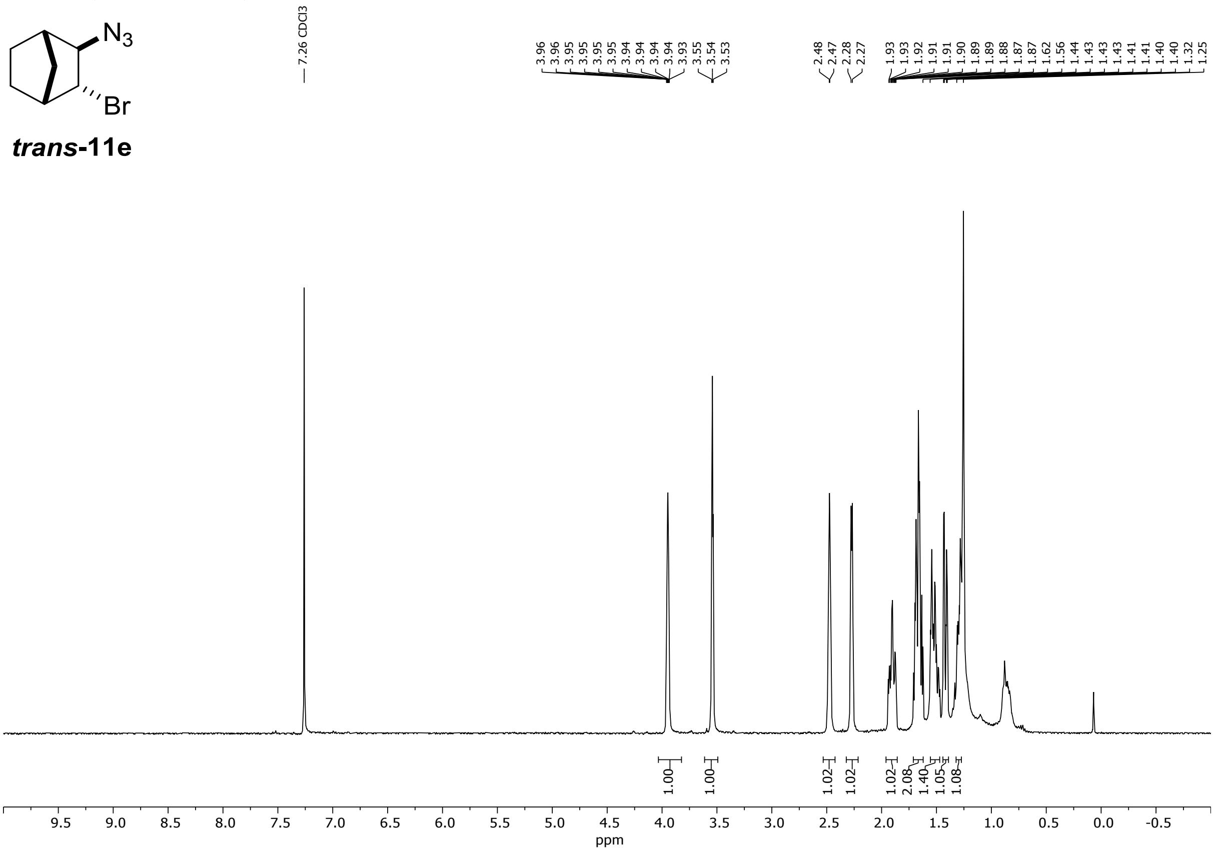

^{13}C{^{1}H} NMR (101 MHz, $CDCl_3$)

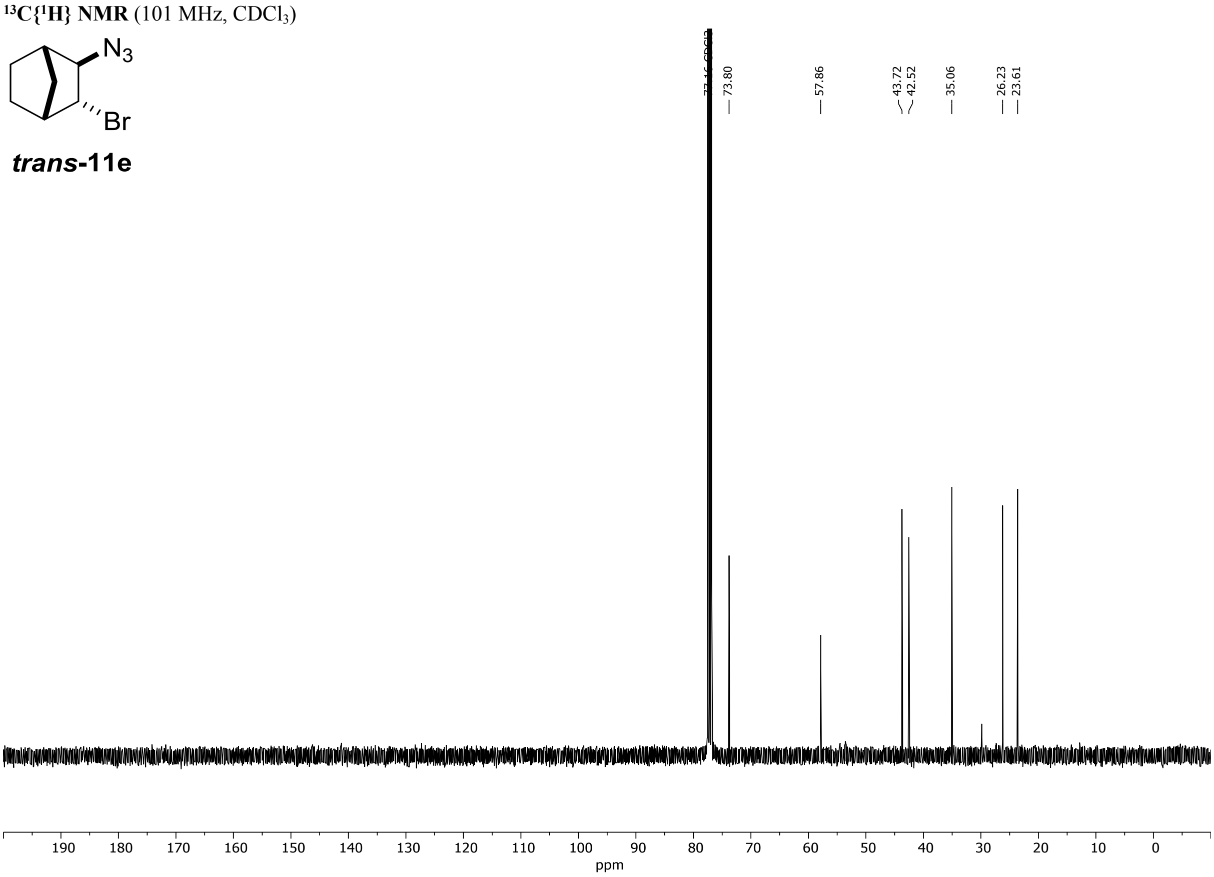

FT-IR

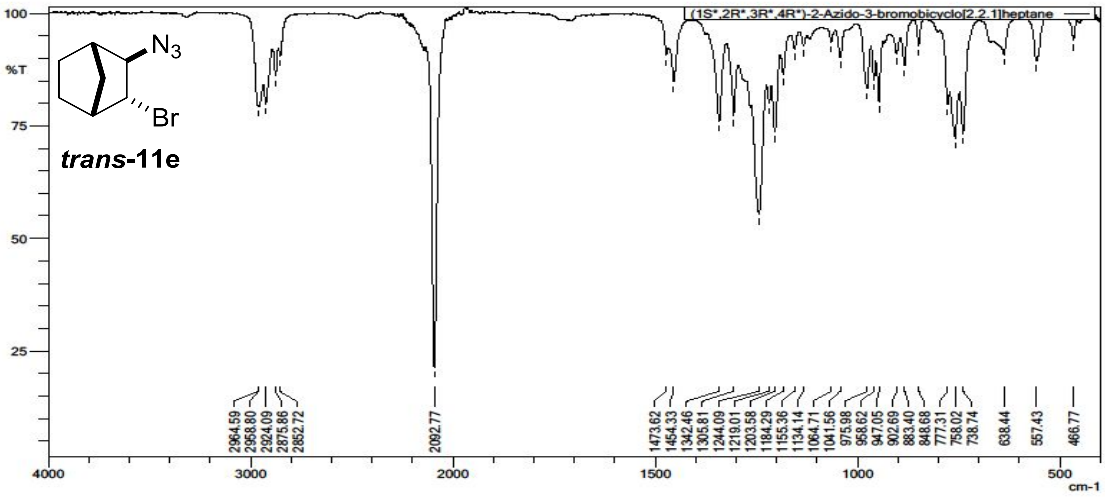

(1*R*,2*S*,5*S*)-2-(Azidomethyl)-2-bromo-6,6-dimethylbicyclo[3.1.1]heptane (11f)

^{1}H NMR (400 MHz, $CDCl_3$)

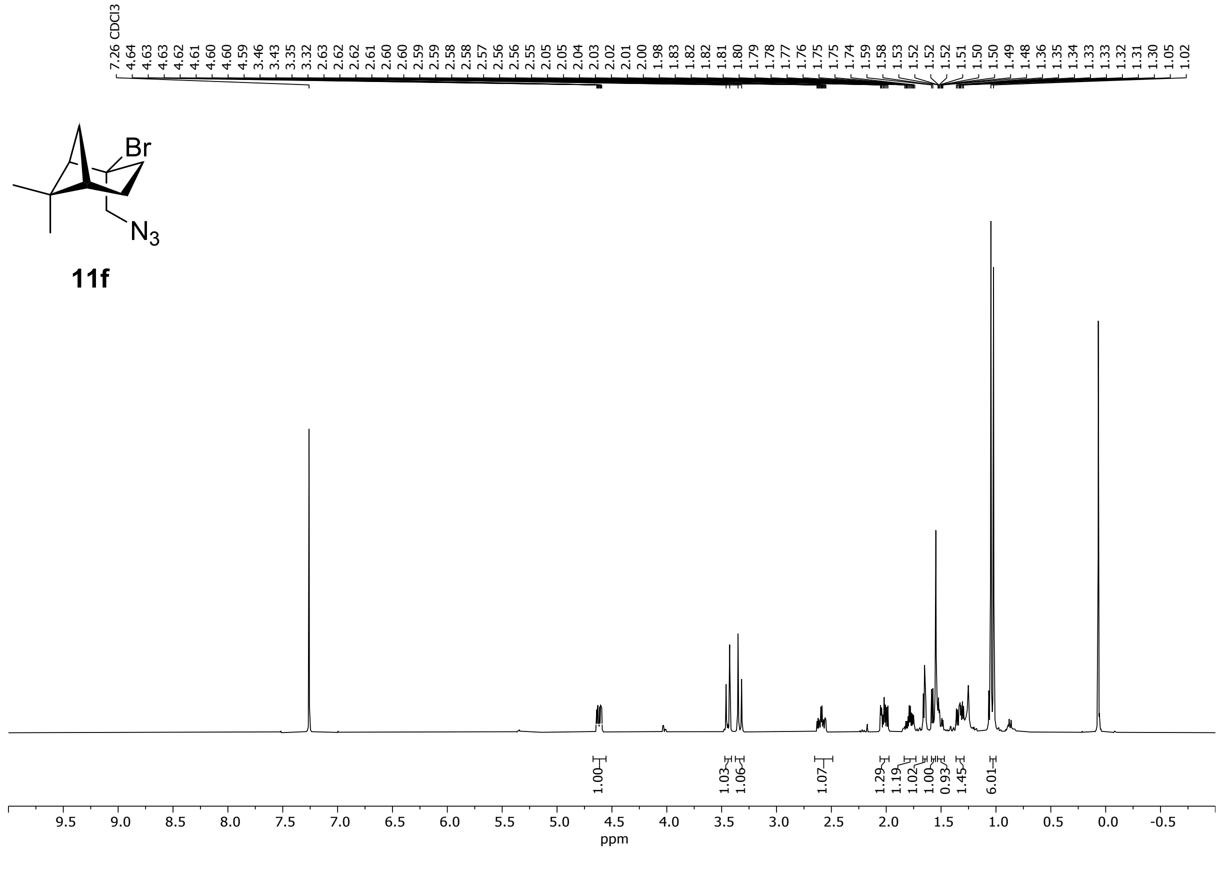

$^{13}C\{^1H\}$ **NMR** (101 MHz, CDCl$_3$)

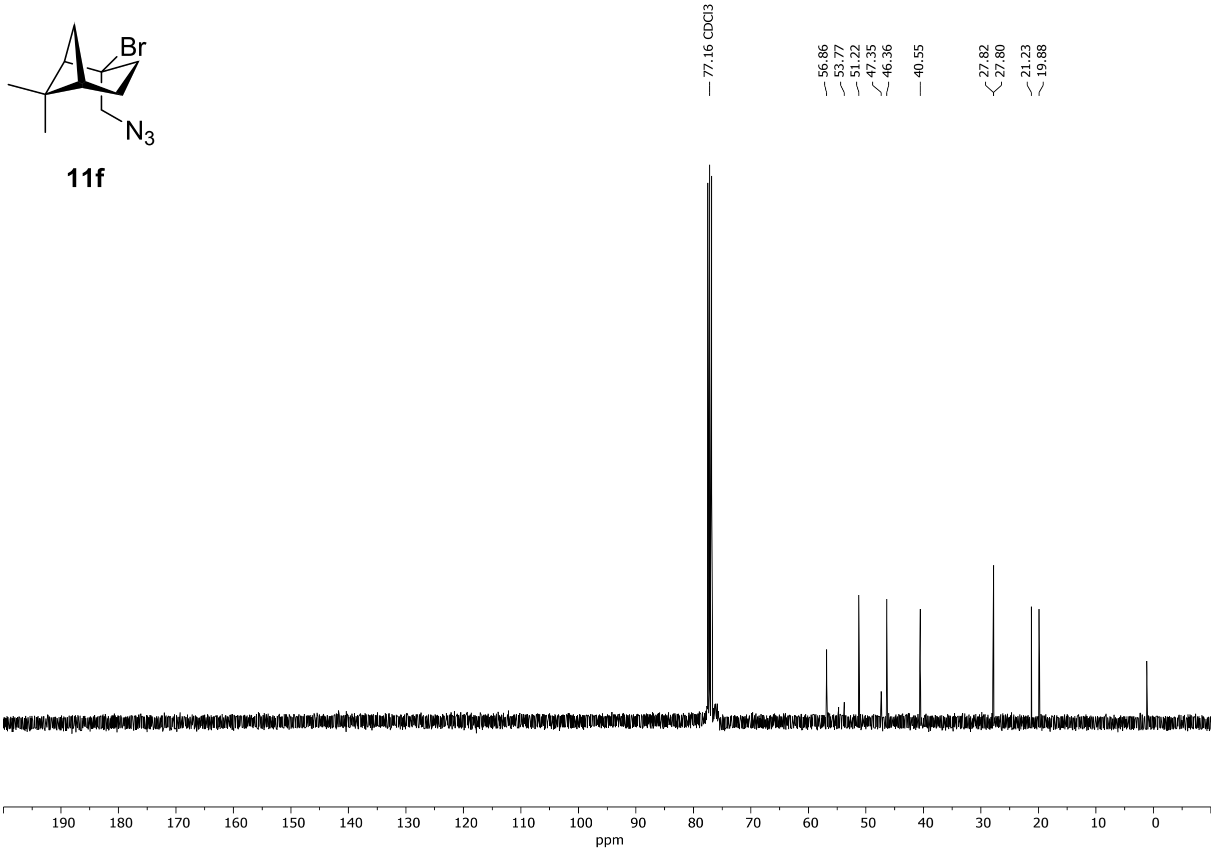

FT-IR

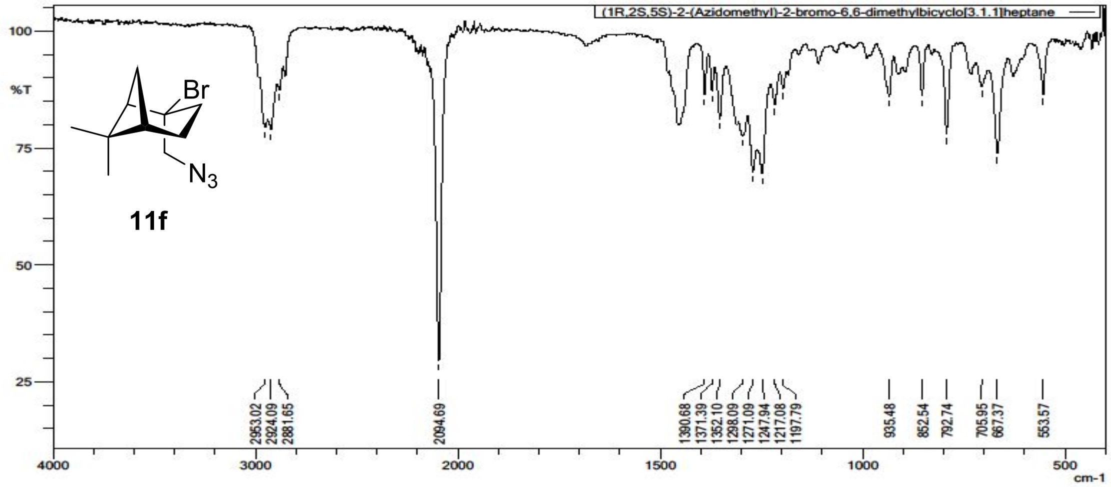

Ethyl (2*S**,3*S**)-2-azido-3-bromo-3-(thiazol-4-yl)propanoate (11g)

^{1}H NMR (400 MHz, $CDCl_3$)

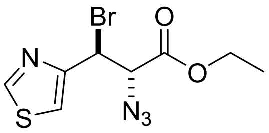

11g

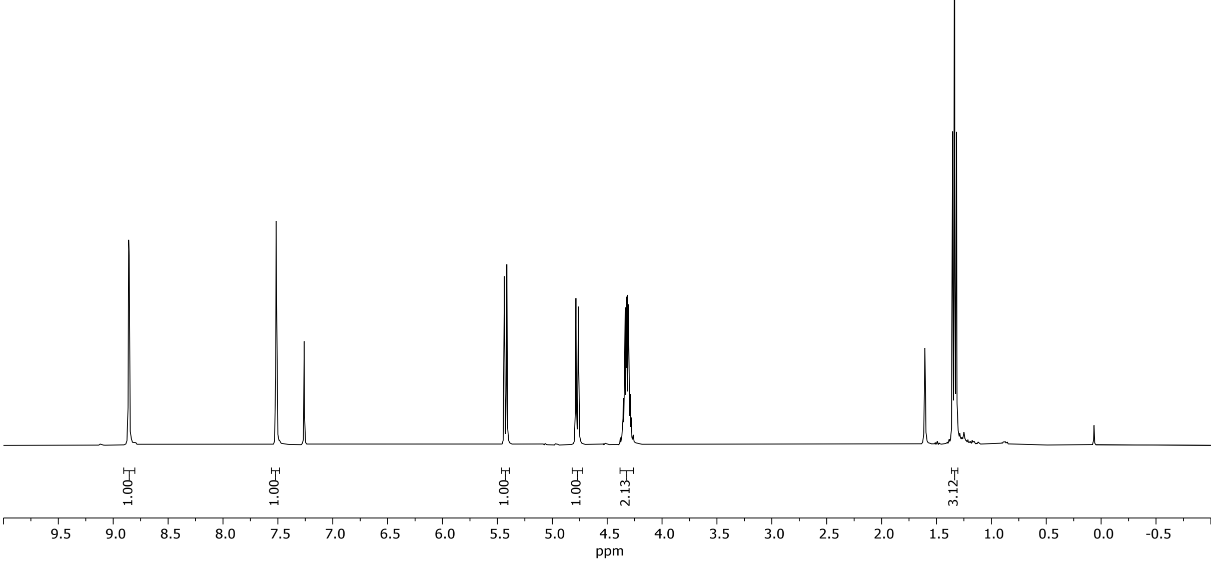

^{13}C{^{1}H} NMR (101 MHz, $CDCl_3$)

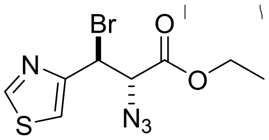

11g

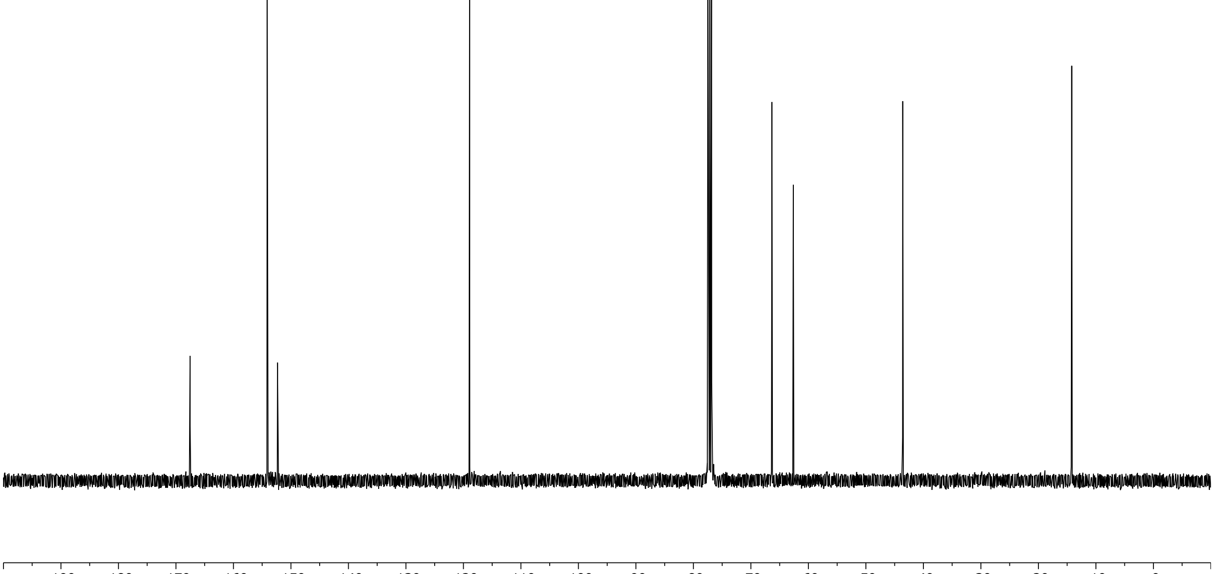

FT-IR

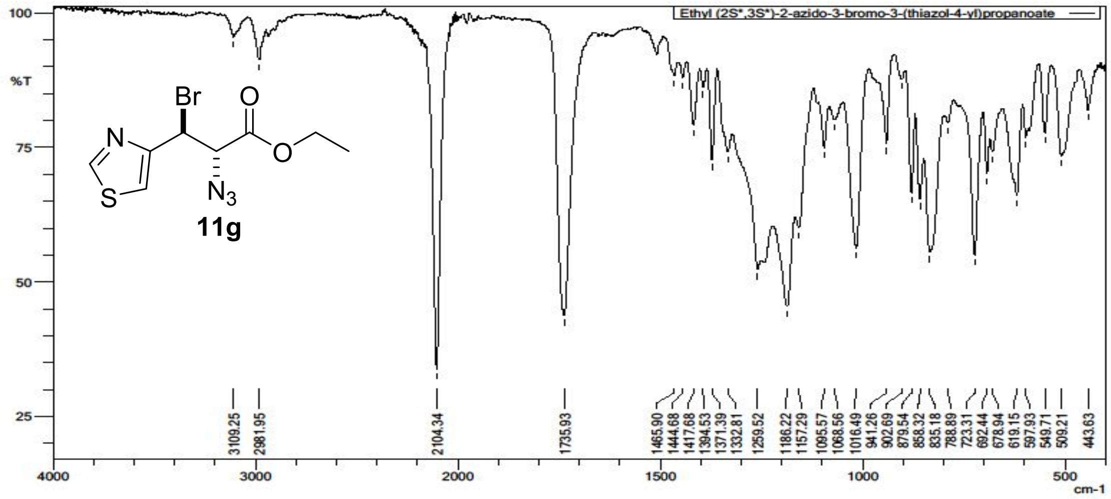

(2*S**,3*R**)-Methyl 2-azido-3-bromo-3-phenylpropanoate (11h)

^{1}H NMR (400 MHz, $CDCl_3$)

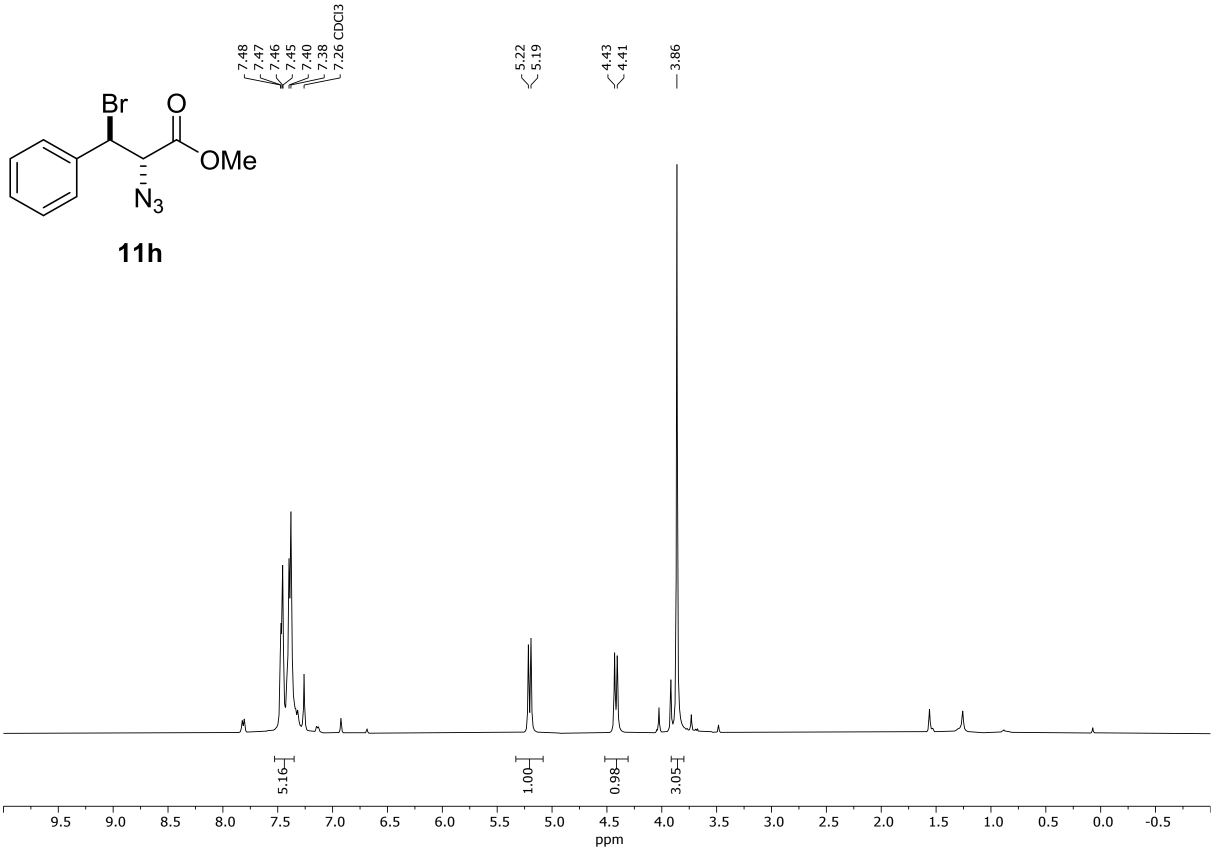

$^{13}C\{^{1}H\}$ **NMR** (101 MHz, $CDCl_3$)

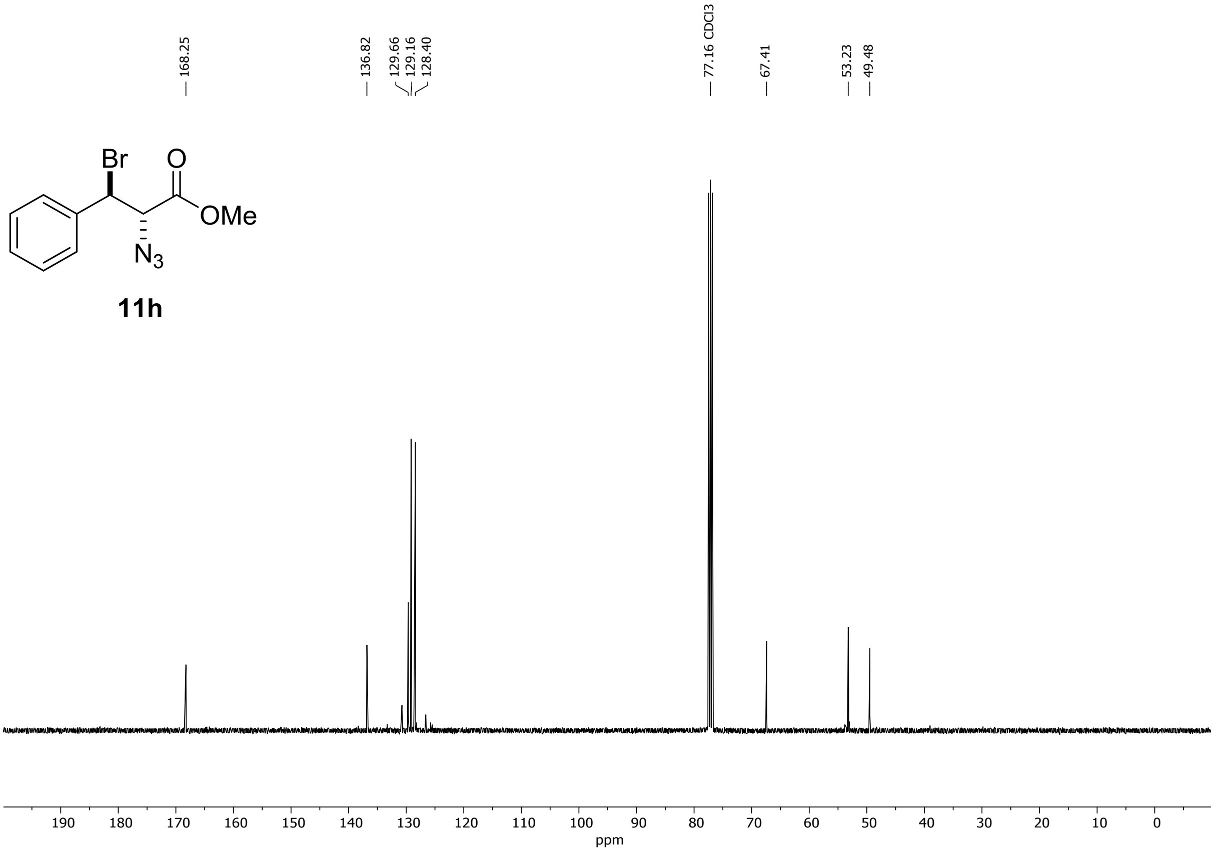

FT-IR

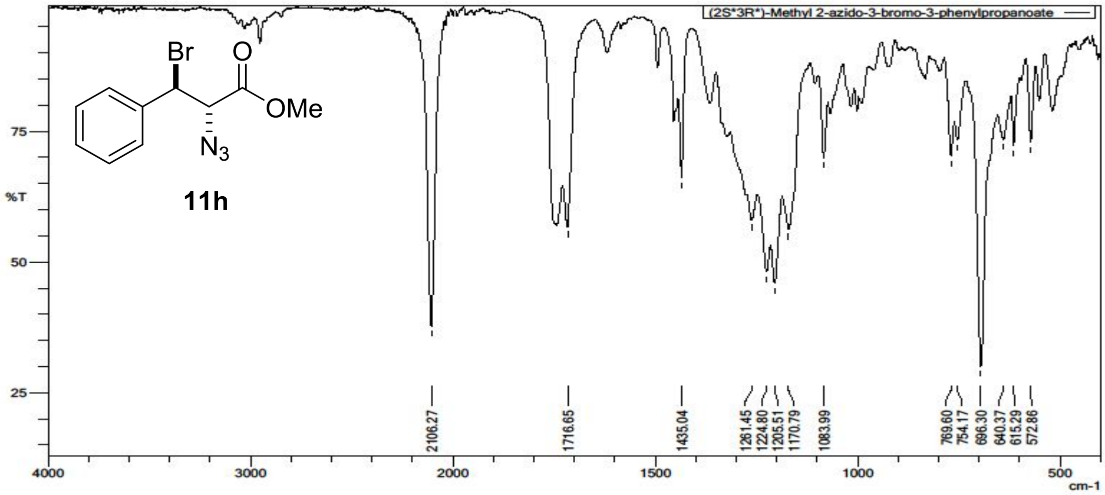

(3*S*,5*R*,6*R*,8*S*,9*S*,10*R*,13*S*,14*S*)-6-Azido-5-bromo-10,13-dimethyl-17-oxohexadecahydro-1*H*-cyclopenta[*a*]phenanthren-3-yl acetate (11i)

^{1}H NMR (400 MHz, $CDCl_3$)

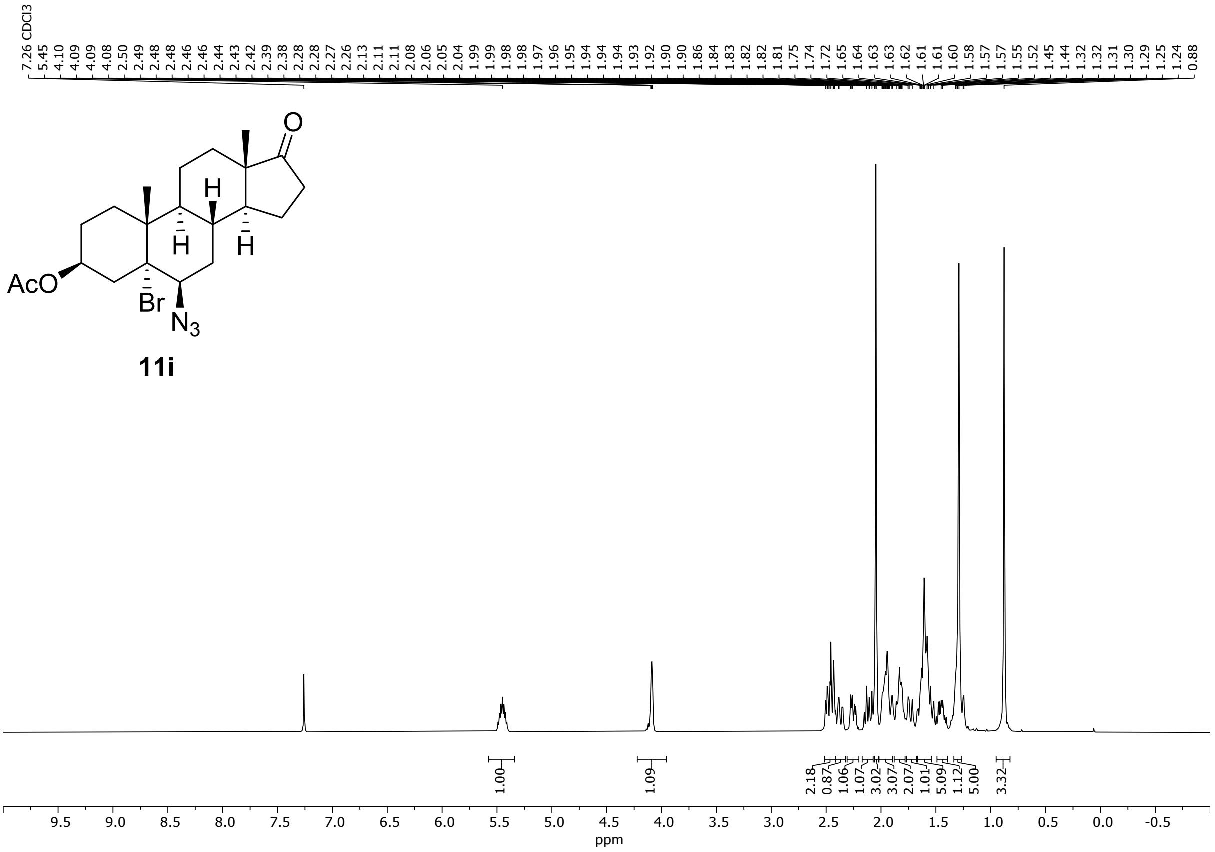

^{13}C{^{1}H} NMR (101 MHz, $CDCl_3$)

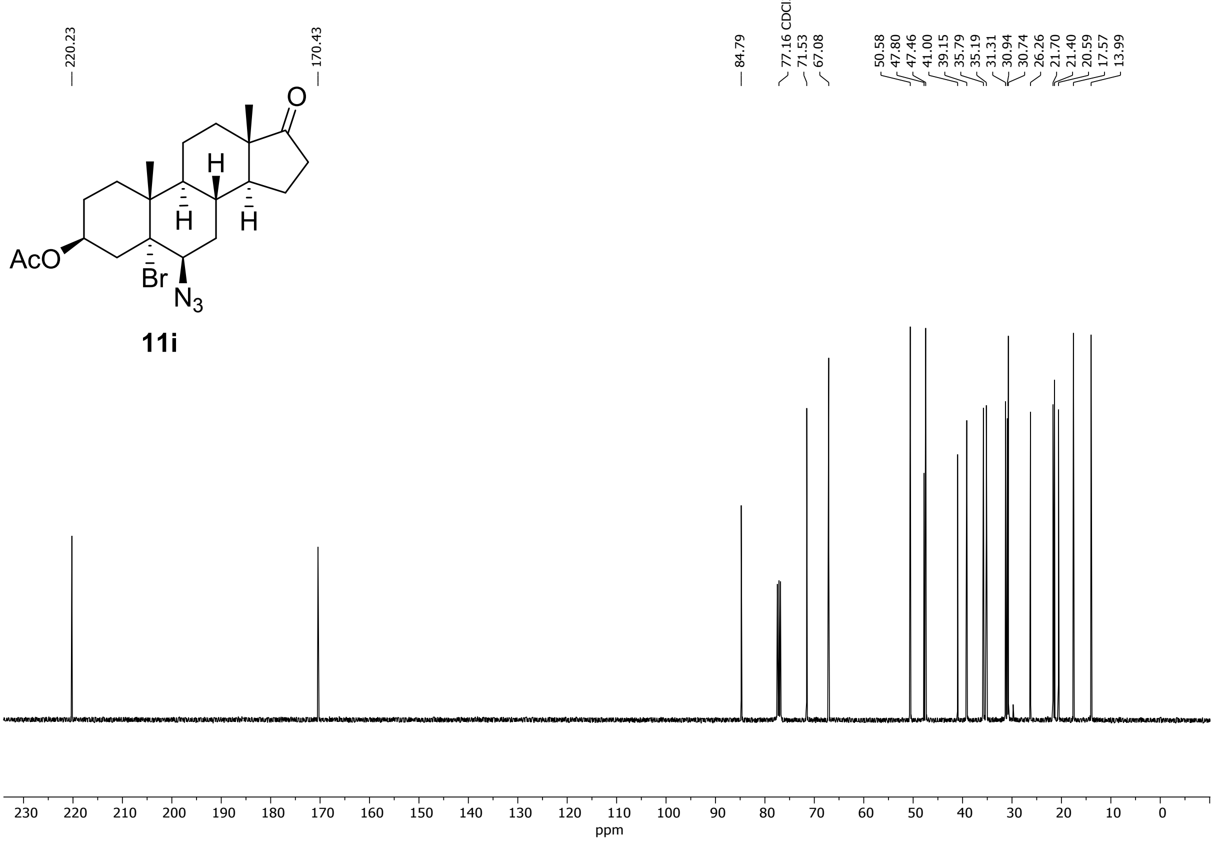

FT-IR

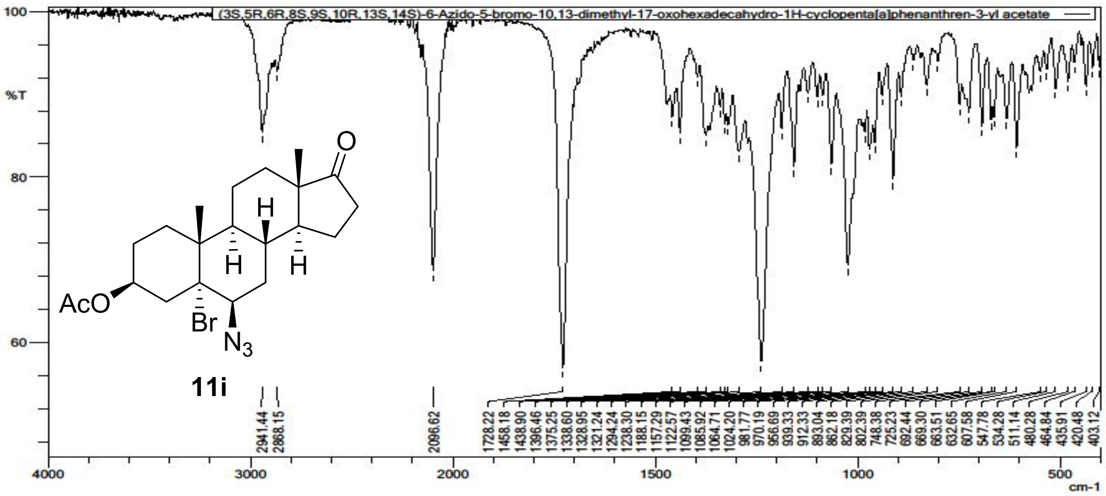

COSY ($CDCl_3$)

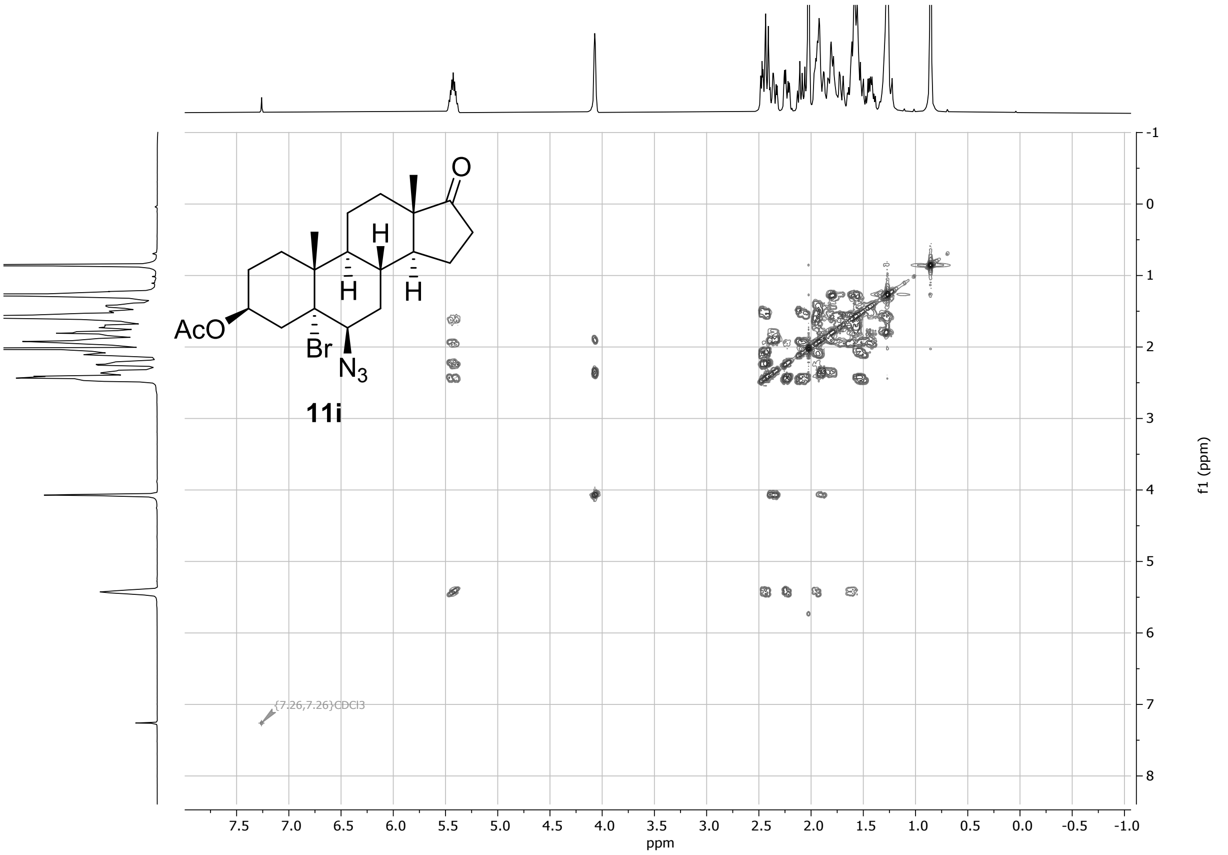

HSQC (CDCl$_3$)

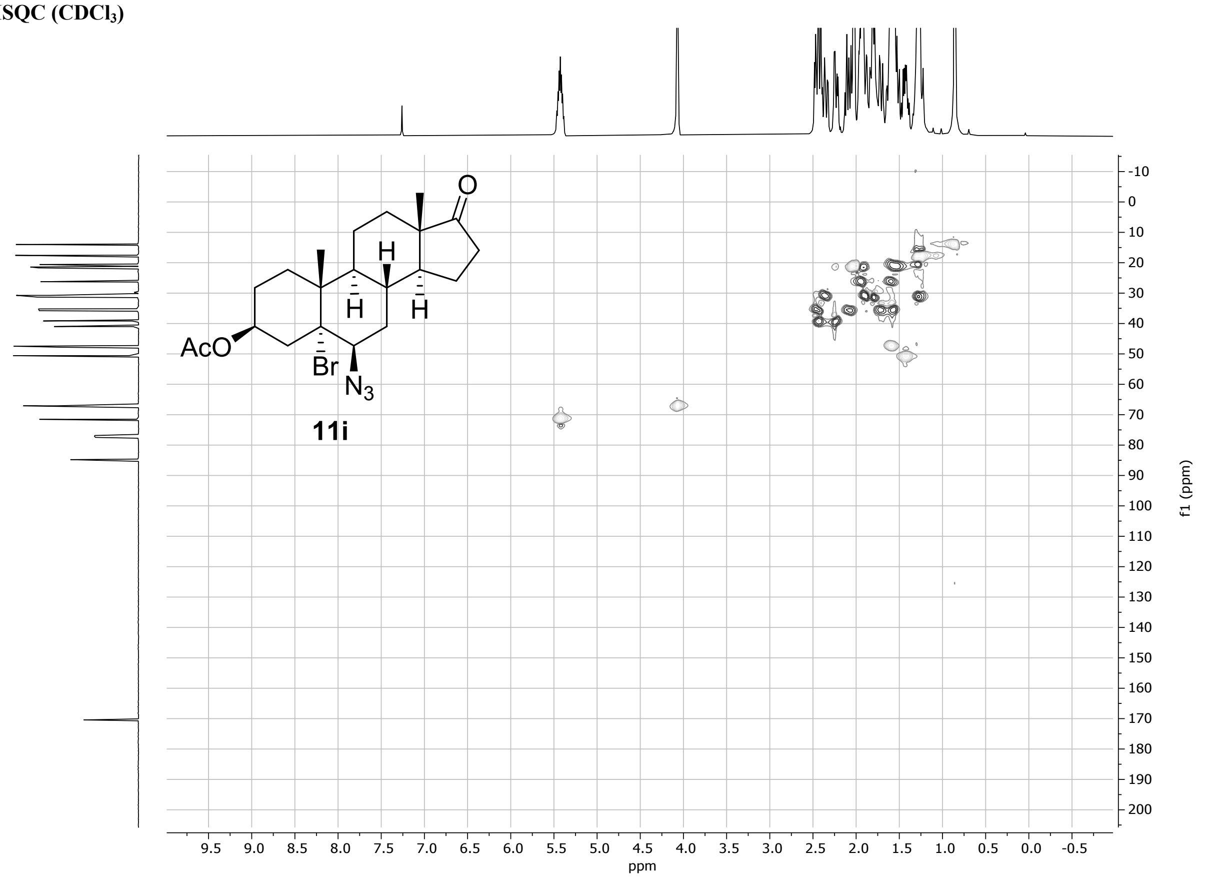

HMBC (CDCl$_3$)

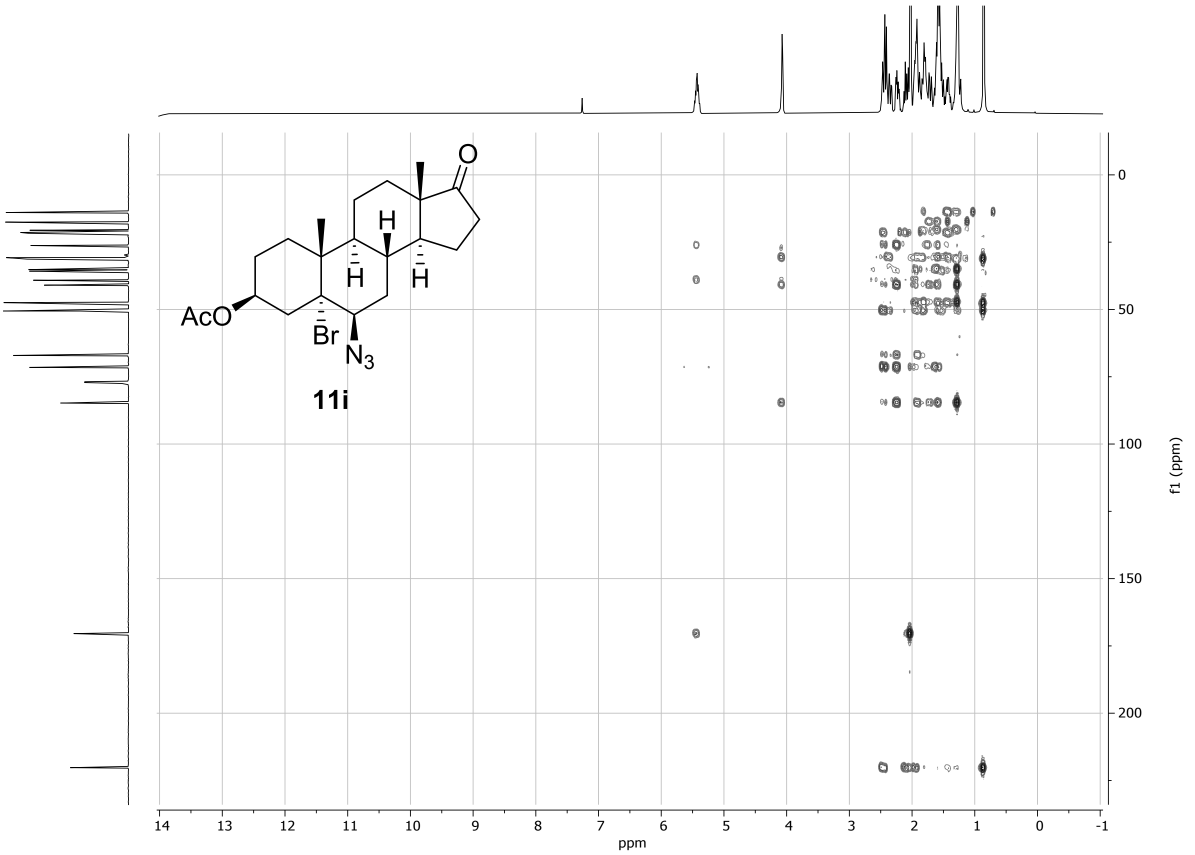

5-Azido-6-bromo-6-methylheptan-2-one (11ja)

^{1}H NMR (400 MHz, $CDCl_3$)

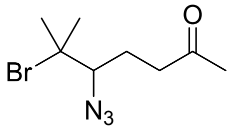

11ja

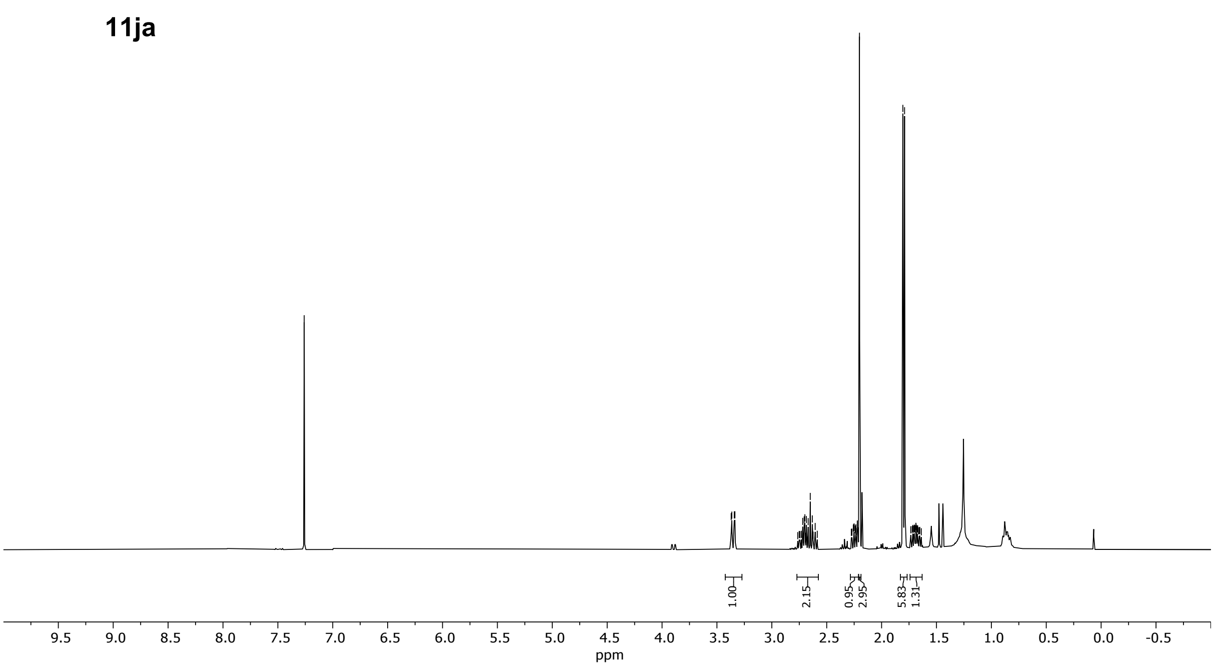

^{13}C{^{1}H} NMR (101 MHz, $CDCl_3$)

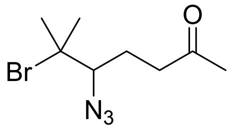

11ja

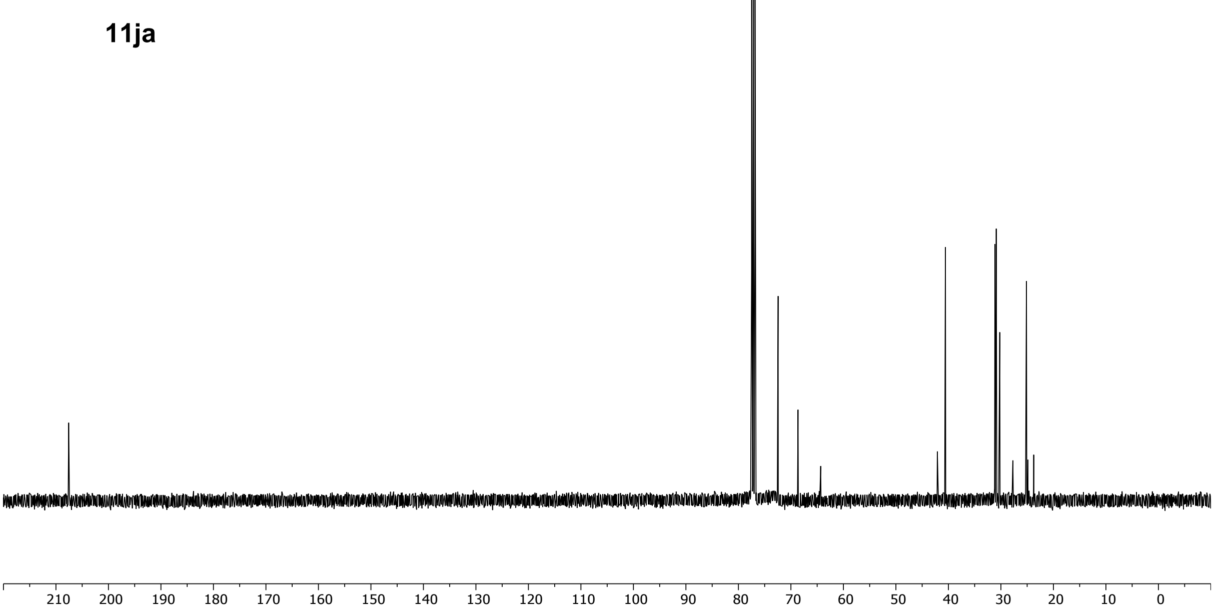

FT-IR

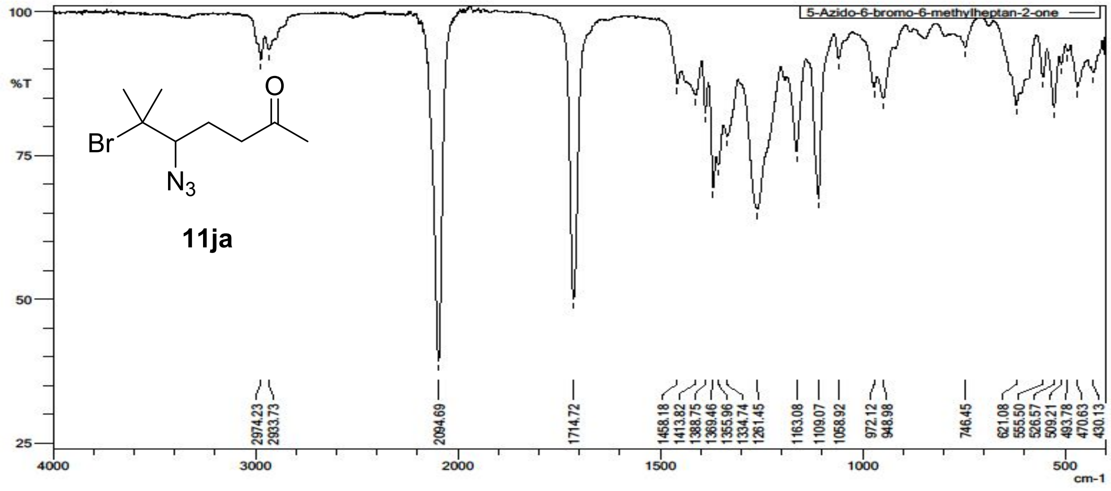

6-Azido-5-bromo-6-methylheptan-2-one (11jb)

^{1}H NMR (400 MHz, $CDCl_3$)

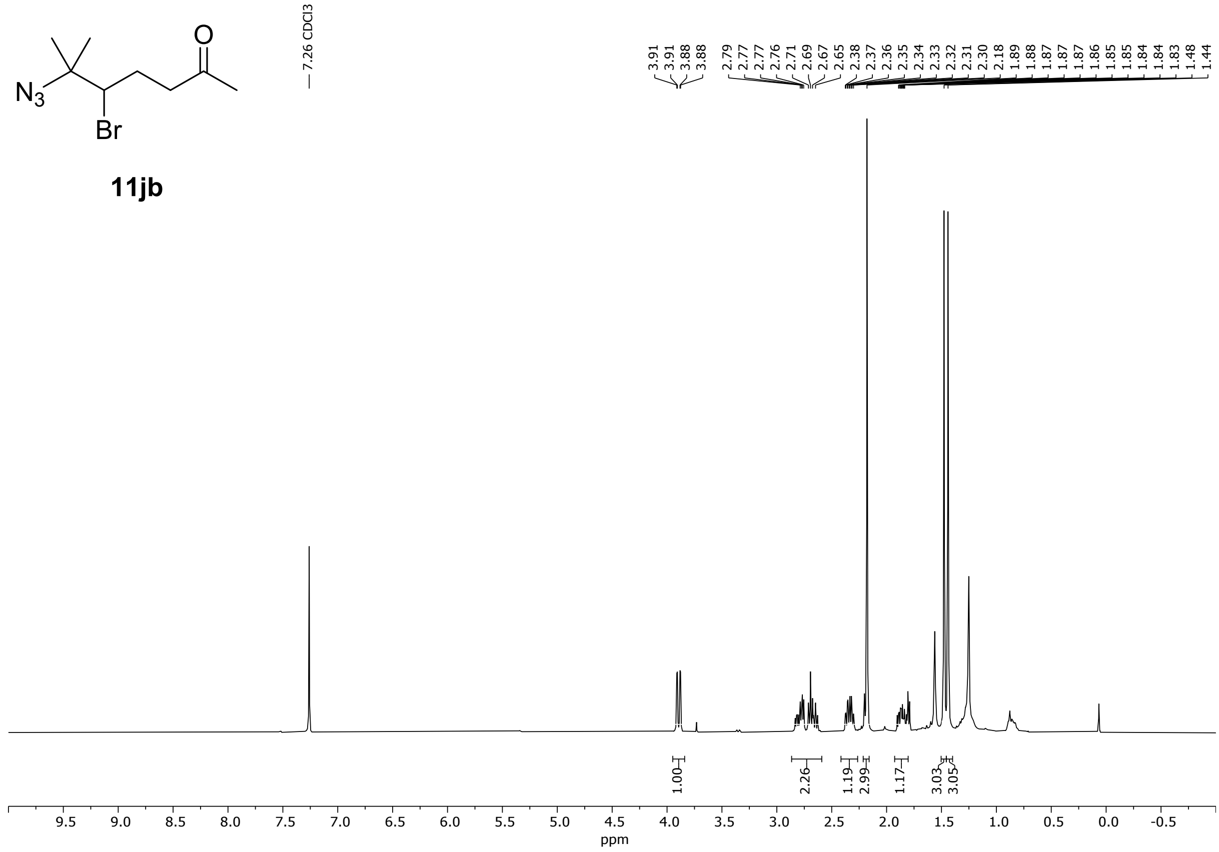

^{13}C{^{1}H} NMR (101 MHz, $CDCl_3$)

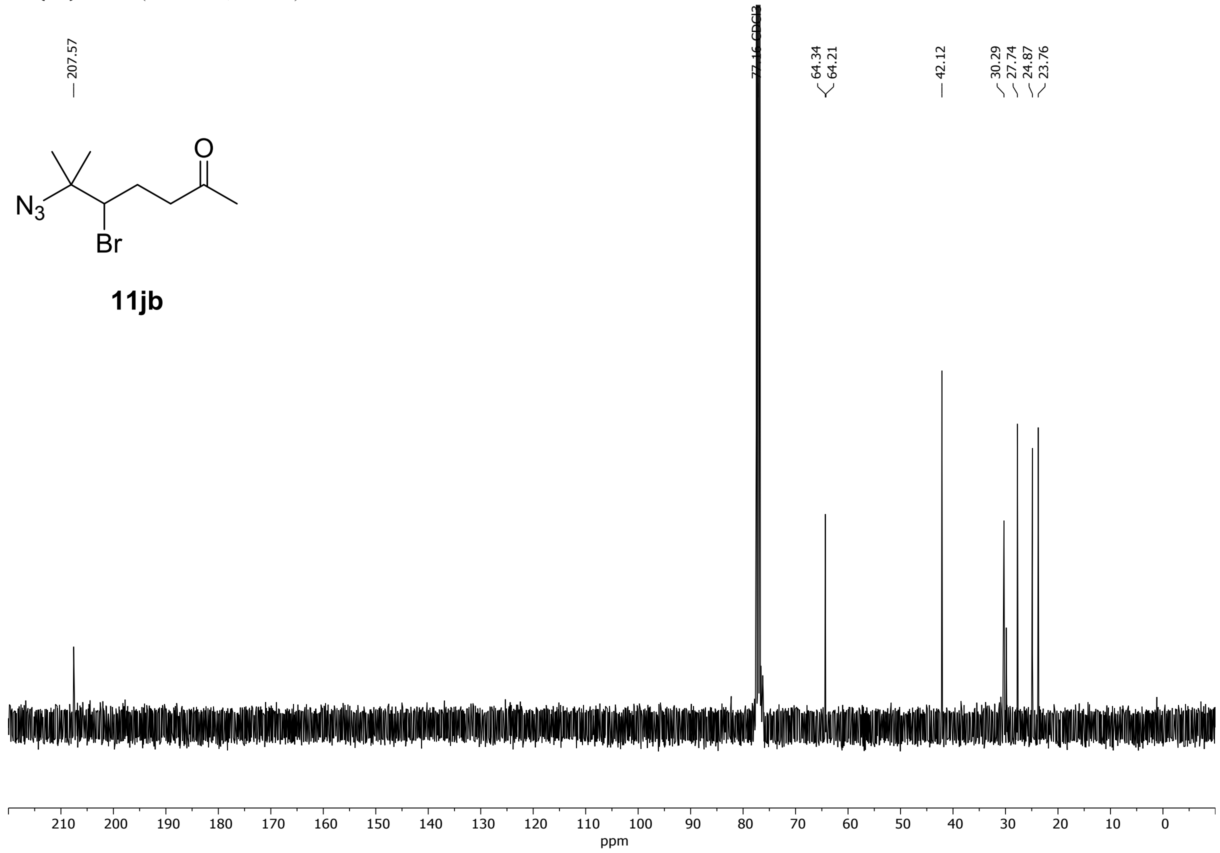

FT-IR

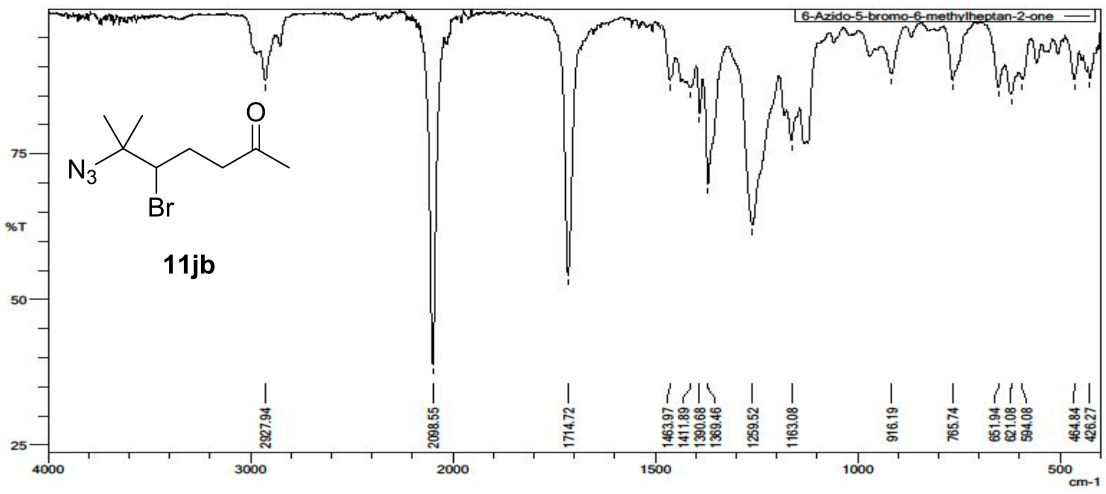

(4-Azido-3-bromobutyl)benzene (12)

^{1}H NMR (400 MHz, $CDCl_3$)

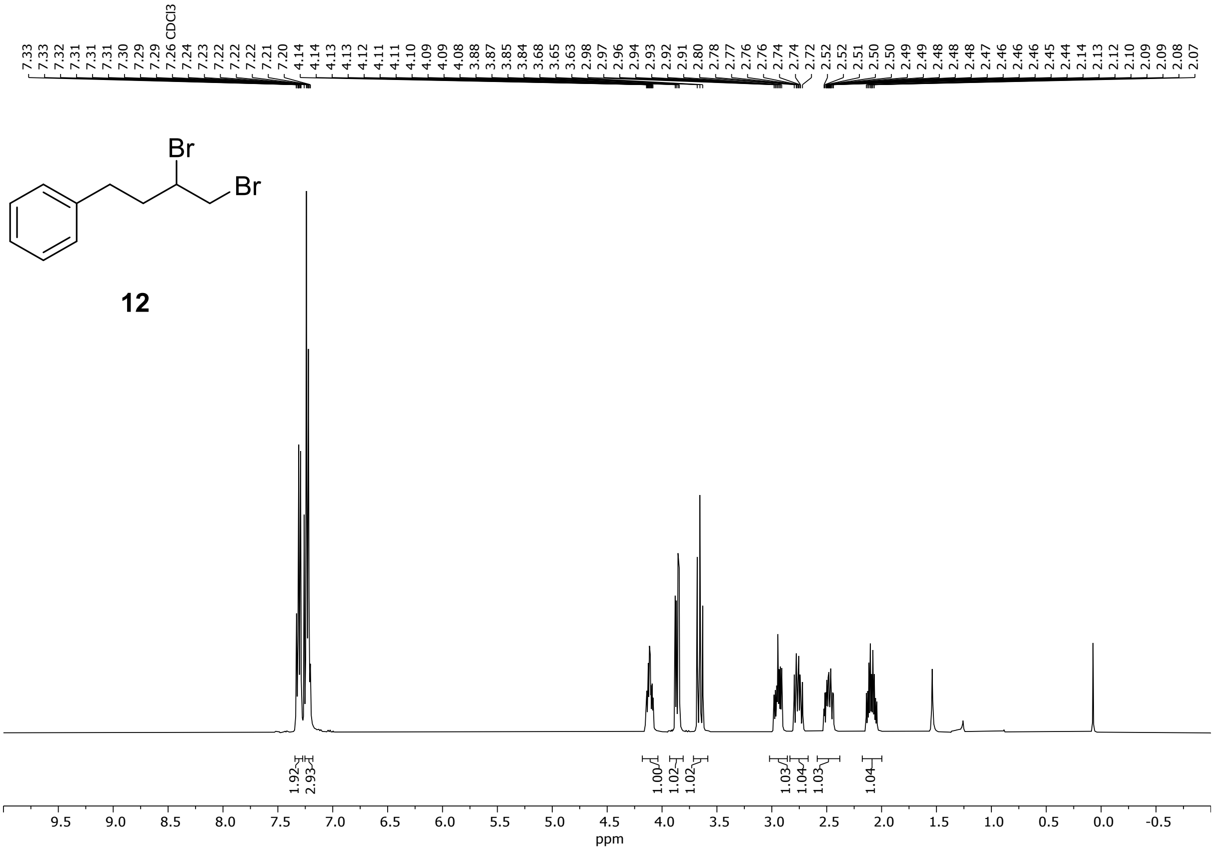

^{13}C{^{1}H} NMR (101 MHz, $CDCl_3$)

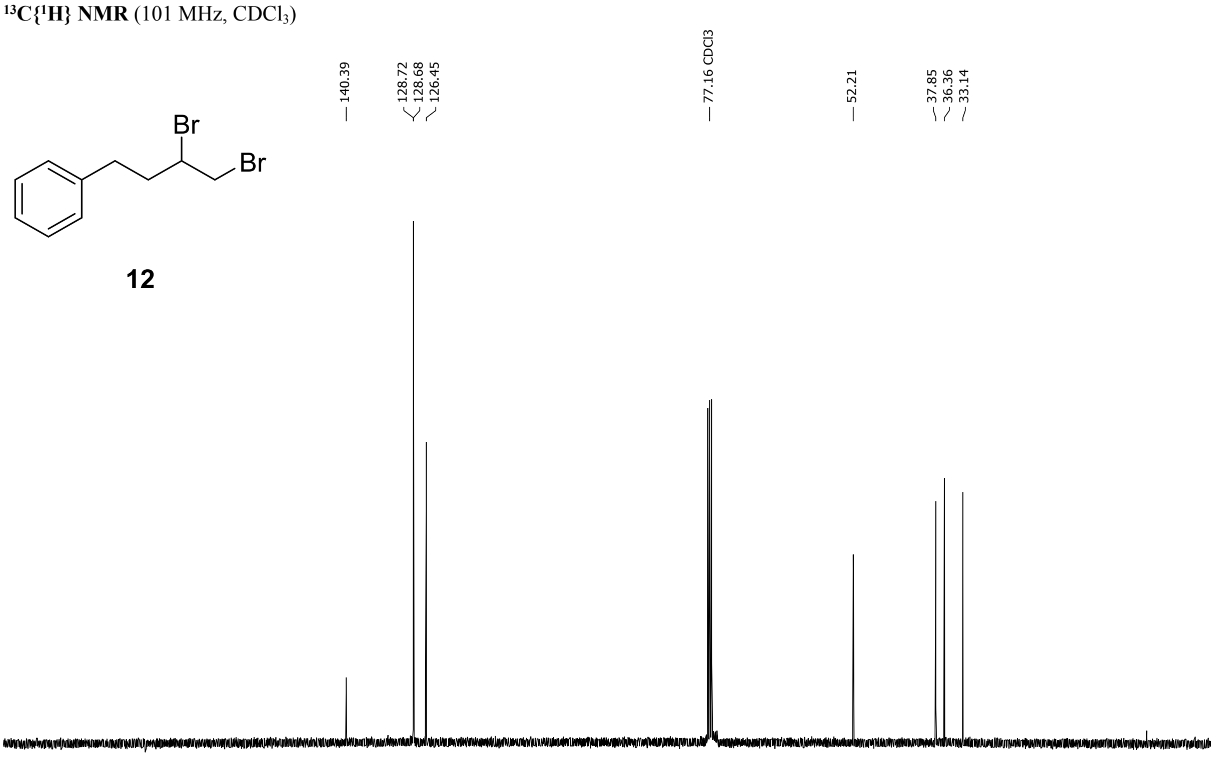

1,2-Dibromooctane (13)

^{1}H NMR (400 MHz, $CDCl_3$)

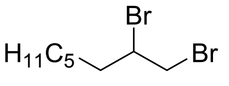

13

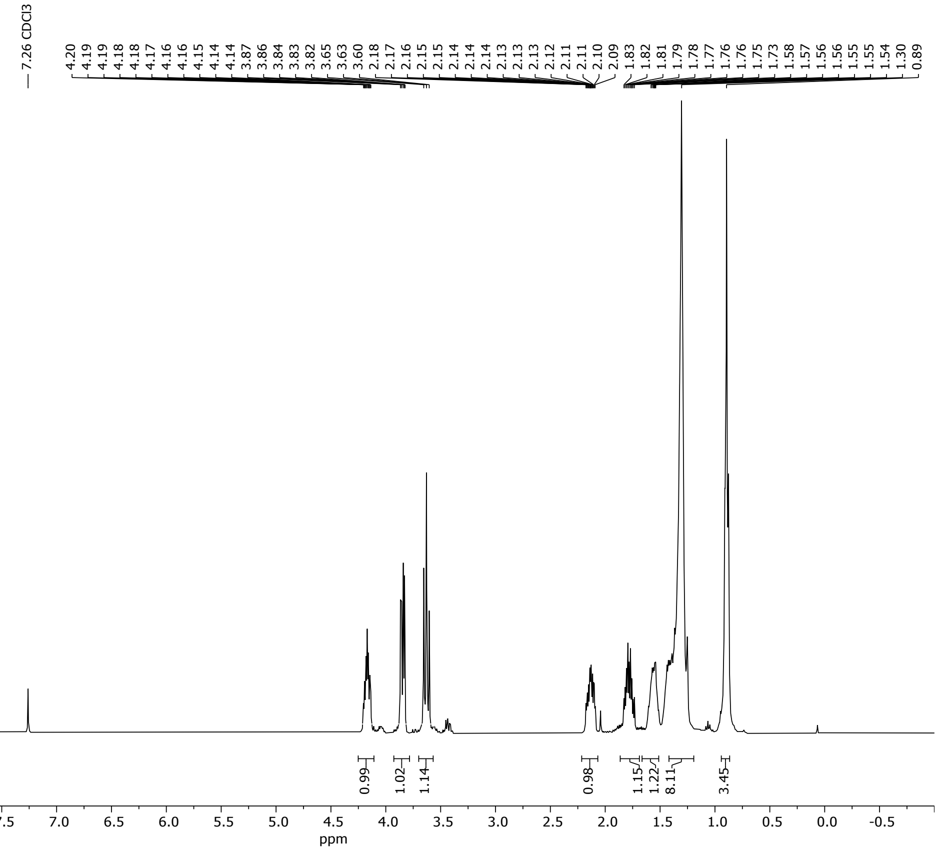

^{13}C{^{1}H} NMR (101 MHz, $CDCl_3$)

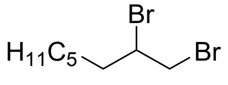

13

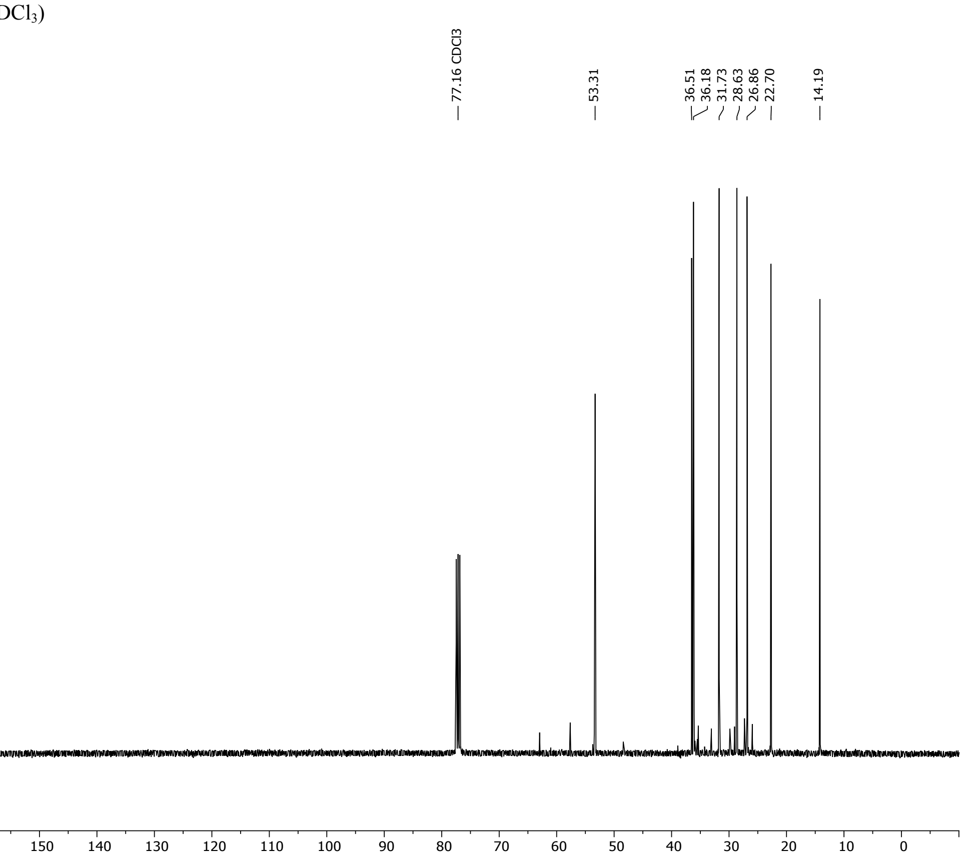

1-(((1*R**,2*R**)-2-azido-2,3-dihydro-1*H*-inden-1-yl)oxy)-2,2,6,6-tetramethylpiperidine (14)

^{1}H NMR (400 MHz, $CDCl_3$)

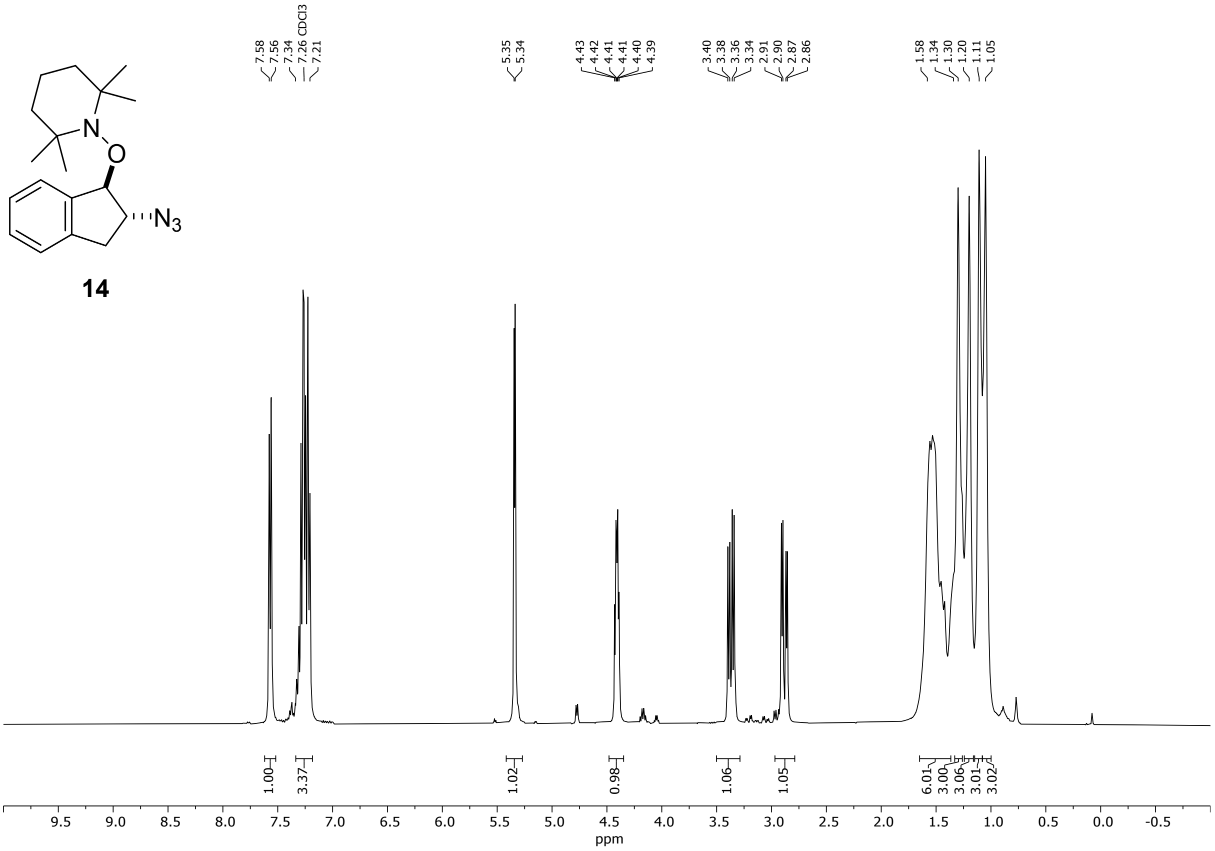

^{13}C{^{1}H} NMR (101 MHz, $CDCl_3$)

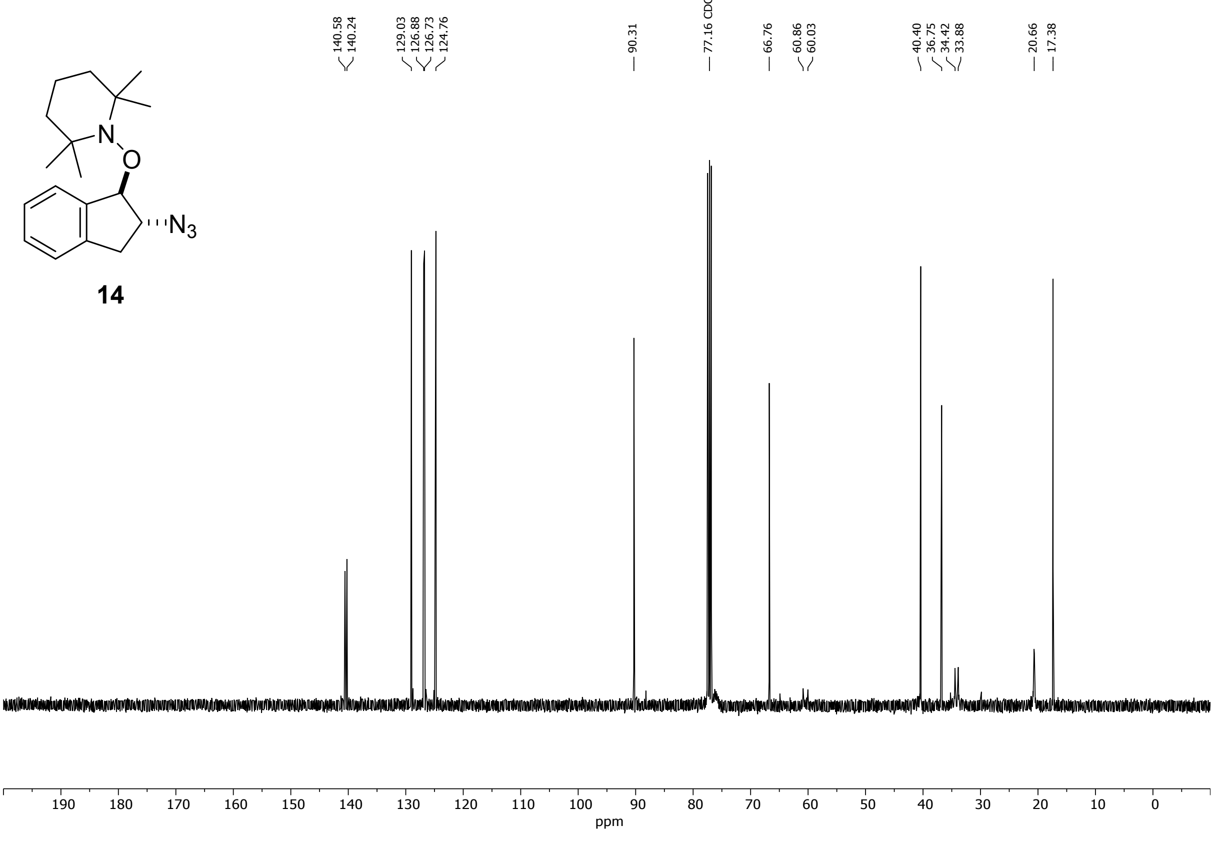

FT-IR

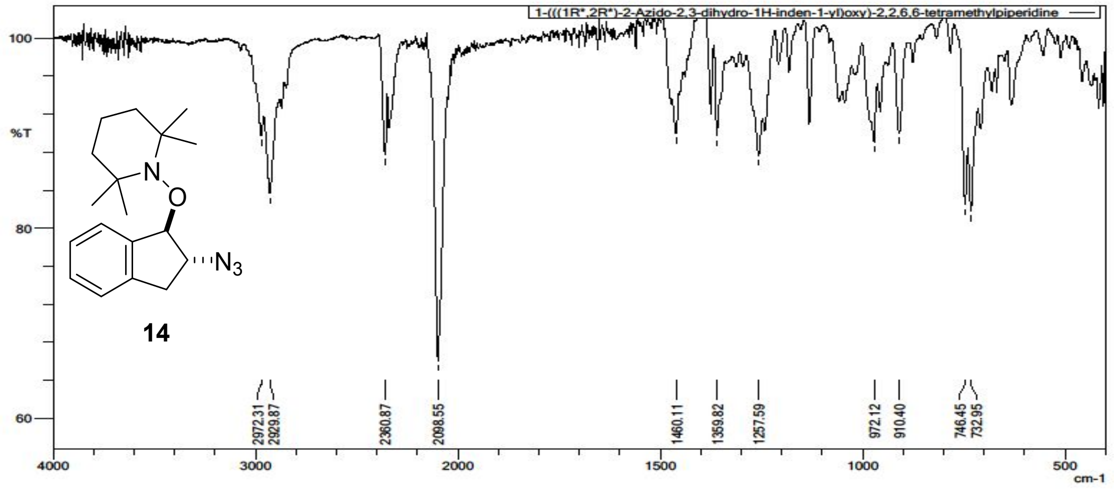

(1*S**,2*S**)-1,2-Dibromo-2,3-dihydro-1*H*-indene (S1)

¹H NMR (400 MHz, $CDCl_3$)

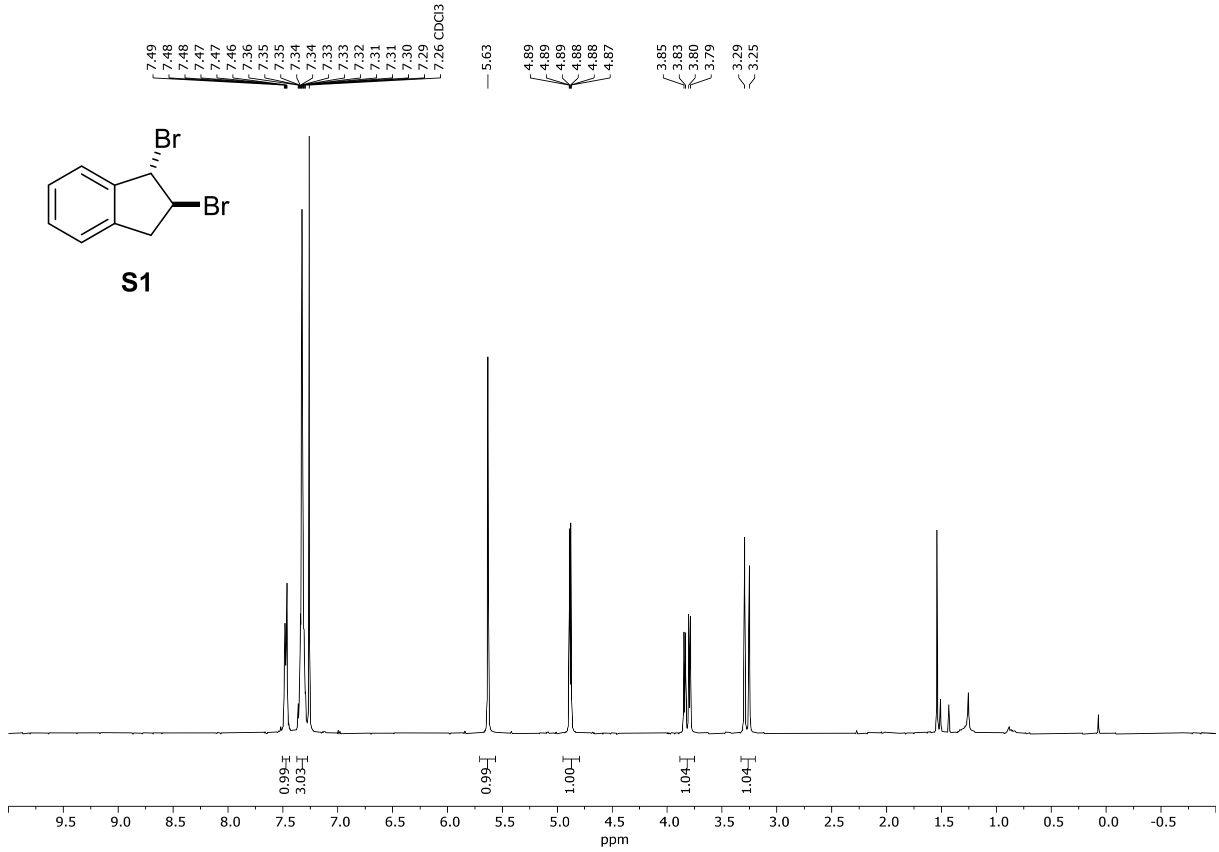

$^{13}C\{^1H\}$ NMR (101 MHz, $CDCl_3$)

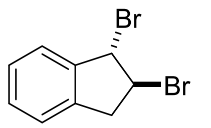

S1

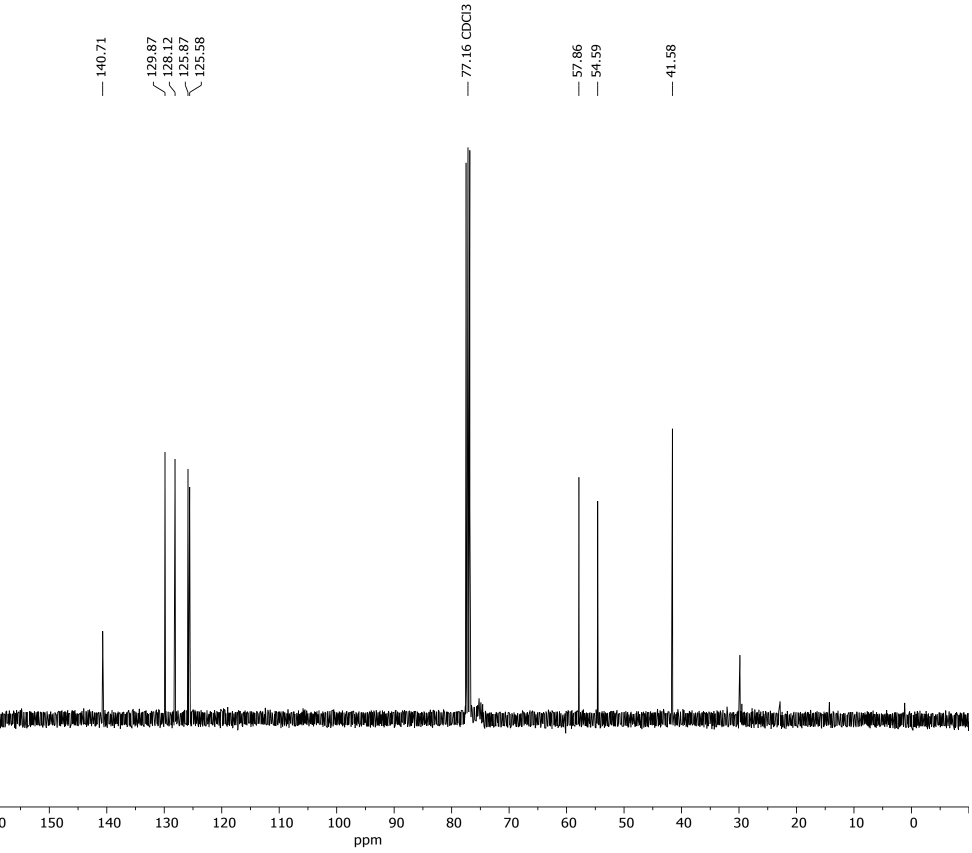

3-(Azidomethyl)-4-(bromomethyl)-3-methyl-1-tosylpyrrolidine (16)

^1H NMR (400 MHz, $CDCl_3$)

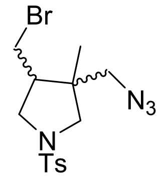

16

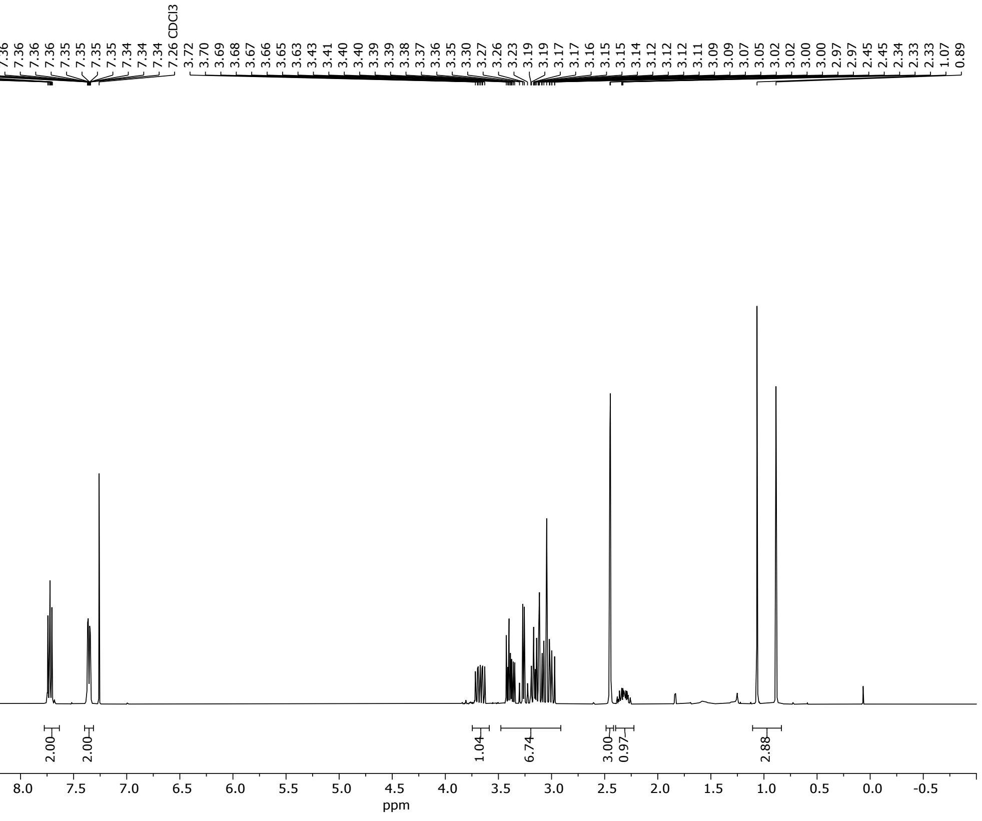

$^{13}C\{^1H\}$ NMR (101 MHz, $CDCl_3$)

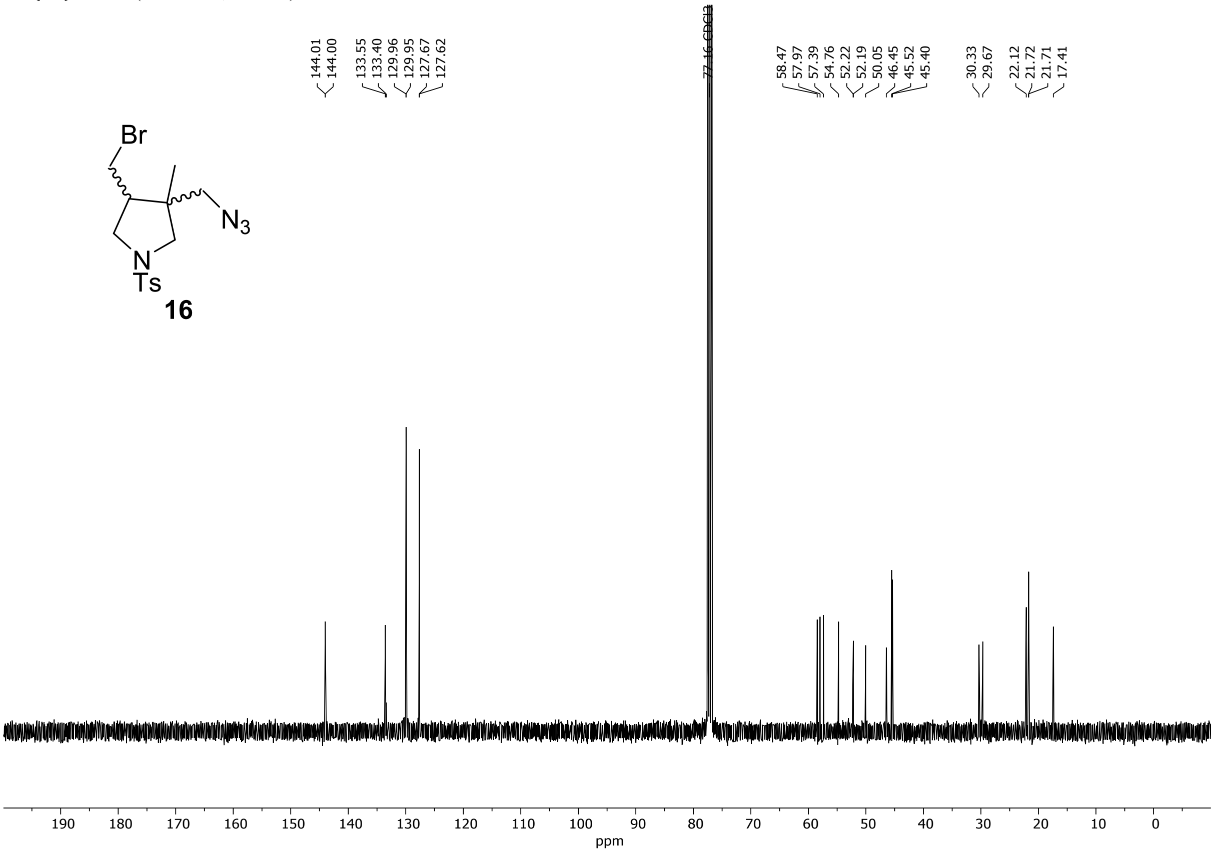

FT-IR

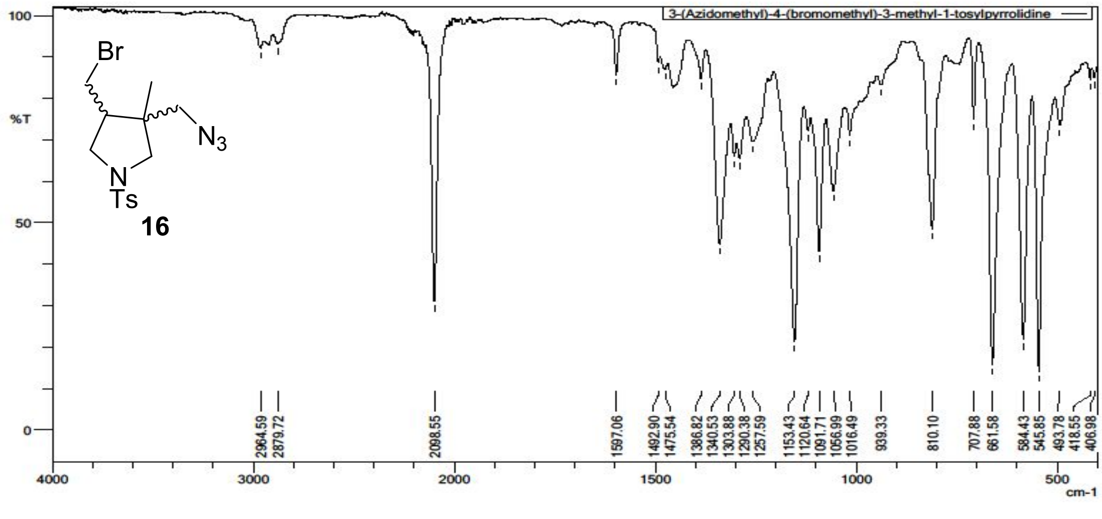

Dibenzyl (*E*)-2-(4-azidobut-2-en-1-yl)-2-bromomalonate (18)

^{1}H NMR (400 MHz, $CDCl_3$)

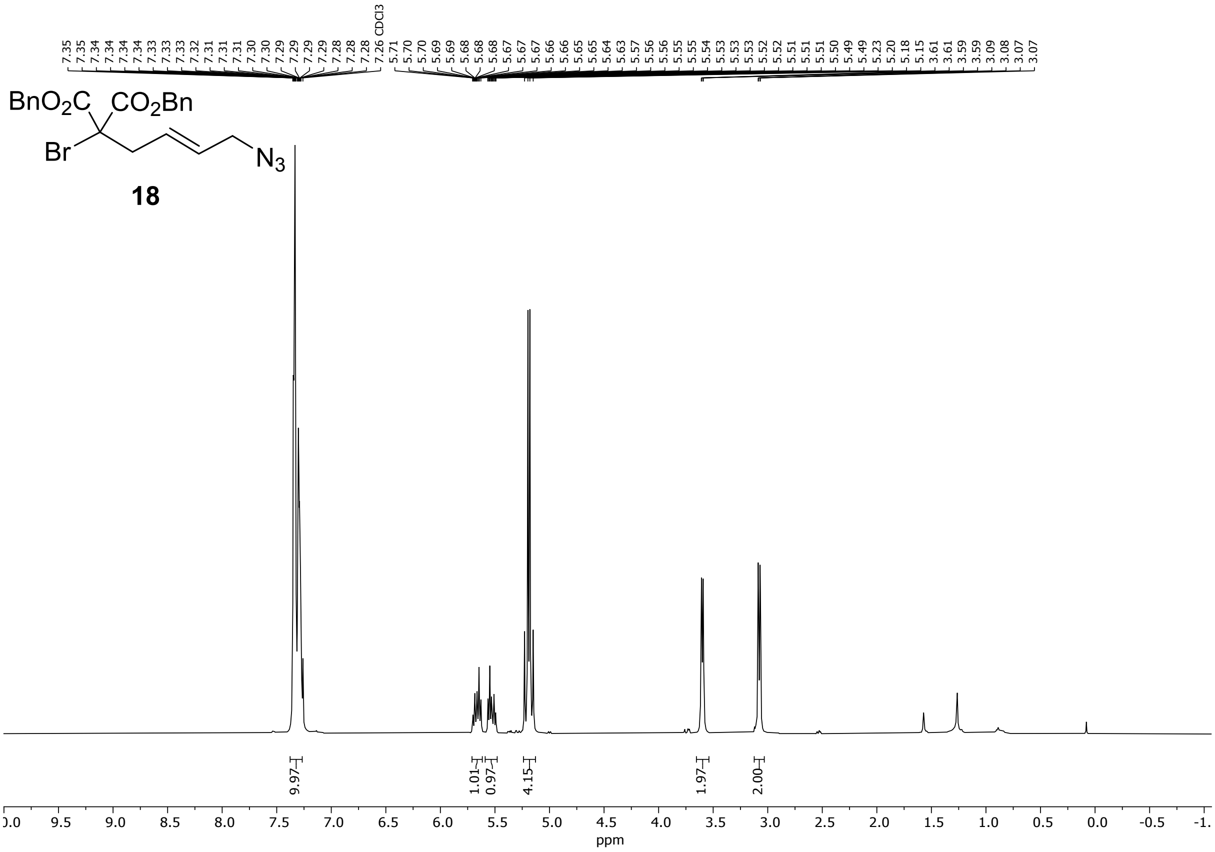

^{13}C{^{1}H} NMR (101 MHz, $CDCl_3$)

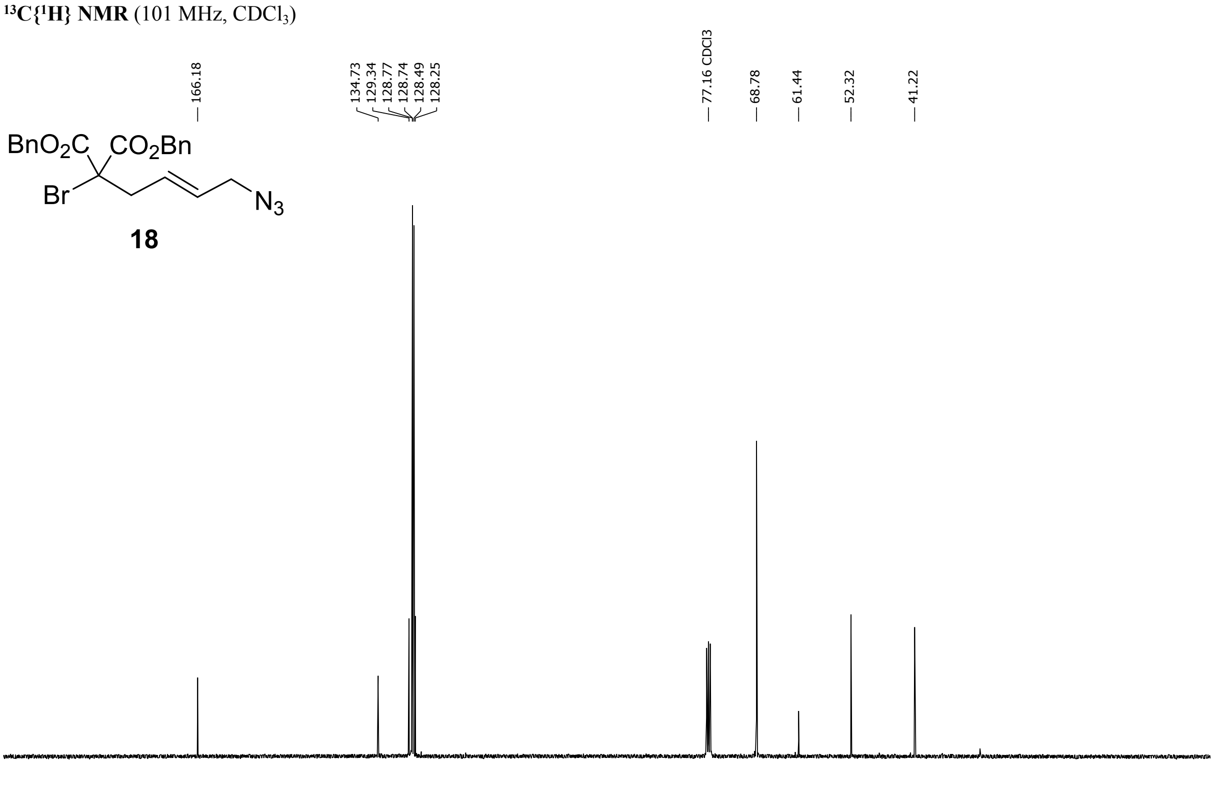

FT-IR

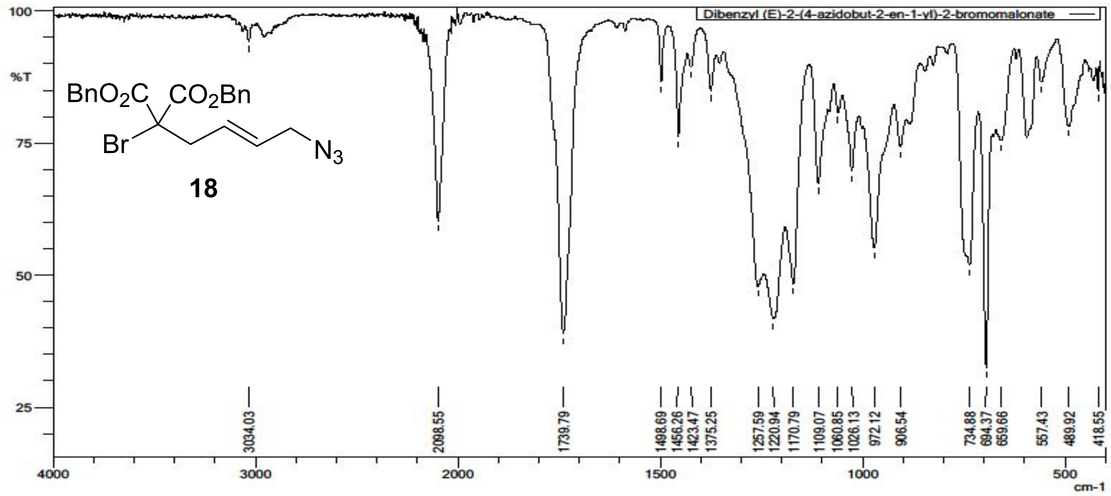

Benzophenone (20a)

^{1}H NMR (400 MHz, $CDCl_3$)

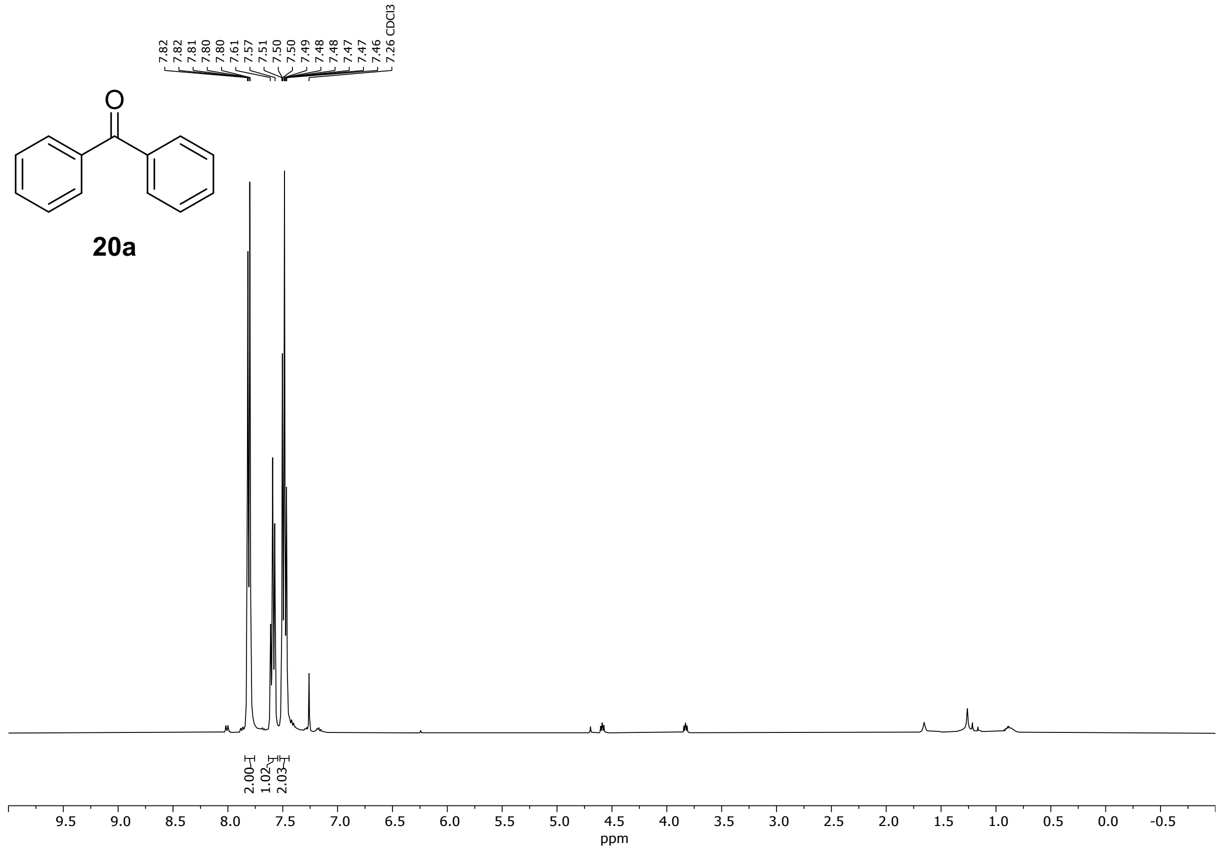

$^{13}C\{^1H\}$ NMR (101 MHz, $CDCl_3$)

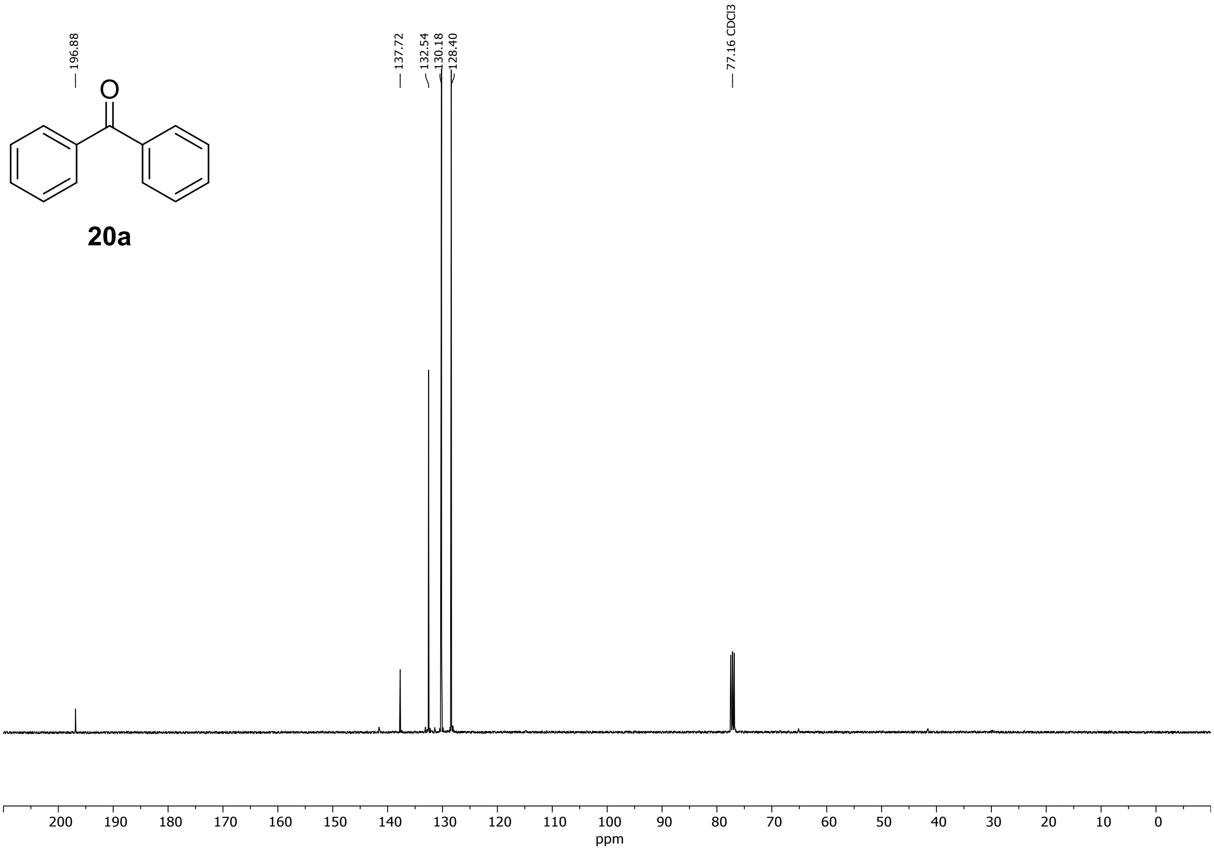

20a

(*1R*,4*S*)-1,3,3-Trimethylbicyclo[2.2.1]heptan-2-one (20b)

^1H NMR (400 MHz, $CDCl_3$)

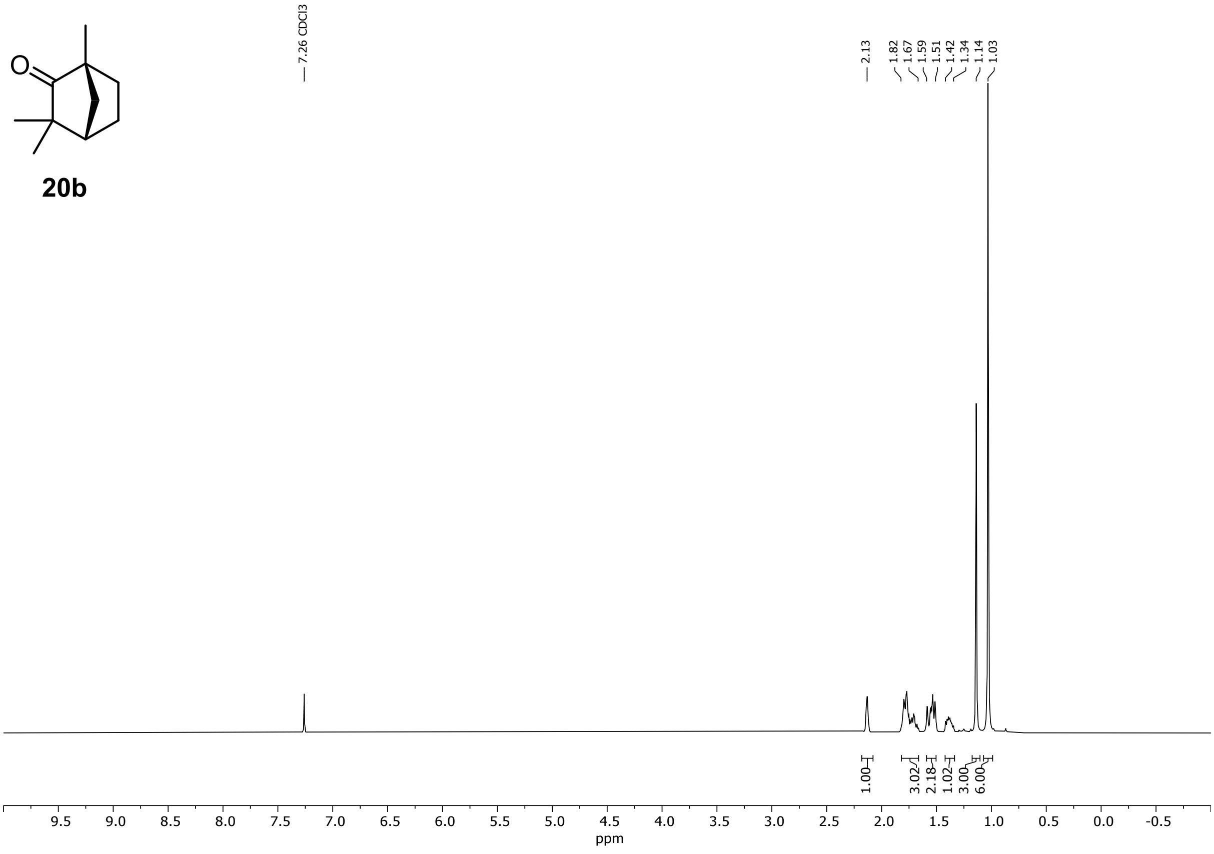

20b

$^{13}C\{^{1}H\}$ **NMR** (101 MHz, $CDCl_3$)

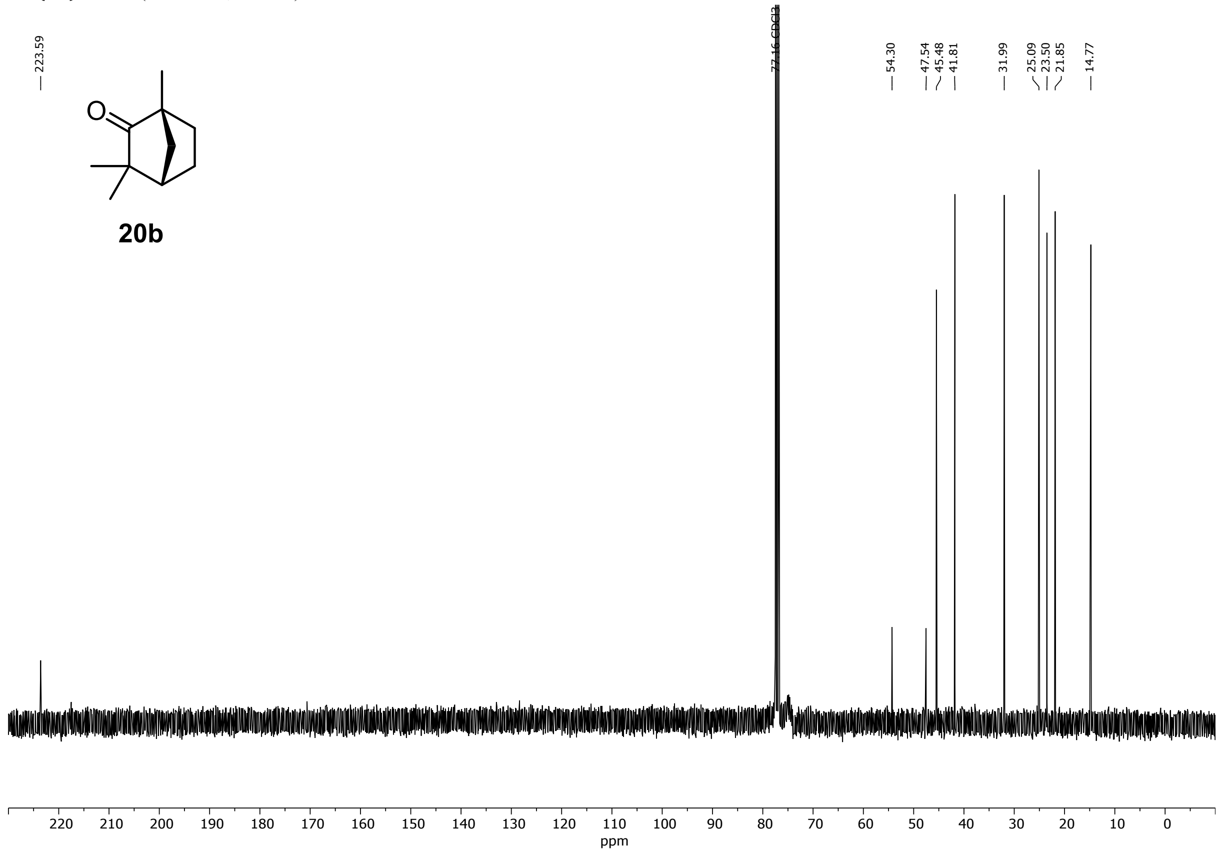

(2*S*,5*R*)-2-Isopropyl-5-methylcyclohexan-1-one (20c)

^{1}H **NMR** (400 MHz, $CDCl_3$)

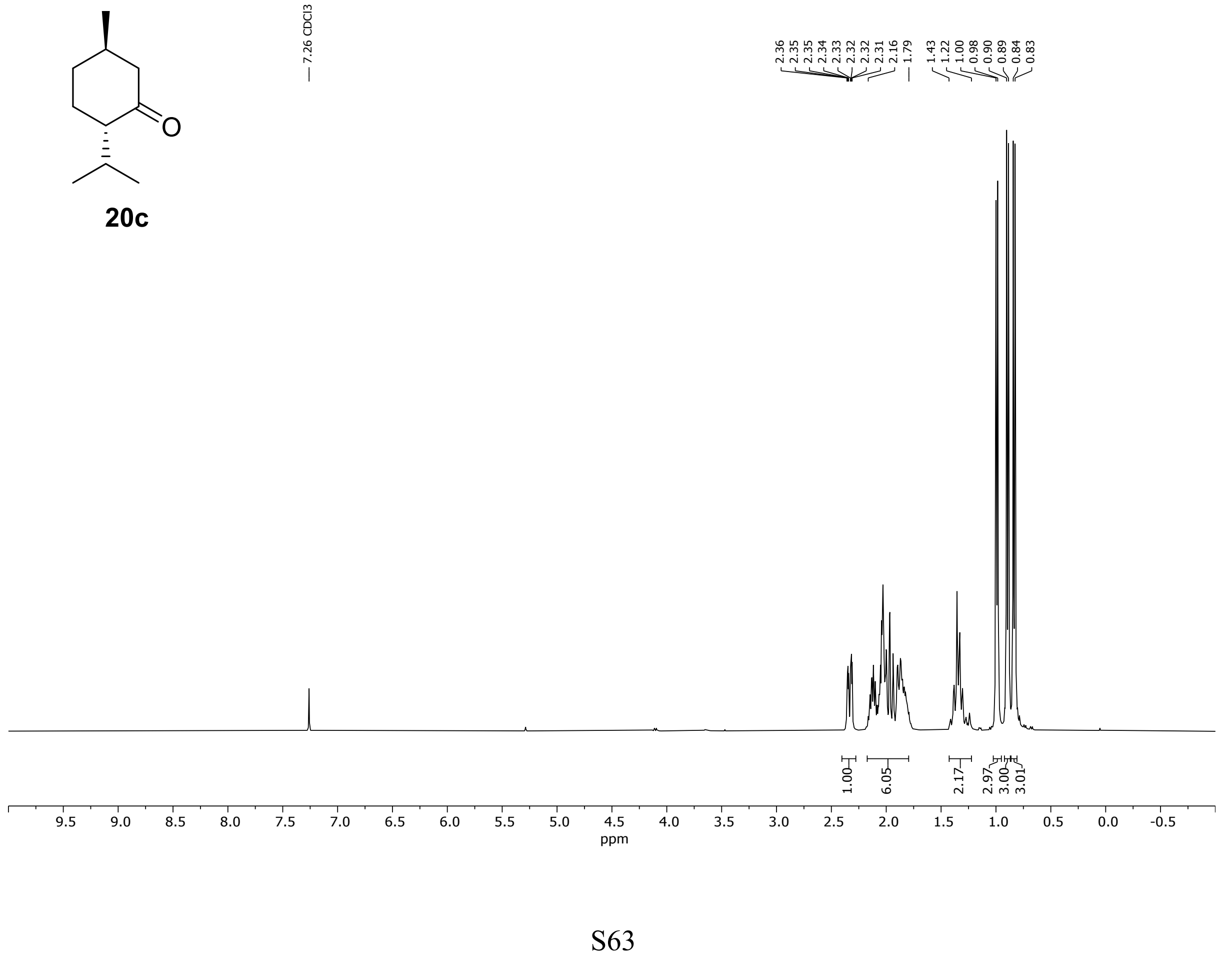

$^{13}C\{^{1}H\}$ NMR (101 MHz, $CDCl_3$)

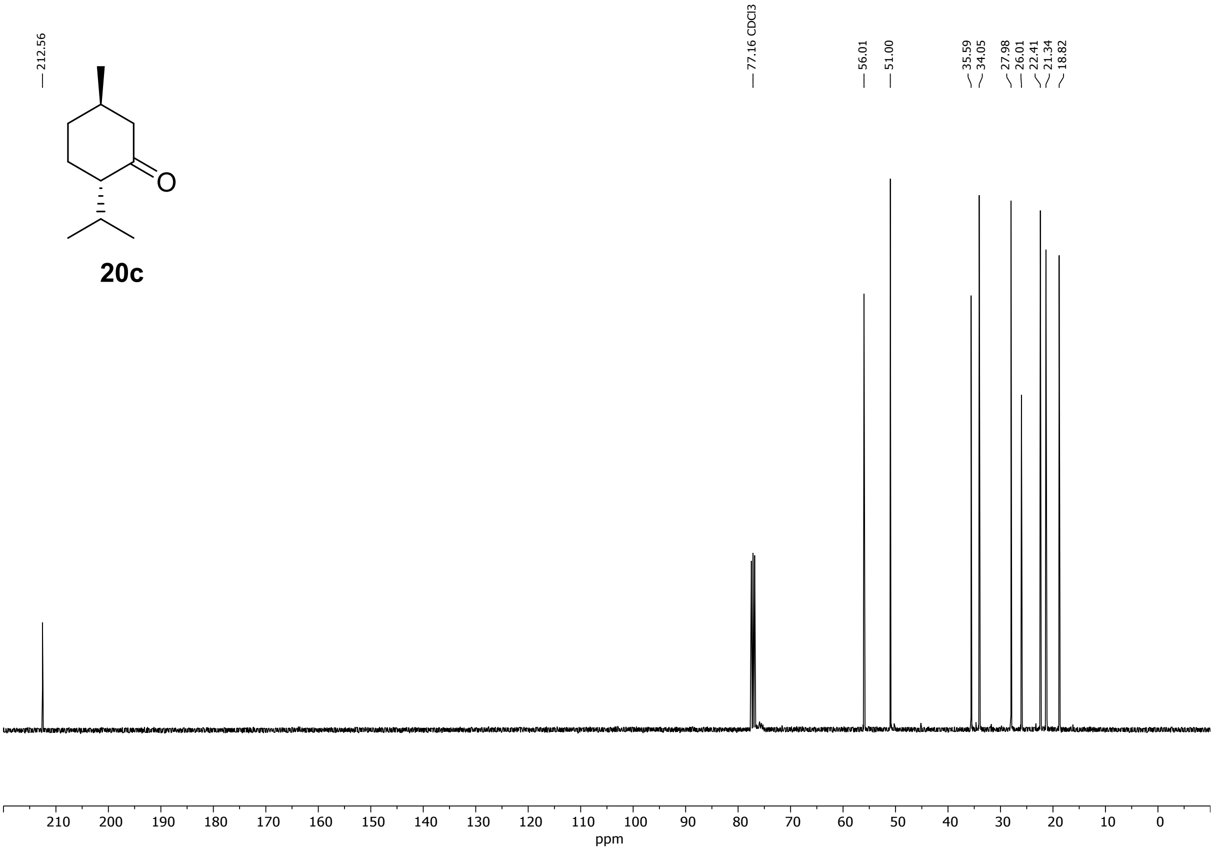

1-Hydroxyoctan-2-one (22)

^{1}H NMR (400 MHz, $CDCl_3$)

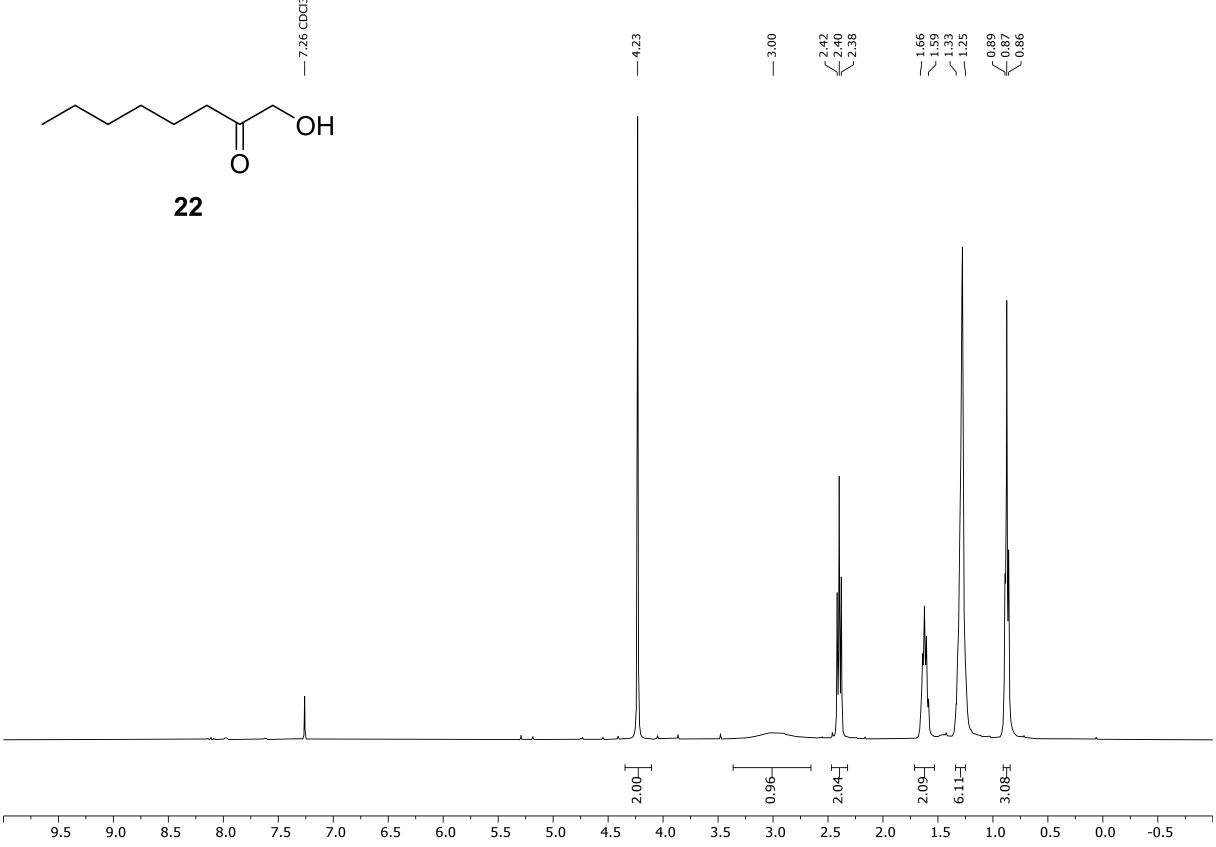

$^{13}C\{^1H\}$ **NMR** (101 MHz, $CDCl_3$)

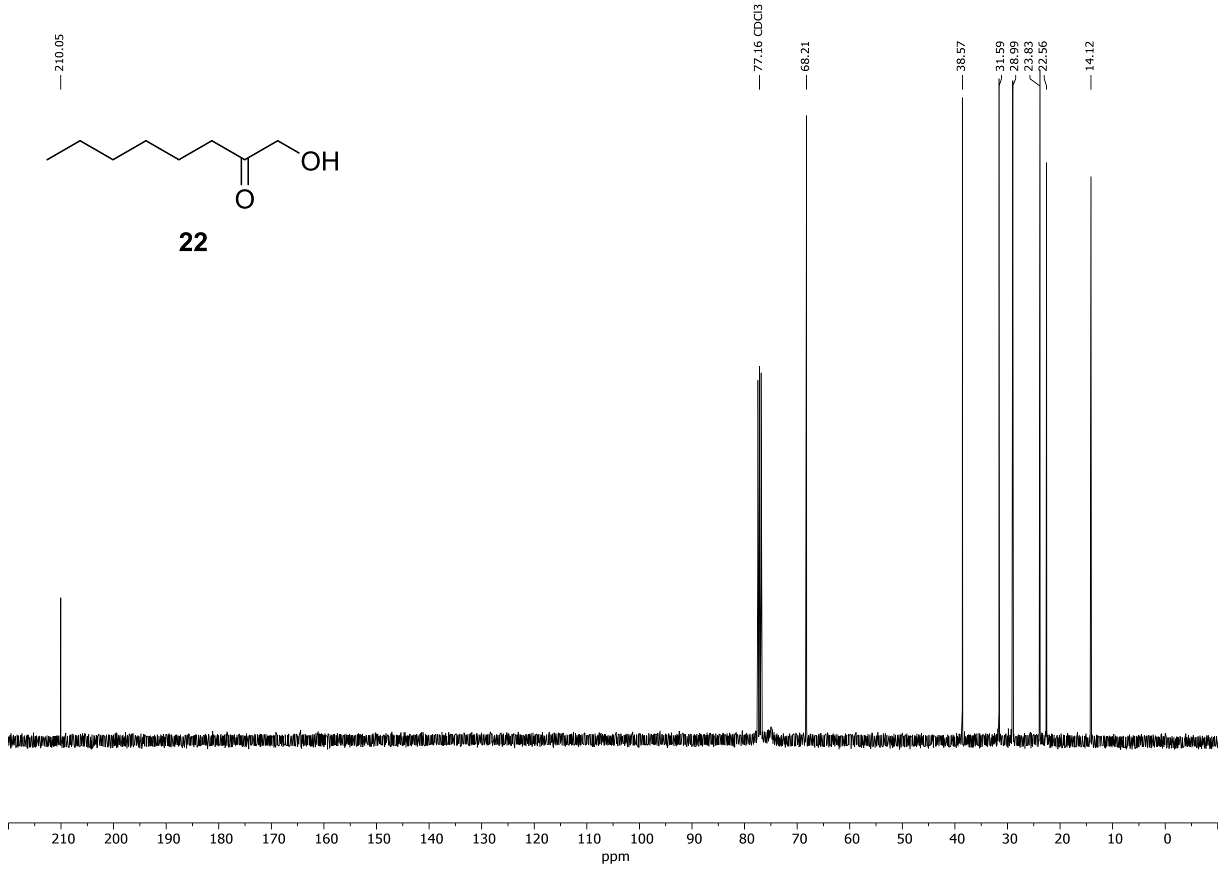

3-(Hydroxymethyl)heptan-4-one (24)

^{1}**H NMR** (400 MHz, $CDCl_3$)

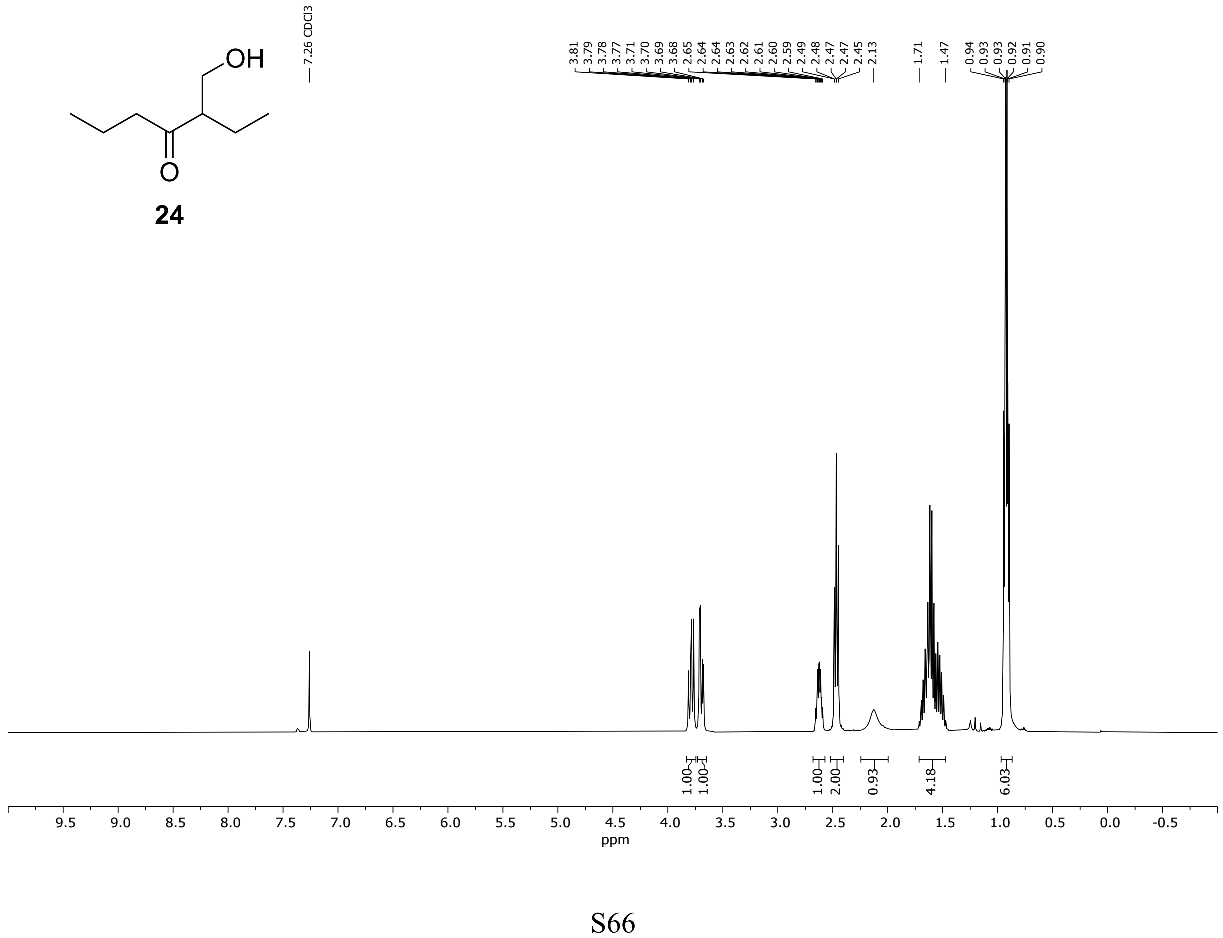

$^{13}C\{^1H\}$ NMR (101 MHz, $CDCl_3$)

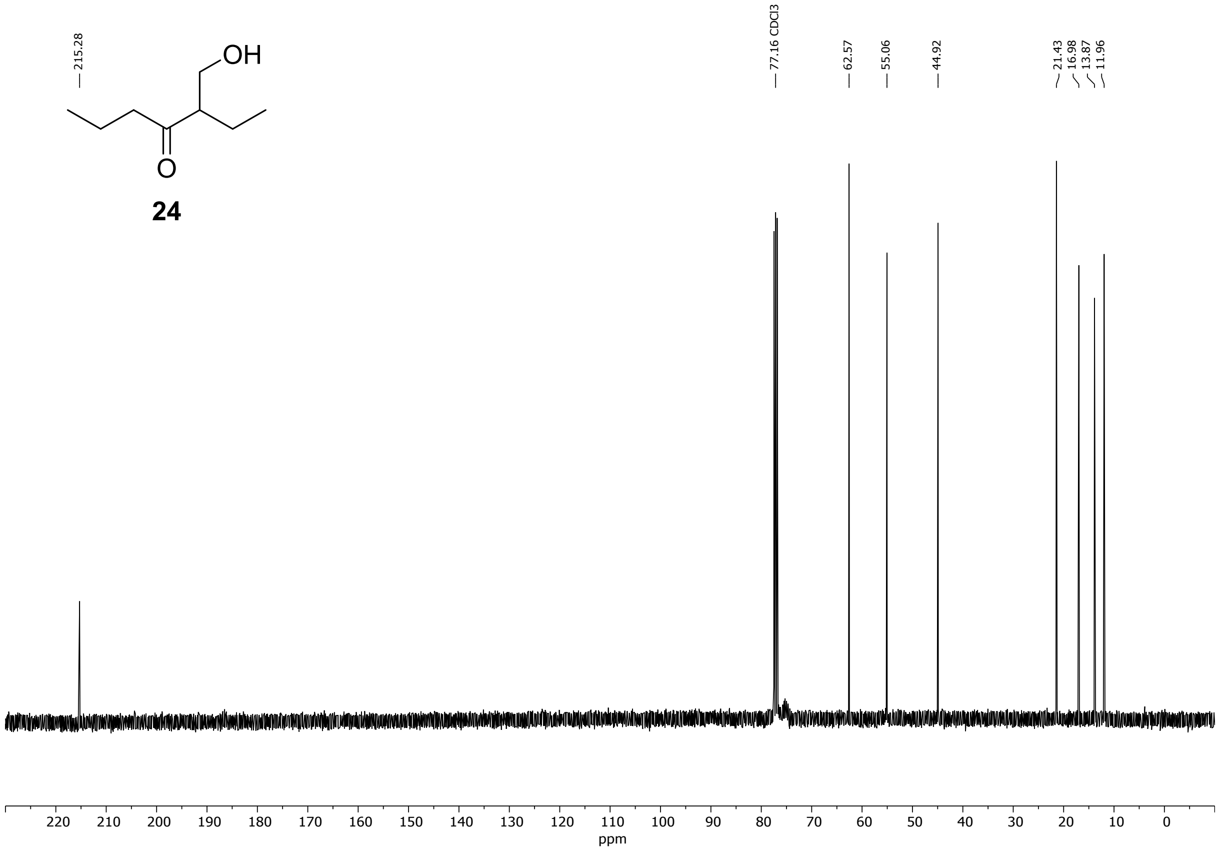

2-Hydroxy-1-phenylethan-1-one (26)

^1H NMR (400 MHz, $CDCl_3$)

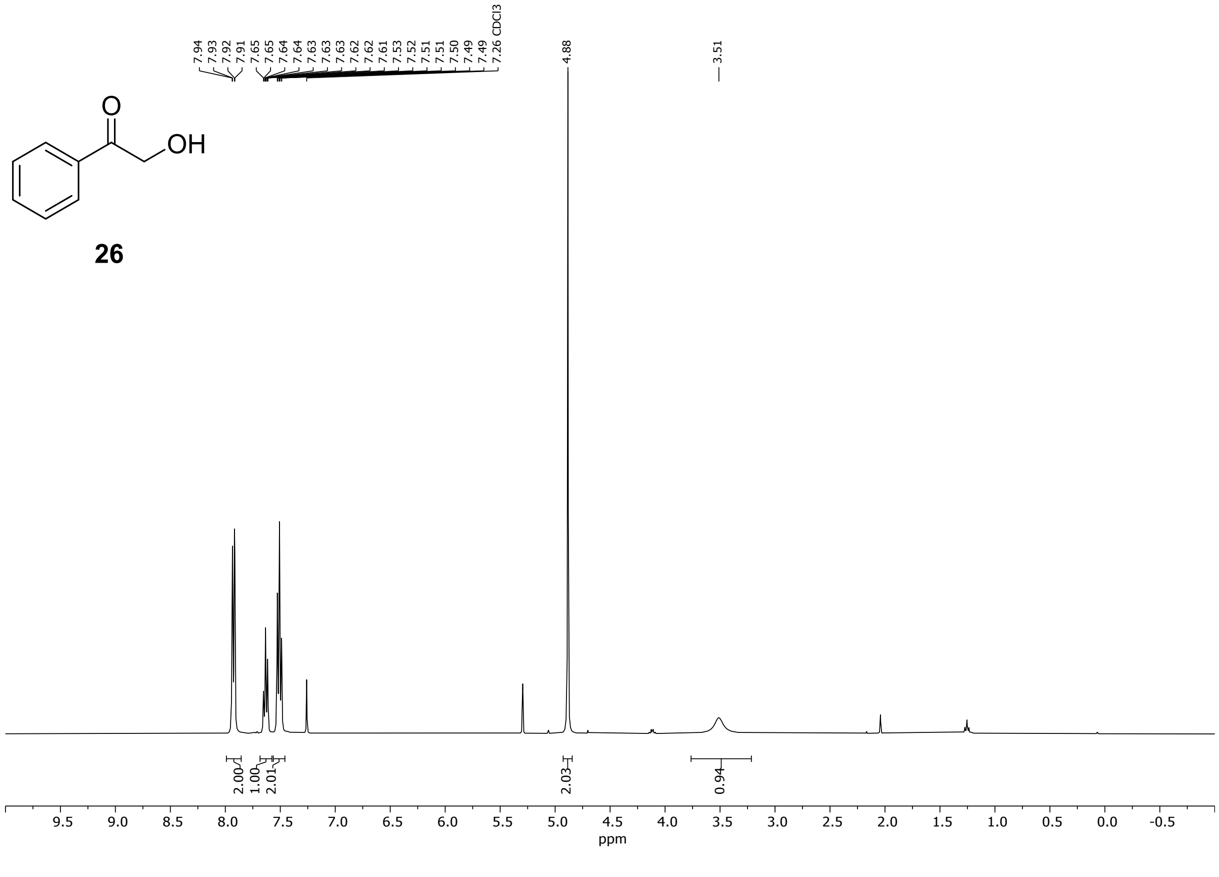

$^{13}C\{^1H\}$ **NMR** (101 MHz, $CDCl_3$)

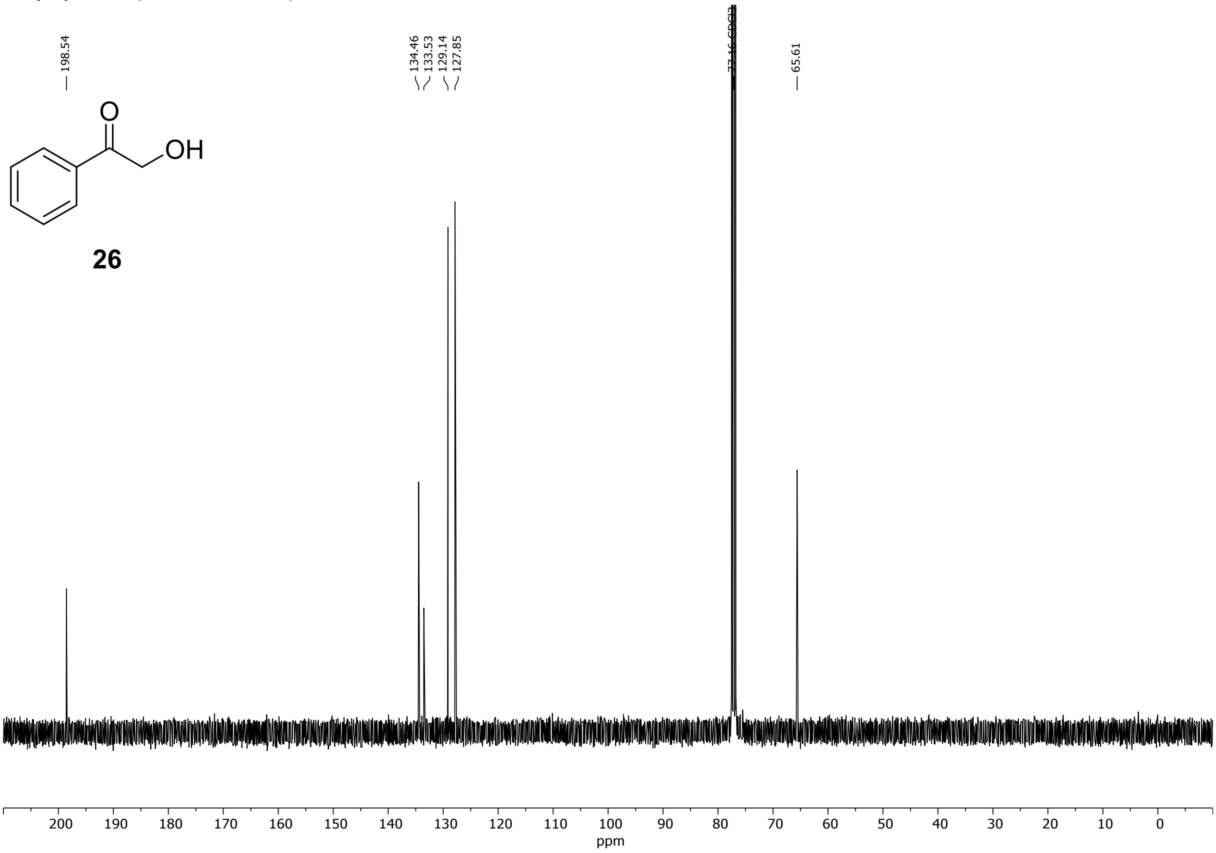

8-Azido-7-bromooctan-2-ol (28)

^{1}**H NMR** (400 MHz, $CDCl_3$)

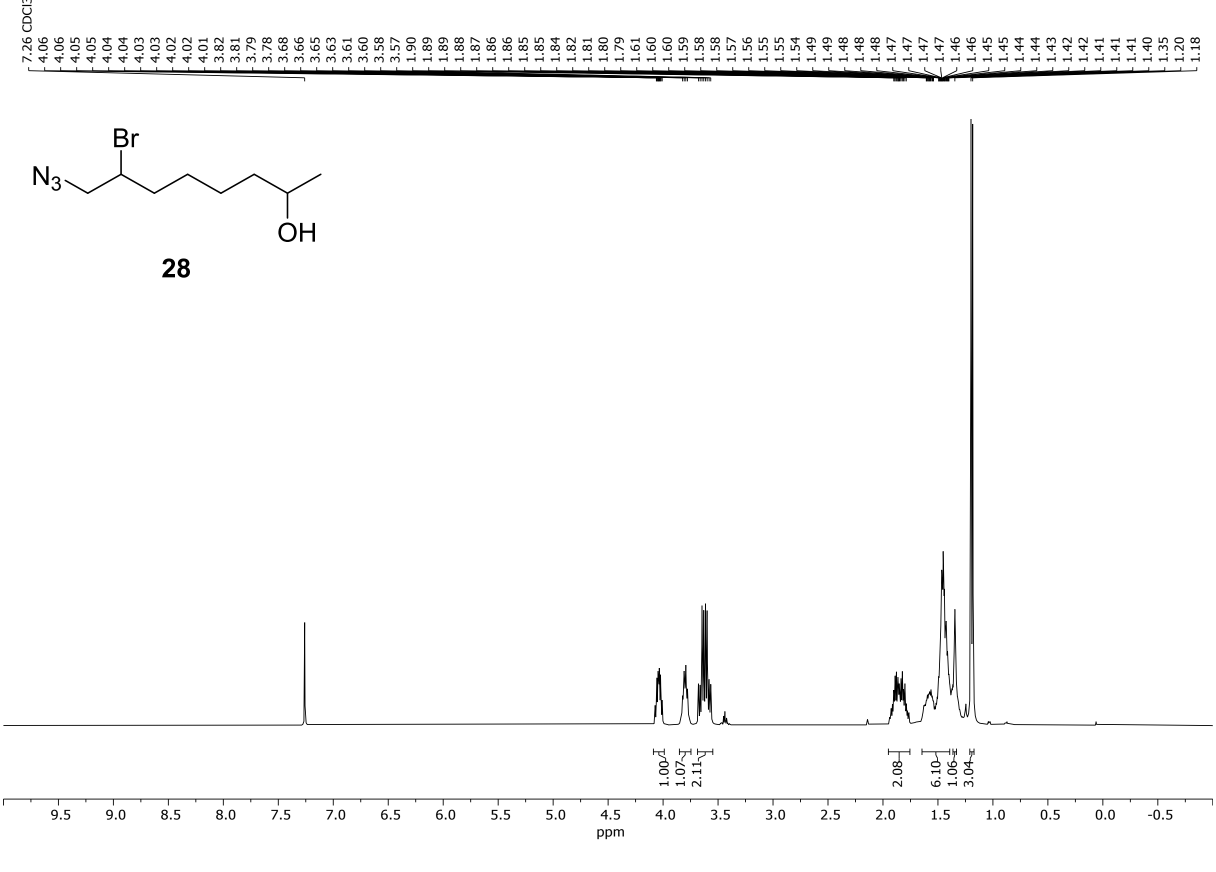

$^{13}C\{^{1}H\}$ **NMR** (101 MHz, $CDCl_3$)

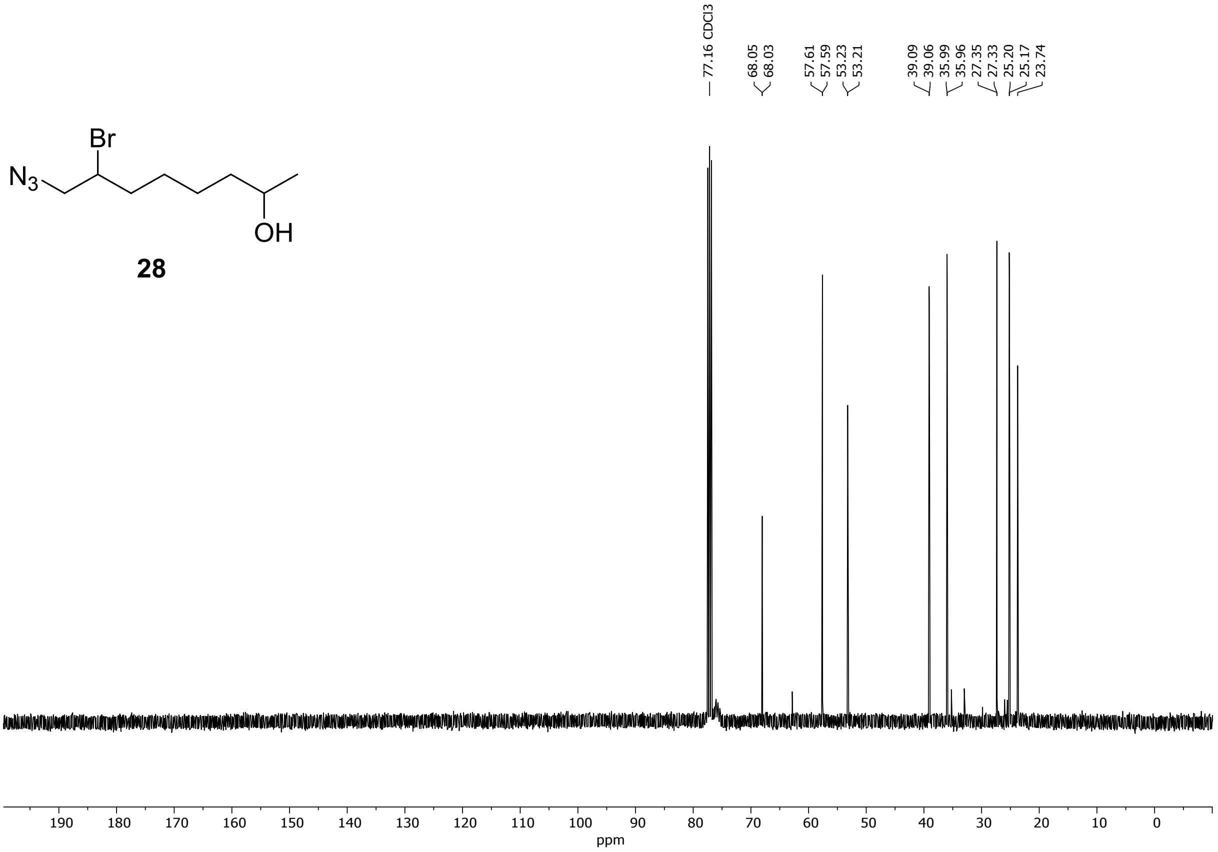

FT-IR

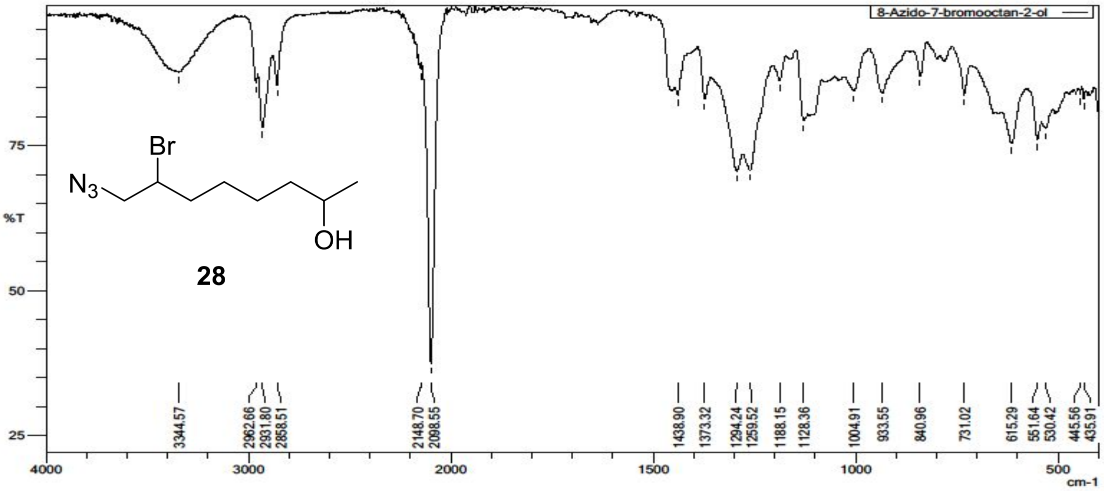

8-Azido-7-bromooctan-2-one (29)

^{1}H NMR (400 MHz, $CDCl_3$)

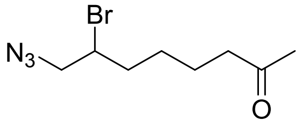

29

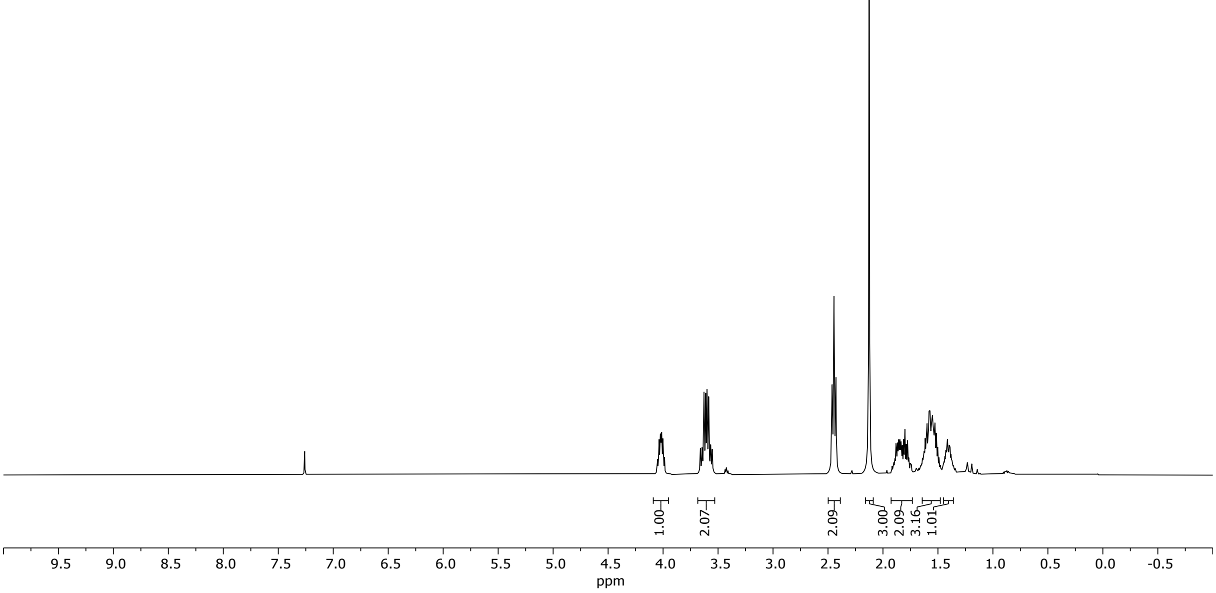

$^{13}C\{^1H\}$ NMR (101 MHz, $CDCl_3$)

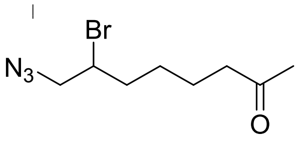

29

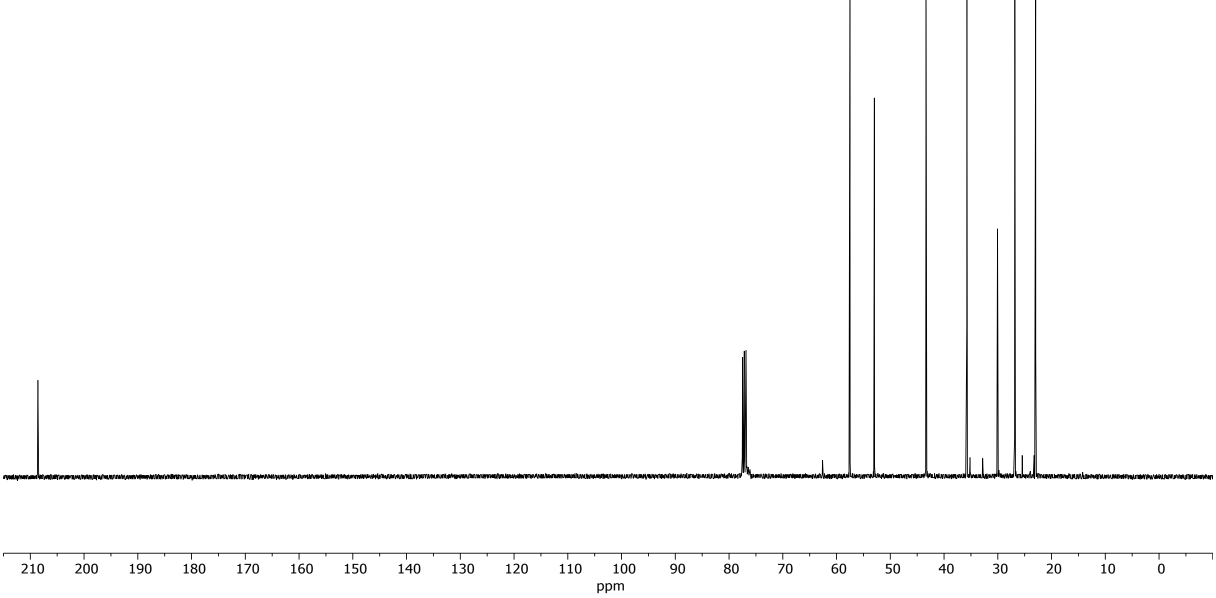

FT-IR

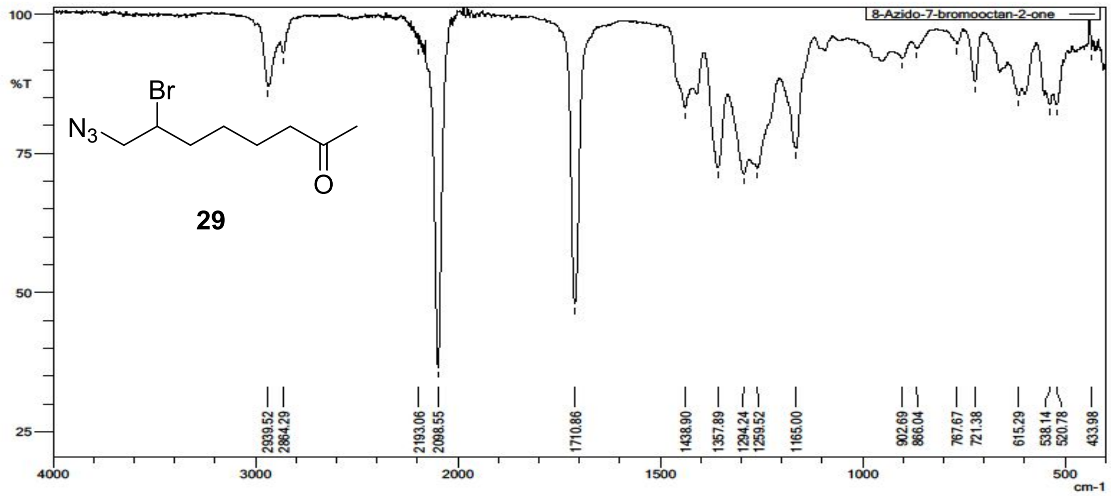

1-(4-(2-Azido-1-bromoethyl)phenyl)ethan-1-ol (31)

^{1}H NMR (400 MHz, $CDCl_3$)

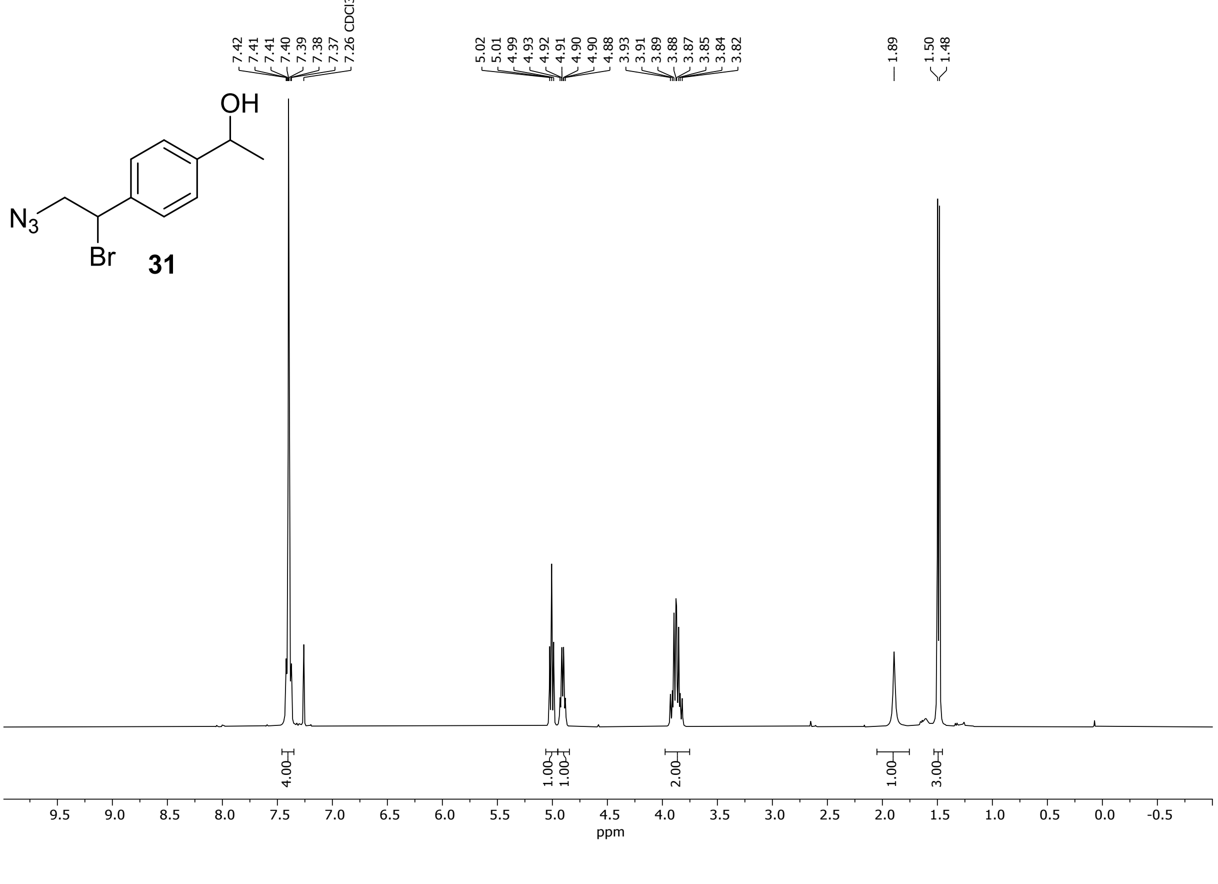

$^{13}C\{^1H\}$ NMR (101 MHz, $CDCl_3$)

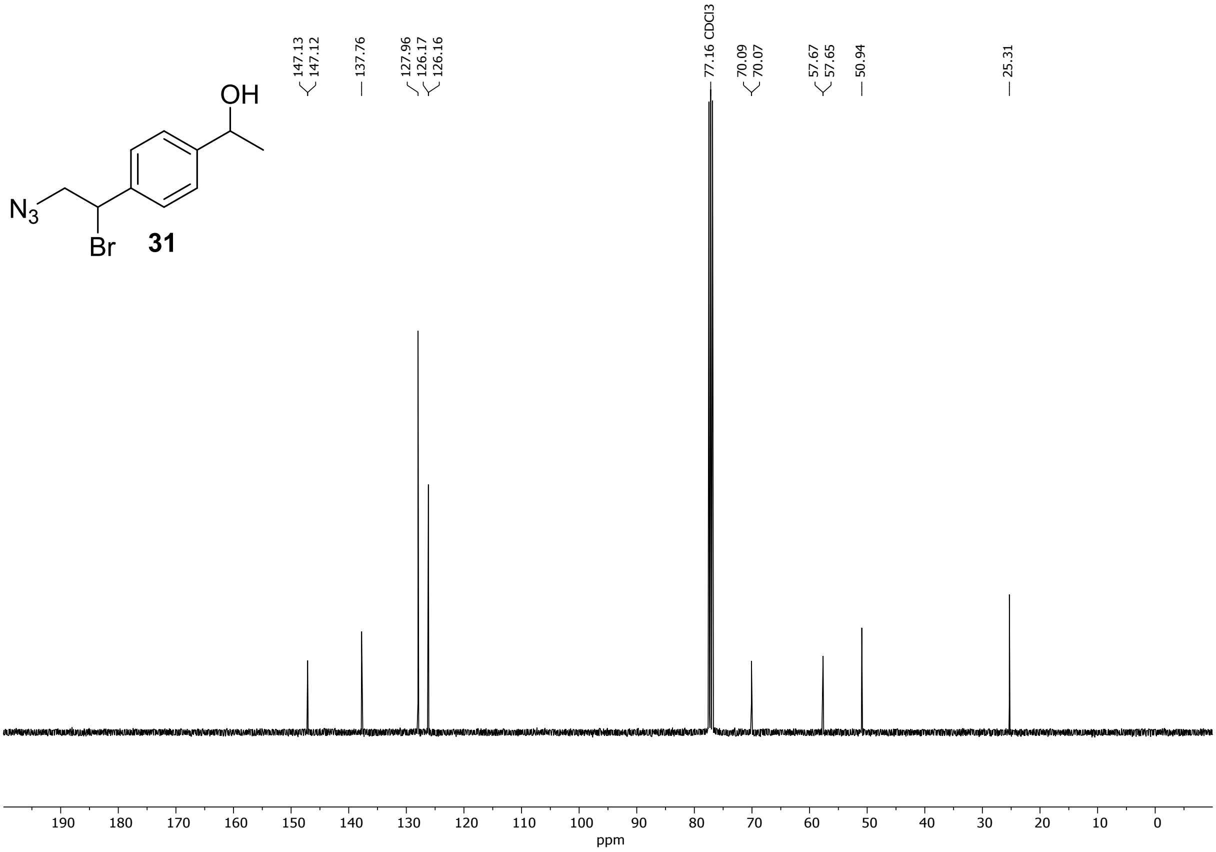

FT-IR

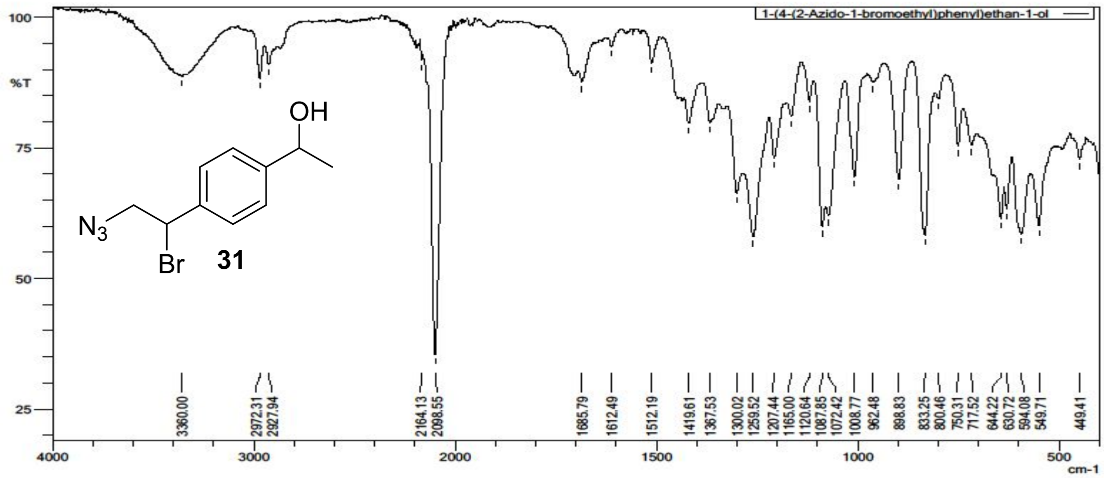

1-(4-(2-Azido-1-bromoethyl)phenyl)ethan-1-one (32)

^{1}H NMR (400 MHz, $CDCl_3$)

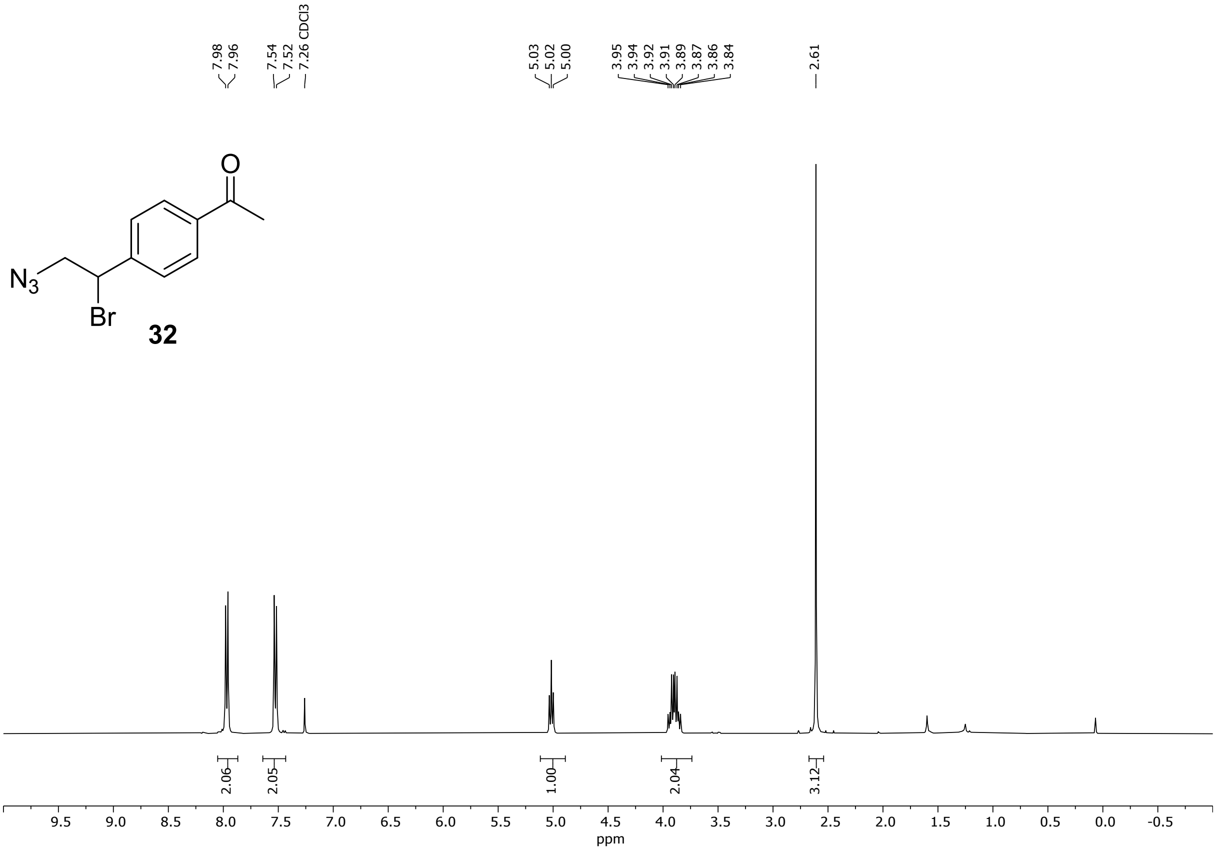

^{13}C{^{1}H} NMR (101 MHz, $CDCl_3$)

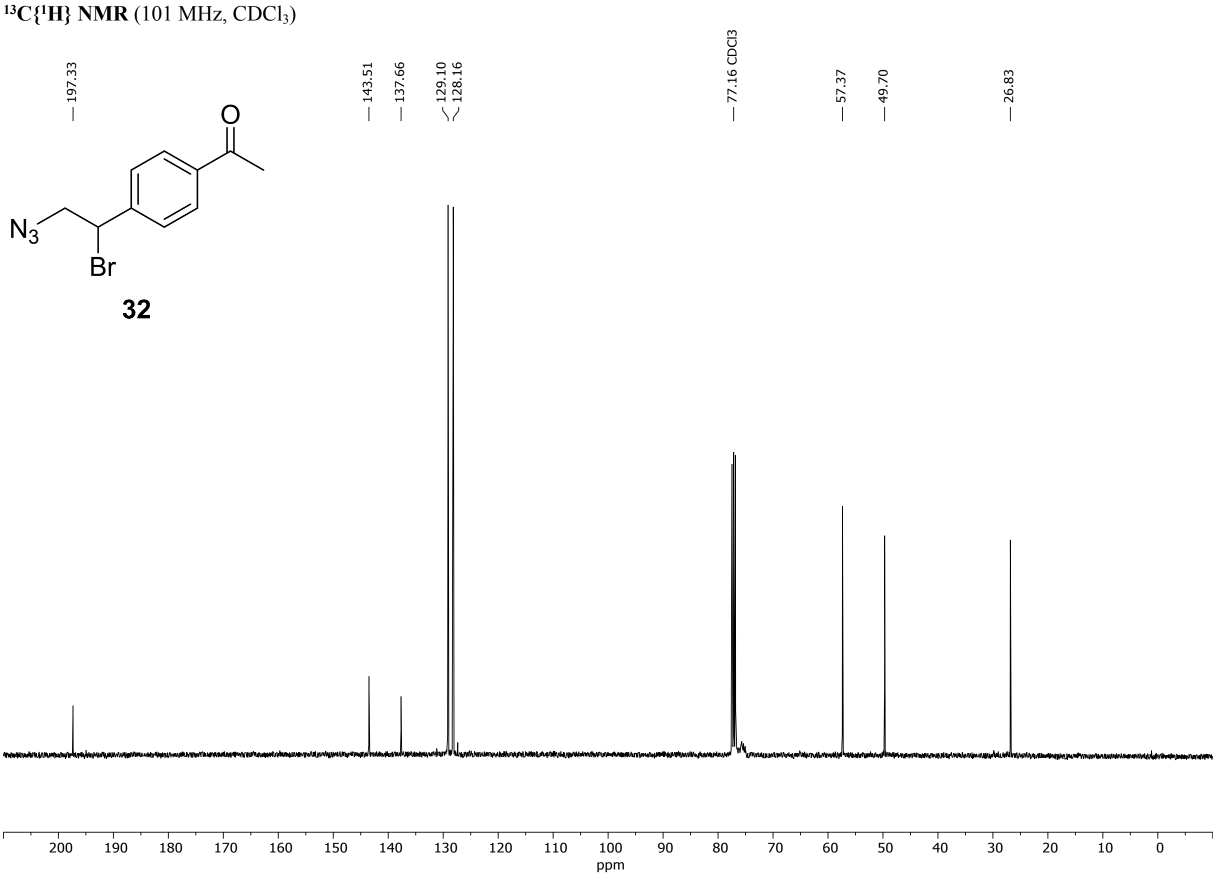

FT-IR

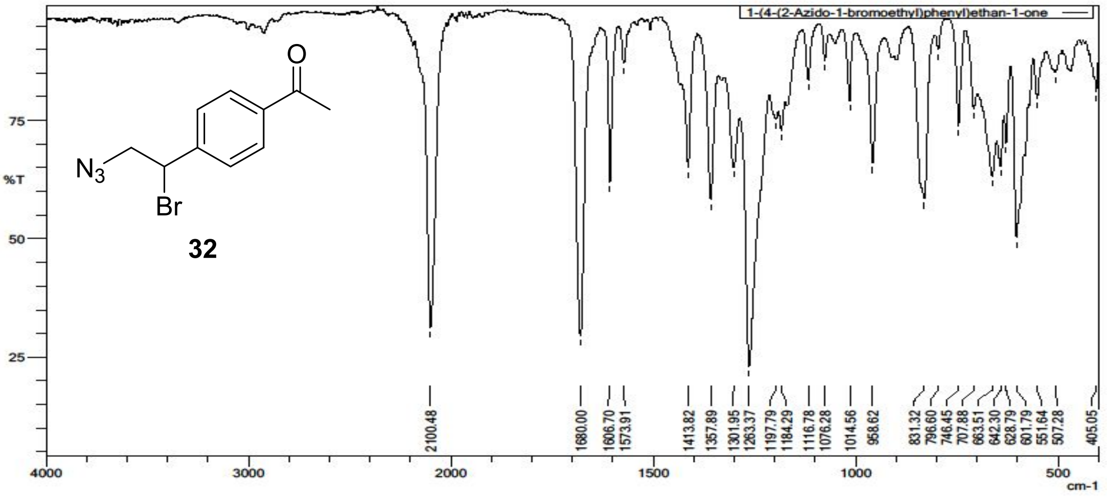
1-(4-(2-Azido-1-bromoethyl)phenyl)ethan-1-one
%T
75
50
25
O
N_3
Br
32
2100.48
1680.00
1606.70
1573.91
1413.82
1357.89
1301.95
1263.37
1197.79
1184.29
1116.78
1076.28
1014.56
958.62
831.32
796.60
746.45
707.88
663.51
642.30
628.79
601.79
551.64
507.28
405.05
4000
3000
2000
1500
1000
500
cm-1
